# Cancer incidence attributable to tuberculosis in 2015: global, regional, and national estimates

**DOI:** 10.1186/s12885-020-06891-5

**Published:** 2020-05-12

**Authors:** Chi Yan Leung, Hsi-Lan Huang, Md. Mizanur Rahman, Shuhei Nomura, Sarah Krull Abe, Eiko Saito, Kenji Shibuya

**Affiliations:** 1 Department of Global Health Policy, Graduate School of Medicine, The University of Tokyo, 7-3-1 Hongo, Bunkyo-ku, Tokyo, 113-0033 Japan; 2 Division of Cancer Statistics Integration, Center for Cancer Control and Information Services, National Cancer Center, Tokyo, Japan; 3 Department of Health Policy and Management, School of Medicine, Keio University, Tokyo, Japan; 4 Epidemiology and Prevention Group, Research Center for Cancer Prevention and Screening, National Cancer Center, Tokyo, Japan; 5 University Institute for Population Health, King’s College London, London, UK

**Keywords:** Tuberculosis, Cancer, Attributable fraction

## Abstract

**Background:**

Tuberculosis is associated with increased risk of cancer. However, the impact of tuberculosis on global cancer burden is unknown.

**Methods:**

We performed random-effects meta-analyses and meta-regressions of studies reporting the association between tuberculosis and cancer risks by searching PubMed, Web of Science, Embase, Cochrane library, and CINAHL from inception to 1 June 2019. Population attributable fractions (PAFs) of cancer incidence attributable to tuberculosis were calculated using relative risks from our meta-analyses and tuberculosis prevalence data from Global Health Data Exchange by age, sex, and country. The study has been registered with PROSPERO (CRD42016050691).

**Results:**

Fourty nine studies with 52,480 cancer cases met pre-specified inclusion criteria. Tuberculosis was associated with head and neck cancer (RR 2.64[95% CI 2.00–3.48]), hepatobiliary cancer (2.43[1.82–3.25]), Hodgkin’s lymphoma (2.19[1.62–2.97]), lung cancer (1.69[1.46–1.95]), gastrointestinal cancer (1.62[1.26–2.08]), non-Hodgkin’s lymphoma (1.61[1.34–1.94]), pancreatic cancer (1.58[1.28–1.96]), leukaemia (1.55[1.25–1.93]), kidney and bladder cancer (1.54[1.21–1.97]), and ovarian cancer (1.43[1.04–1.97]). We estimated that 2.33%(1.14–3.81) or 381,035(187145–623,404) of global cancer incidences in 2015 were attributable to tuberculosis. The PAFs varied by Socio-demographic Index (SDI)—ranging from 1.28% (0.57–2.31%) in the high-SDI countries to 3.51% (1.84–5.42%) in the middle-SDI countries. Individually, China and India accounted for 47% of all tuberculosis-related cancer cases.

**Conclusions:**

Tuberculosis is associated with increased risk of cancer at ten sites. The burden of tuberculosis attributable cancer skewed towards lower resource countries. Research priorities are to better understand regional disparities and underlying mechanism linking tuberculosis and cancer development.

## Background

In 2015, 17.5 million new cancer cases were reported worldwide, with 8.7 million cancer-related deaths [1]. Carcinogenic infections are well-established risk factors for cancer, namely Epstein-Barr virus, *Helicobacter pylori*, hepatitis B and C virus, human herpes virus type 8, and human papillomavirus [2]. In 2012, 2.2 million (15.4%) of global incident cancers were attributed to infections [2]. Substantial reduction of infection-related cancer burden has been made by prevention and treatment of infectious agents, for instance, hepatitis B virus vaccine and human papillomavirus vaccine [2].

Tuberculosis is the global leading cause of infectious disease mortality and the ninth leading cause of death in 2016 [3]. From 2000 to 2016, tuberculosis deaths fell from 1.7 million to 1.3 million, yet an estimated 10.4 million new tuberculosis cases arose in 2016 [3]. Although a growing body of evidence has revealed the association between tuberculosis and cancer, [4–10] the global cancer burden attributable to tuberculosis has not been quantified, and therefore, the potential impact of tuberculosis elimination on cancer burden remains unclear. Quantification of global cancer burden attributable to tuberculosis can contribute to the global and national discussions on health system investments, especially in countries facing the double burden of tuberculosis infection and cancer. In line with the Sustainable Development Goal (SDG) to end tuberculosis, this study aims to quantify the proportion of global cancer incidence in 2015 that was attributable to tuberculosis, and to explore additional potential benefits of tuberculosis elimination.

## Methods

### Overview

We performed a systematic review and meta-analysis to quantify the association of tuberculosis with the risk of cancers. To ensure that population attributable fractions (PAFs) were calculated using pooled risk estimates from sufficient studies, we defined tuberculosis-related cancers as those including more than five studies to synthesise risk estimates and having association with tuberculosis. Then, age-, sex-, and country-specific PAFs of tuberculosis-related cancers in 2015 were estimated using corresponding pooled relative risks assessed in our meta-analysis. We calculated the PAFs of cancer attributable to tuberculosis in 195 countries and aggregated into 11 geographical regions and five Socio-demographic Index (SDI) categories. This study adhered to the Preferred Reporting Items for Systematic Reviews and Meta-Analyses (PRISMA) guidelines and the Guideline for Accurate and Transparent Health Estimates Reporting (GATHER) (Additional file 1: PRISMA Checklist) [11, 12].

### Search strategy and selection criteria

We searched PubMed, Web of Science, Embase, Cochrane library, and CINAHL from inception to 1 June 2019, with no language restrictions, reporting the association between tuberculosis and risk of cancer at 17 sites (Additional file 2: Table S1–S5). In case of non-English articles, we consulted two native speakers for translations. The search strategy was iterative, in that the bibliographies of all included relevant studies were manually searched for additional articles. Two reviewers (CYL and HLH) independently conducted title and abstract screening of potentially eligible articles for inclusion. Disagreement on eligibility was resolved by discussion between the reviewers. We included all articles of original observational studies (cohort and case-control studies) which assessed the risk of cancer incidence at 17 sites in patients with tuberculosis compared to those without, starting at age of 20 years or older, and published in a peer-reviewed journal. To minimize potential publication bias, we excluded studies with a sample size of fewer than 50. We specified that each study must either provide relative risk (RR), odds ratio (OR), or hazard ratio (HR) with 95% confidence intervals (CIs); or provide sufficient data that would allow the risk estimate to be calculated. We excluded reviews, editorials, letters, and animal studies, along with studies assessing cancer mortality risk in tuberculosis infection. The review protocol was registered in PROSPERO (CRD42016050691).

### Data extraction and quality assessment

A standardised observation form (Additional file 2: Supplementary Notes) was independently completed and crosschecked by two reviewers (CYL and HLH) during data extraction. In cases where duplicated cohorts were reported in multiple studies, we extracted data from the study with the larger sample size or higher study quality with a lower risk of bias based on the Newcastle-Ottawa Scale (NOS) [13]. We assessed the methodological quality and risk of bias (Additional file 2: Supplementary Notes) in the selection, comparability, and outcome of all included studies using NOS by two independent reviewers (CYL and HLH) [13].

### Statistical analysis

We estimated pooled cancer-specific RRs with 95% CIs by random-effects meta-analysis with inverse-variance weighting. OR was converted to RR, [14] and the HR was presumably equivalent to RR. We used the adjusted risk ratio from each study unless otherwise specified. We re-ran random-effects meta-analysis for lung cancer with never-smokers only (Additional file 2: Supplementary Notes) to eliminate the possible confounding effect of smoking. We assessed heterogeneity using *I*^*2*^ statistic, where 25, 50, and 75% were the cut-off value for low, moderate, and high heterogeneity, respectively. To explore the source of heterogeneity, we performed random-effects meta-regression to investigate whether associations varied according to geographical region, mean age, quality assessment by Newcastle-Ottawa Scale, sample size, SDI, study design (cohort or case-control study), adjustment for confounding variables, and World Bank country-income category. Publication bias and small-study effects were assessed by visual inspection of funnel plots and Egger’s regression asymmetry test [15]. To address funnel plot asymmetry, we used the trim and fill method to evaluate the number of missing studies and their influence on the pooled estimates. For sensitivity analyses, random-effects models were re-run without highly influential studies, on the basis of weight estimates from meta-analysis. In this study, unless *P* < 0.0001, exact *p* values are provided.

### Tuberculosis attributable fractions

PAF is the proportion of cancer incidence that can be attributed to a risk factor in a given population [16]. We calculated the PAFs of tuberculosis-related cancers for each sex and age group (20–24, 25–29, 30–34, 35–39, 40–44, 45–49, 50–54, 55–59, 60–64, 65–69, 70–74, 75–79, 80–84, 85–89, and 90–94) in 195 countries for a binary exposure using the following equation: [16]

$$ \left(\mathrm{PAF}=\right)\ \frac{p\left( RR-1\right)}{1+p\left( RR-1\right)} $$

where *p* is the age- and sex-specific prevalence of tuberculosis in the given population; and *RR* is the pooled RR of tuberculosis-related cancers estimated in our meta-analyses. Age-, sex-, and country-specific tuberculosis prevalence estimates were derived from Global Health Data Exchange (GHDx) [17]. The case definition contains tuberculosis in all forms, including active tuberculosis and latent tuberculosis infection [17]. For PAF estimation of lung cancer, we restricted to use pooled RR which was adjusted for smoking status. We integrated the uncertainties of estimated RRs and tuberculosis prevalence to report the 95% CI for PAFs using the substitution method [18].

We estimated age-, sex-, country-, and cancer site-specific incident cancer cases attributable to tuberculosis infection by multiplying age-, sex-, country-, and cancer site-specific PAFs by corresponding cancer incident cases. We obtained information on age-, sex-, and country-specific cancer incidence from Global Health Data Exchange (GHDx) [17]. Countries and territories were grouped into 11 geographical regions and five SDI quintiles in 2015 (Additional file 2: Supplementary Notes). For regional-specific and SDI-specific PAFs for each cancer site, we divided the summation of individual national estimates of tuberculosis-related cancer incident cases by the total number of cancer incident cases in the corresponding category. The precise time required for the development of tuberculosis-related cancer is not well established. We assumed a lag-time of 15 years between first exposure and cancer diagnosis, which represents the average lag time for most risk factors and cancers [19]. Based on the assumption of lag-time, we mapped the tuberculosis prevalence in 2000 to cancer incidence in 2015. We used STATA version 14.2 (College Station, TX, USA) to analyse data.

## Results

**Fig. 1:**
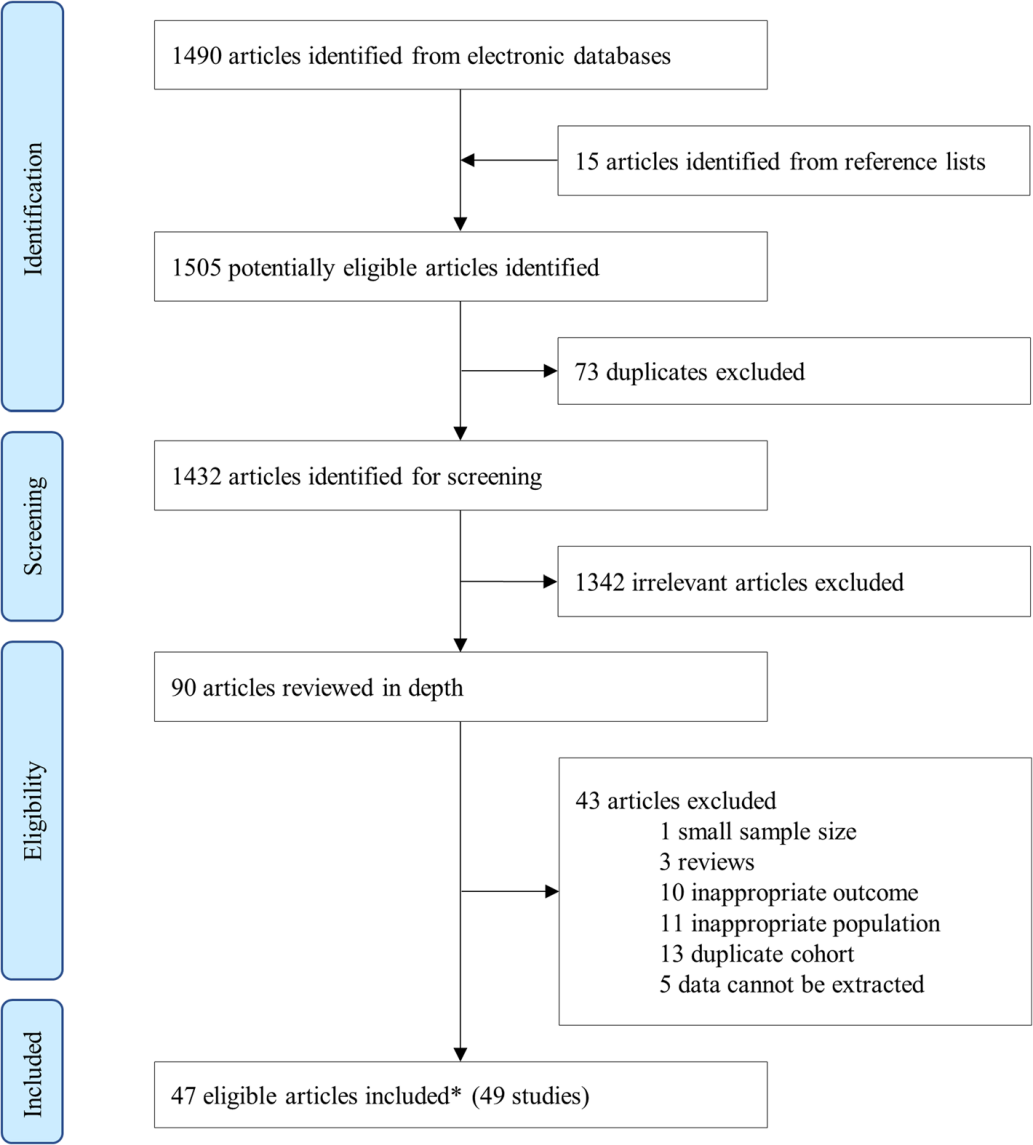
Study selection. ^#^Two articles reported two different independent study results within one article (see Additional file 2: Table S7)

Among 1505 articles identified, 90 were eligible for full-text review. Search details and process with reasons for exclusion are presented in Fig. 1 and Additional file 2 Table S6. A total of 47 published articles with 49 unique studies reporting on 52,480 cancer cases met the inclusion criteria, providing relevant data on lung cancer risk (38 studies, 40,062 cancer cases) and extrapulmonary cancer risks (13 studies, 12,418 cancer cases) (Additional file 2: Table S7). Overall, 11 of these studies were cohort studies and 38 were case-control studies. The studies were published between 1982 and 2017, with two-thirds (33/49) published after 2000. Eighteen in studies were conducted in Southeast Asia, East Asia, and Oceania; 14 studies in High-income North America; 11 in Western Europe; three in High-income Asia Pacific; and three in Central Europe, Eastern Europe, and Central Asia (Additional file 2: Fig. S1). Quality assessment suggested that 75% of articles (35/47) were at low risk of bias, whereas 5% (2/47) and 20% (10/47) were at medium or high risk of bias, respectively (Additional file 2: Table S8–9, and Fig. S2).

**Fig. 2:**
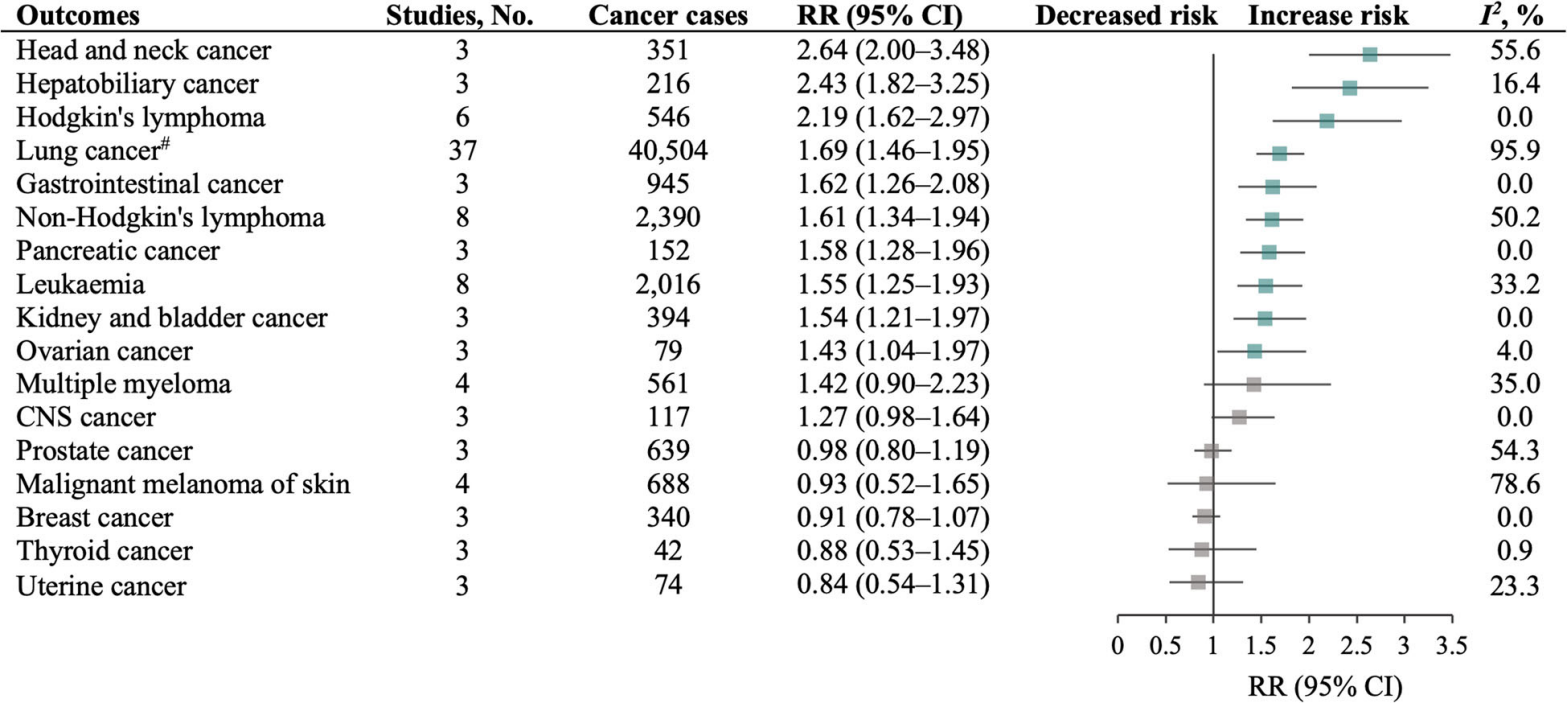
Summary of pooled relative risks for the association between tuberculosis and cancers. Note: ^#^Of 37 studies for lung cancer, 23 studies qualified the association between tuberculosis and lung cancer with adjustment for smoking, pooled relative risk (RR) (1.55 [95% CI 1.31–1.83], *I*^2^ = 96.0%); 14 studies qualified the association between tuberculosis and lung cancer risk among never-smokers, pooled RR (1.64 [1.41–1.91], *I*^2^ = 58.8%). Forest plots for each pooled estimate are shown in Additional file 2 Fig. S3–9. Blue indicates an increase in risk of cancer; grey indicates a null association. No.: number, RR: relative risk, CI: confidence interval, CNS: central nervous system.

The results from meta-analysis are shown in Fig. 2. Tuberculosis was associated with increased risk of cancer at ten sites: head and neck cancer (RR 2.64 [95% CI 2.00–3.48]), hepatobiliary cancer (2.43 [1.82–3.25]), Hodgkin’s lymphoma (2.19 [1.62–2.97]), lung cancer (1.69 [1.46–1.95]), gastrointestinal cancer (1.62 [1.26–2.08]), non–Hodgkin’s lymphoma (1.61 [1.34–1.94]), pancreatic cancer (1.58 [1.28–1.96]), leukaemia (1.55 [1.25–1.93]), kidney and bladder cancer (1.54 [1.21–1.97]), and ovarian cancer (1.43 [1.04–1.97]). The pooled RRs of lung cancer for smoking adjustment and for never-smokers were 1.55 (1.31–1.83) and 1.64 (1.41–1.91), respectively. On the other hand, there was no associations of tuberculosis with breast cancer, central nervous system cancer, cervical cancer, multiple myeloma, malignant melanoma of skin, prostate cancer, thyroid cancer, and uterine cancer. We observed high heterogeneity for lung cancer and malignant melanoma of skin (*I*^*2*^ = 95.9 and 78.6%, respectively). Forest plots for each cancer site were presented in appendix (Additional file 2: Fig. S3–9).

Meta-regression analyses (Additional file 2: Table S10–13) showed between-group differences by geographical region (*p* = 0.0305) and study design (*p* = 0.0227) for lung cancer, and these two variables explained 37% of between-study heterogeneity. Associations with tuberculosis were stronger in cohort studies than in case-control studies for leukaemia (*p* = 0.026) and non-Hodgkin’s lymphoma (*p* = 0.0317). Funnel plot asymmetry, which suggests the presence of publication bias and small-study effects, was not evident for lung cancer (Additional file 2: Fig. S10). The trim and fill method in a random-effects model suggested that overall estimates were not greatly modified by publication bias (Additional file 2: Table S14). Sensitivity analyses produced similar results, suggesting that results were robust to exclude highly influential studies (Additional file 2: Table S15).

Among the ten cancer sites identified, we further investigated the PAFs for cancers with pooled RRs obtained from more than five studies. Our results show that an estimated 2.33% (1.14–3.81%) or 381,035 (187145–623,404) of global cancer incidence in 2015 were attributable to tuberculosis infection if the association is causal. By sex, 2.93% (1.45–4.75%) of cancer incidence in 2015 in men and 1.61% (0.78–2.67%) in women were attributable to tuberculosis worldwide (Table 1). PAFs of tuberculosis-related cancers varied by geographical region, SDI, and cancer site. Table 1 shows the regional PAFs, with the highest PAF of 3.99% (2.1–6.13) in the Southeast Asia, East Asia, and Oceania, and the lowest PAF of 0.76% (0.31–1.45) in Australasia. SDI-specific estimates showed that middle-SDI countries had the highest PAF, at 3.51% (1.84–5.42) of total cancer, while countries with high SDIs had the lowest PAF, at 1.28% (0.57–2.31) of total cancer (Table 1). Cancer site-specific estimates varied from 12.59% (6.07–21.15) for non-Hodgkin’s lymphoma to 22.27% (10.62–36.44) for Hodgkin’s lymphoma.

**Table 1:** Estimated PAFs and numbers of cancer cases associated with tuberculosis in 2015, by SDI and region

	Hodgkin’s lymphoma		Leukaemia		Lung cancer		Non-Hodgkin’s lymphoma		Total		
	PAF (95% CI)	n (95% CI)	PAF (95% CI)	n (95% CI)	PAF (95% CI)	n (95% CI)	PAF (95% CI)	n (95% CI)	n (95% CI)	PAF TB-related^a^ (95% CI)	PAF all^b^ (95% CI)
**Male**											
**Region**											
Australasia	13.23% (5.40–25.08)	64 (26–121)	6.67% (2.41–13.29)	287 (104–572)	6.76% (3.01–12.20)	610 (271–1100)	7.58% (3.30–13.99)	297 (129–548)	1258 (530–2341)	7.10% (2.99–13.21)	0.74% (0.31–1.38)
Central Europe, Eastern Europe, and Central Asia	29.43% (14.41–46.50)	1314 (644–2077)	16.31% (6.60–28.78)	3001 (1215–5295)	16.86% (8.37–27.31)	20 480 (10 163–33 171)	18.12% (8.90–29.66)	2501 (1228–4093)	27 297 (13 249–44 636)	17.26% (8.38–28.23)	3.72% (1.80–6.08)
High-income Asia Pacific	17.62% (8.18–30.16)	176 (82–302)	8.79% (3.50–16.16)	876 (349–1610)	8.49% (4.23–13.98)	8029 (3997–13 217)	9.63% (4.70–16.22)	1672 (816–2817)	10 754 (5243–17 946)	8.75% (4.27–14.60)	1.75% (0.85–2.92)
High-income North America	9.64% (4.55–16.84)	711 (336–1243)	5.32% (2.18–9.69)	1699 (694–3093)	5.45% (2.75–8.93)	7954 (4013–13 030)	5.93% (2.96–9.92)	3062 (1526–5118)	13 427 (6570–22 484)	5.67% (2.77–9.50)	0.98% (0.48–1.65)
Latin America and Caribbean	29.58% (14.50–46.63)	576 (283–909)	15.89% (6.34–28.34)	1796 (716–3202)	16.40% (8.12–26.56)	6123 (3033–9920)	17.98% (8.76–29.56)	1721 (838–2830)	10 216 (4869–16 860)	16.98% (8.09–28.03)	1.95% (0.93–3.22)
North Africa and Middle East	30.03% (12.49–50.03)	730 (304–1216)	16.41% (5.52–31.65)	1941 (652–3743)	16.75% (6.81–29.95)	6935 (2822–12 404)	18.36% (7.44–32.83)	1549 (628–2769)	11 155 (4406–20 132)	17.40% (6.87–31.40)	4.48% (1.77–8.09)
South Asia	34.35% (18.25–51.17)	1582 (841–2358)	19.85% (8.60–33.10)	3696 (1602–6165)	17.54% (9.01–27.69)	14 416 (7410–22 759)	20.89% (10.76–32.93)	3895 (2007–6140)	23 590 (11 860–37 421)	19.01% (9.56–30.16)	3.66% (1.84–5.80)
Southern Latin America	13.79% (5.72–25.91)	45 (19–84)	6.96% (2.49–14.00)	132 (47–266)	7.50% (3.30–13.64)	760 (335–1382)	8.02% (3.48–14.91)	162 (70–301)	1099 (471–2032)	7.64% (3.28–14.14)	1.26% (0.54–2.33)
Southeast Asia, East Asia, and Oceania	32.63% (17.29–48.60)	2122 (1125–3162)	18.24% (7.96–30.36)	11 435 (4992–19 031)	18.50% (10.00–28.00)	108 968 (58 888–164 937)	20.09% (10.65–30.93)	10 297 (5458–15 853)	132 824 (70 462–202 983)	18.72% (9.93–28.61)	4.81% (2.55–7.35)
Sub-Saharan Africa	33.74% (15.76–53.15)	600 (280–945)	19.72% (7.46–35.48)	1170 (443–2106)	21.91% (10.60–35.22)	3879 (1877–6236)	20.69% (9.41–34.92)	1673 (761–2824)	7322 (3361–12 111)	21.85% (10.03–36.15)	2.84% (1.30–4.69)
Western Europe	14.93% (5.49–29.95)	1127 (414–2262)	7.36% (2.38–15.94)	2986 (965–6473)	7.83% (3.05–15.61)	14 577 (5689–29 080)	8.69% (3.37–17.49)	4374 (1695–8802)	23 065 (8764–46 616)	8.10% (3.08–16.37)	1.49% (0.57–3.01)
**SDI**											
High	13.82% (5.89–25.40)	2314 (986–4254)	7.63% (2.84–14.89)	6843 (2546–13 349)	8.02% (3.69–14.15)	35 888 (16 528–63 322)	8.19% (3.69–14.79)	10 130 (4572–18 296)	55 176 (2463–99 221)	8.14% (3.64–14.64)	1.47% (0.66–2.64)
High-middle	25.87% (12.18–42.31)	1485 (699–2428)	13.38% (5.13–24.72)	3415 (1310–6312)	14.47% (6.87–24.34)	22 283 (10 576–37 483)	14.44% (6.65–24.94)	3165 (1458–5466)	30 347 (14 043–51 689)	14.65% (6.78–24.95)	3.13% (1.45–5.33)
Middle	31.79% (16.41–48.19)	2756 (1423–4177)	17.87% (7.72–29.99)	12 897 (5571–21 648)	18.40% (9.94–27.85)	111 839 (60 432–169 265)	19.69% (10.32–30.60)	11 689 (6126–18 160)	139 181 (73 551–213 250)	18.61% (9.83–28.51)	4.31% (2.28–6.60)
Low-middle	34.55% (17.63–52.26)	2069 (1056–3130)	19.75% (8.09–34.04)	5071 (2077–8741)	17.95% (8.76–29.17)	20 436 (9972–33 216)	21.00% (10.35–33.91)	5053 (2489–8157)	32 629 (15 594–53 244)	19.24% (9.19–31.39)	3.86% (1.84–6.30)
Low	31.77% (14.12–51.56)	426 (189–691)	17.97% (6.24–34.08)	793 (275–1504)	19.01% (8.22–32.84)	2285 (988–3948)	19.58% (8.58–33.85)	1167 (511–2017)	4671 (1964–8160)	19.68% (8.28–34.38)	3.08% (1.30–5.38)
**Global**											
	23.52% (11.31–38.15)	9049 (4352–14 679)	13.35% (5.42–23.71)	29 020 (11 779–51 554)	14.44% (7.38–23.01)	192 732 (98 497–307 235)	13.28% (6.45–22.17)	31 204 (15 157–52 096)	262 005 (129 785–425 563)	14.35% (7.11–23.31)	2.93% (1.45–4.75)
**Female**											
**Region**											
Australasia	12.46% (4.80–24.65)	43 (16–85)	6.47% (2.17–13.65)	149 (50–314)	6.75% (2.78–12.94)	415 (171–796)	7.39% (3.01–14.36)	216 (88–420)	823 (325–1614)	7.02% (2.78–13.77)	0.79% (0.31–1.55)
Central Europe, Eastern Europe, and Central Asia	27.31% (13.14–43.90)	961 (462–1544)	15.41% (6.23–27.24)	2199 (890–3887)	15.36% (7.61–24.98)	5585 (2767–9078)	17.12% (8.41–28.03)	2451 (1204–4012)	11 195 (5324–18 522)	16.35% (7.78–27.06)	1.53% (0.73–2.52)
High-income Asia Pacific	18.95% (8.85–32.08)	96 (45–163)	9.69% (3.88–17.64)	812 (325–1477)	9.04% (4.51–14.81)	3735 (1865–6120)	10.59% (5.20–17.68)	1522 (747–2541)	6165 (2982–10 301)	9.55% (4.62–15.95)	1.39% (0.67–2.32)
High-income North America	6.91% (3.21–12.36)	381 (177–682)	3.82% (1.55–7.05)	813 (329–1502)	4.00% (2.00–6.64)	5054 (2528–8391)	4.32% (2.14–7.31)	1812 (897–3066)	8060 (3931–13 640)	4.13% (2.01–6.99)	0.69% (0.34–1.17)
Latin America and Caribbean	28.41% (13.84–45.13)	367 (179–583)	15.62% (6.18–28.02)	1422 (563–2551)	15.61% (7.67–25.53)	3833 (1883–6267)	17.37% (8.46–28.62)	1439 (701–2371)	7062 (3326–11 773)	16.33% (7.69–27.23)	1.34% (0.63–2.24)
North Africa and Middle East	27.74% (11.27–47.52)	429 (174–735)	16.42% (5.50–31.81)	1311 (440–2541)	16.25% (6.84–28.76)	1845 (777–3266)	17.58% (7.04–31.82)	1045 (419–1891)	4630 (1810–8433)	17.26% (6.74–31.43)	2.06% (0.81–3.75)
South Asia	32.14% (17.03–48.27)	747 (396–1123)	18.04% (7.68–30.65)	2478 (1055–4211)	16.66% (8.63–26.14)	4404 (2282–6908)	19.23% (9.79–30.71)	2417 (1230–3860)	10 046 (4963–16 101)	18.24% (9.01–29.24)	1.49% (0.74–2.39)
Southern Latin America	13.07% (5.05–25.65)	27 (11–54)	6.72% (2.23–14.22)	89 (29–187)	7.33% (2.99–14.04)	381 (155–731)	7.88% (3.19–15.31)	137 (56–266)	634 (251–1238)	7.49% (2.96–14.62)	0.75% (0.30–1.47)
Southeast Asia, East Asia, and Oceania	30.04% (15.59–45.75)	836 (434–1273)	16.77% (7.18–28.45)	6933 (2971–11 764)	17.00% (9.06–26.12)	40 170 (21 405–61 729)	18.51% (9.65–28.99)	5735 (2990–8984)	53 674 (27 799–83 751)	17.23% (8.92–26.89)	2.80% (1.45–4.37)
Sub-Saharan Africa	31.74% (14.70–50.86)	410 (190–658)	19.02% (7.19–34.45)	924 (349–1674)	20.29% (9.73–33.07)	1710 (820–2787)	19.62% (8.91–33.41)	1333 (605–2270)	4378 (1965–7389)	20.48% (9.19–34.57)	1.38% (0.62–2.33)
Western Europe	13.01% (4.80–26.43)	663 (244–1347)	6.24% (2.01–13.60)	1904 (615–4154)	7.10% (2.77–14.13)	6579 (2572–13 106)	7.59% (2.95–15.28)	3217 (1252–6474)	12 363 (4684–25 080)	7.24% (2.74–14.69)	1.02% (0.39–2.07)
**SDI**											
High	11.58% (4.90–21.55)	1376 (582–2562)	6.76% (2.51–13.27)	4369 (1619–8573)	6.55% (3.02–11.56)	18 126 (8360–32 015)	7.31% (3.30–13.22)	7522 (3394–13 601)	31 393 (13 955–56 751)	6.88% (3.06–12.44)	1.05% (0.46–1.89)
High-middle	24.57% (11.42–40.66)	1010 (469–1671)	12.89% (4.96–23.81)	2382 (916–4400)	13.11% (6.19–22.22)	5756 (2716–9756)	13.83% (6.42–23.82)	2713 (1259–4672)	11 861 (5360–20 499)	13.77% (6.22–23.81)	1.31% (0.59–2.26)
Middle	29.19% (14.71–45.32)	1218 (614–1892)	16.55% (7.04–28.24)	8030 (3415–13 699)	16.90% (8.99–26.02)	42 175 (22 426–64 916)	18.15% (9.35–28.69)	6853 (3531–10 831)	58 277 (29 986–91 338)	17.14% (8.82–26.87)	2.44% (1.25–3.82)
Low-middle	32.56% (16.40–49.96)	1041 (524–1597)	18.24% (7.32–32.04)	3557 (1428–6251)	16.88% (8.35–27.28)	6637 (3281–10 726)	19.54% (9.45–32.07)	3319 (1605–5447)	14 555 (6838–24 021)	18.42% (8.66–30.40)	1.62% (0.76–2.68)
Low	29.76% (13.10–49.23)	316 (139–523)	17.17% (5.88–33.11)	695 (238–1340)	17.84% (7.77–31.00)	1016 (443–1766)	18.45% (8.06–32.22)	917 (401–1602)	2945 (1221–5232)	18.66% (7.74–33.15)	1.45% (0.60–2.58)
**Global**											
	20.31% (9.53–33.75)	4961 (2328–8245)	12.27% (4.91–22.09)	19 033 (7617–34 263)	11.98% (6.05–19.37)	73 711 (37 226–119 179)	11.70% (5.59–19.84)	21 325 (1018–36 154)	119 030 (57 360–197 841)	12.18% (5.87–20.25)	1.61% (0.78–2.67)
**Both sexes**											
**Region**											
Australasia	12.91% (5.15–24.90)	107 (43–206)	6.60% (2.33–13.42)	436 (154–886)	6.76% (2.92–12.50)	1025 (442–1895)	7.50% (3.17–14.15)	513 (217–968)	2080 (855–3955)	7.07% (2.91–13.44)	0.76% (0.31–1.45)
Central Europe, Eastern Europe, and Central Asia	28.50% (13.85–45.35)	2275 (1106–3621)	15.92% (6.44–28.10)	5200 (2105–9182)	16.52% (8.19–26.77)	26 065 (12 930–42 249)	17.61% (8.65–28.83)	4952 (2433–8106)	38 492 (18 573–63 158)	16.99% (8.20–27.87)	2.62% (1.26–4.30)
High-income Asia Pacific	18.07% (8.41–30.80)	273 (127–465)	9.20% (3.67–16.84)	1688 (673–3088)	8.66% (4.31–14.23)	11 764 (5861–19 337)	10.06% (4.93–16.88)	3194 (1563–5358)	16 918 (8225–28 247)	9.02% (4.39–15.07)	1.60% (0.78–2.67)
High-income North America	8.47% (3.98–14.93)	1092 (513–1925)	4.72% (1.92–8.63)	2512 (1024–4594)	4.78% (2.40–7.87)	13 008 (6541–21 420)	5.21% (2.59–8.75)	4874 (2424–8184)	21 487 (10 501–36 124)	4.97% (2.43–8.36)	0.85% (0.41–1.43)
Latin America and Caribbean	29.11% (14.23–46.03)	944 (461–1492)	15.77% (6.27–28.20)	3218 (1279–5754)	16.09% (7.94–26.15)	9957 (4916–16 187)	17.70% (8.62–29.12)	3160 (1539–5201)	17 278 (8195–28 633)	16.71% (7.93–27.69)	1.65% (0.78–2.73)
North Africa and Middle East	29.14% (12.01–49.06)	1159 (478–1951)	16.41% (5.51–31.71)	3252 (1092–6284)	16.64% (6.82–29.69)	8780 (3599–15 670)	18.04% (7.28–32.41)	2594 (1047–4660)	15 785 (6216–28 565)	17.36% (6.84–31.41)	3.33% (1.31–6.03)
South Asia	33.61% (17.84–50.20)	2330 (1237–3480)	19.08% (8.21–32.06)	6174 (2657–10 376)	17.32% (8.92–27.31)	18 820 (9692–29 667)	20.22% (10.37–32.04)	6312 (3237–10 000)	33 636 (16 823–53 523)	18.78% (9.39–29.88)	2.55% (1.27–4.06)
Southern Latin America	13.50% (5.46–25.81)	72 (29–138)	6.86% (2.39–14.09)	221 (77–453)	7.44% (3.20–13.78)	1141 (490–2113)	7.96% (3.34–15.09)	299 (126–567)	1733 (722–3270)	7.59% (3.16–14.32)	1.01% (0.42–1.91)
Southeast Asia, East Asia, and Oceania	31.85% (16.78–47.75)	2958 (1559–4435)	17.66% (7.65–29.60)	18 368 (7963–30 795)	18.07% (9.73–27.46)	149 139 (80 292–226 666)	19.50% (10.27–30.20)	16 033 (8448–24 837)	186 498 (98 261–286 733)	18.27% (9.62–28.08)	3.99% (2.10–6.13)
Sub-Saharan Africa	32.90% (15.31–52.19)	1010 (470–1602)	19.40% (7.34–35.01)	2095 (792–3780)	21.38% (10.32–34.53)	5589 (2698–9023)	20.20% (9.18–34.23)	3006 (1366–5094)	11 700 (5326–19 500)	21.32% (9.70–35.53)	2.03% (0.92–3.39)
Western Europe	14.16% (5.21–28.53)	1790 (659–3609)	6.87% (2.22–14.94)	4891 (1581–10 626)	7.58% (2.96–15.12)	21 156 (8261–42 186)	8.19% (3.18–16.48)	7591 (2947–15 275)	35 428 (13 448–71 697)	7.78% (2.95–15.74)	1.28% (0.49–2.60)
**SDI**											
High	12.89% (5.48–23.80)	3690 (1568–6815)	7.27% (2.70–14.21)	11 212 (4166–21 922)	7.46% (3.44–13.16)	54 014 (24 889–95 337)	7.79% (3.51–14.07)	17 652 (7966–31 897)	86 568 (38 588–155 972)	7.63% (3.40–13.76)	1.28% (0.57–2.31)
High-middle	25.33% (11.86–41.62)	2494 (1168–4099)	13.17% (5.06–24.34)	5796 (2226–10 711)	14.17% (6.72–23.87)	28 039 (13 292–47 239)	14.15% (6.54–24.41)	5878 (2717–10 138)	42 208 (19 403–72 188)	14.39% (6.62–24.61)	2.25% (1.03–3.85)
Middle	30.95% (15.86–47.26)	3974 (2036–6069)	17.34% (7.44–29.28)	20 928 (8986–35 348)	17.97% (9.67–27.32)	154 014 (82 858–234 181)	19.09% (9.94–29.85)	18 542 (9657–28 991)	197 458 (103 537–304 588)	18.15% (9.52–28.00)	3.51% (1.84–5.42)
Low-middle	33.86% (17.21–51.46)	3110 (1580–4727)	19.10% (7.76–33.18)	8629 (3505–14 992)	17.67% (8.65–28.68)	27 074 (13 253–43 942)	20.40% (9.97–33.15)	8372 (4094–13 604)	47 184 (22 432–77 265)	18.98% (9.02–31.08)	2.71% (1.29–4.43)
Low	30.88% (13.67–50.53)	742 (328–1214)	17.59% (6.07–33.61)	1489 (514–2845)	18.63% (8.08–32.25)	3302 (1431–5714)	19.07% (8.35–33.11)	2084 (912–3619)	7616 (3185–13 392)	19.27% (8.06–33.89)	2.15% (0.90–3.78)
**Global**											
	22.27% (10.62–36.44)	14 010 (6681–22 923)	12.90% (5.21–23.03)	48 053 (1939–85 817)	13.66% (6.96–21.86)	266 442 (135 723–426 414)	12.59% (6.07–21.15)	52 529 (25 346–88 250)	381 035 (187 145–623 404)	13.59% (6.68–22.24)	2.33% (1.14–3.81)

Note: *PAF* population attributable fraction, n: number of cancer cases, *TB* tuberculosis, *n* number, *CI* confidence interval, *SDI* Socio-demographic Index

^a^PAF TB-related: proportion of TB-related cancers attributable to tuberculosis

^b^PAF all: proportion of all cancer attributable to tuberculosis

**Fig. 3:**
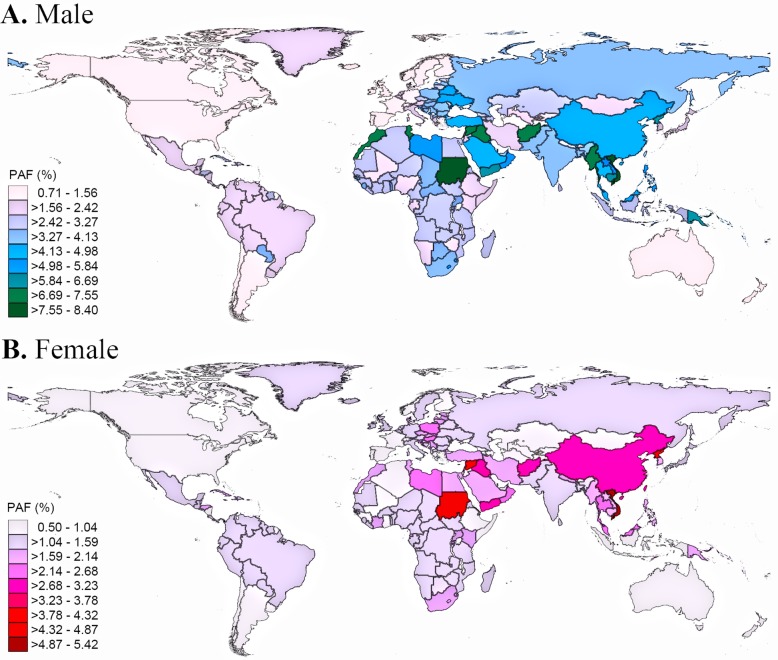
Proportion of cancer in 2015 attributable to tuberculosis in male (**a**) and female (**b**), by country.PAF: population attributable fraction. The figures were created using StataCorp. 2015. *Stata Statistical Software: Release 14*. College Station, TX: StataCorp LP.

Country-specific PAFs are presented in Fig. 3 and Additional file 2 Table S16. Of the 195 countries we analysed, the PAFs were higher for men than for women in all countries. In men, the PAFs were more than 7.2% in Morocco, Sudan, and Vietnam; but less than 1.0% in Australia, Chile, and the United States. In women, the PAFs were more than 4.5% in North Korea, Sudan, and Vietnam; but less than 0.6% in Jordan, Malta, and Spain. With respect to the national contribution to tuberculosis-related cancer cases in 2015 (Additional file 2: Table S17), China (153,259 cases [95% CI 83601–230,298]), India (25,457 [13341–38,736]), the United States (19,459 [9532–32,647]), Russia (14,572 [7108–23,676]), and Japan (12,801 [6346–21,111]) contributed the most. Two of the top five countries with the highest TB-related new cancer cases were among the three high tuberculosis burden countries listed by the WHO, namely China, and India, accounted for 47% of tuberculosis-related cancer cases worldwide. When PAFs for lung cancer were adjusted for smoking status, we observed 0.34–3.72% point difference with comparison to unadjusted PAFs (Additional file 2: Table S18). Since study design is a significant source of heterogeneity for lung cancer and leukaemia, we performed sensitivity analysis to calculate the PAFs using cohort studies exclusively (Additional file 2: Table S19, page 45–47). Compared with estimates in primary analysis, we observed 5.13–15.96 points difference for lung cancer and 3.67–15.31 points difference for leukaemia.

## Discussion

To our knowledge, this study is the first comprehensive assessment to estimate the impact of tuberculosis on global cancer incidence. We performed a systematic review and meta-analysis, synthesising non-overlapping data from 52,480 cancer patients from 49 studies, to quantify the association between tuberculosis and cancer incidence at 17 cancer sites. The study findings show that tuberculosis is associated with increased risk of cancer at ten sites in adults. Our estimates show that 2.93% (1.45–4.75%) of total cancer in men and 1.61% (0.78–2.67%) in women could be attributed to tuberculosis in 195 countries and territories in 2015.

This study adds important vision to the contribution of infectious agents to cancer risk. Previous study has quantified the global cancer burden attributable to nine infectious agents: *Helicobacter pylori*, human papillomavirus, hepatitis B virus, hepatitis C virus, Epstein-Barr virus, human herpesvirus type 8, *Schistosoma haematobium*, Human T-cell lymphotropic virus type 1, and *Opisthorchi viverrini* [2]. This study is the first estimate of global cancer incidence attributable to tuberculosis infection. The study findings are consistent with and also extend the preceding view on the association between tuberculosis and cancer risk. One previous study estimated the PAF of lung cancer attributable to tuberculosis with 1.1%, 2.4, and 12.7% in North America, Europe, and China, respectively [20]. This study assessed the PAF for three additional cancer sites and provides sex-, and country-specific estimates with uncertainty intervals and extensive sensitivity analyses compared to previous work. Collectively, our results provide robust estimates derived from a comprehensive search without language restriction and subgroup analyses. Also, we rigorously used the highest quality cancer incidence and tuberculosis prevalence data available.

Chronic inflammation fosters multiple tumour-promoting responses and seeds neoplastic microenvironments [21]. Experimental evidence showed that chronic tuberculosis infection in lung is sufficient to drive carcinogenesis [6]. Genome alteration with DNA damage led by oxidative stress was observed in tuberculosis-infected macrophages 2 months after initial infection. Tuberculosis infection-associated DNA damage, toll-like receptor, and tumour necrosis factor-α activate the nuclear factor-*κ*B pathway and exert an anti-apoptotic effect on DNA-damaged cells [6]. Finally, epiregulin produced by tuberculosis-infected macrophages stimulates the proliferation of surrounding normal epithelial and stromal cells [6]. Similar up-regulated epiregulin expression has also been linked to activation of the Kras signalling pathway in colon cancer [6]. For lung cancer, pathological scarring due to ongoing inflammation might induce carcinogenesis [7]. Also, a relationship between tuberculosis and lung cancer epithelial-mesenchymal transition had been demonstrated [8]. Alterations of epithelial cell polarity induce carcinogenesis and are associated with tumour progression [22]. Although, the possibility of reverse causality should be taken into account as compromised immunity in cancer patients may increase the risk of latent tuberculosis activation or new tuberculosis infection, [23] reverse causality is unlikely to fully explain the long-term association between tuberculosis and cancer given the increase in cancer risk were observed even five to 20 years after the diagnosis of tuberculosis [24–26]. The underlying causal mechanism remains an active area of research, and more rigorously controlled preclinical studies are needed.

Strategies tailored to country’s context for tuberculosis control and elimination might have tremendous potential impact — not merely to reduce the burden of tuberculosis, but also to promote cancer prevention. As China and India account for 47% of tuberculosis-related cancer cases, the feasibility of these two countries to achieve the WHO target with existing interventions could further impact the global cancer burden [27]. In China, despite the high performance of the nationwide Directly Observed Treatment, Short-Course (DOTS) strategy, epidemic of drug-resistant tuberculosis remains as a major threat to tuberculosis control [28]. Future efforts should be focused on delivering rapid molecular tests for multidrug-resistant tuberculosis and appropriate treatments in peripheral and local health centres to achieve a further reduction in tuberculosis-related cancer incidence. In India, tackling of the key determinants of tuberculosis, such as undernutrition and cigarette smoking, and investment in health systems remain critical priorities to reduce the tuberculosis-related burden [29]. By raising awareness of the importance of the carcinogenic effect of tuberculosis, healthcare policymakers could ultimately lead to the proactive development of measures that positively affect the global cancer burden and therefore contribute to the global public good.

The results should be, however, interpreted with caution. First, meta-analyses of observational studies are susceptible to inherited confounding factors [30]. Smoking is the leading risk factor, [31] and a possible confounder for lung cancer [32, 33]. To overcome this issue, we restricted our PAF calculation of lung cancer using risk estimates adjusted for smoking. The meta-analysis for lung cancer was also re-run with never-smokers to eliminate the effects of smoking. Our risk estimate for never-smokers further suggested that tuberculosis has an independent association with lung cancer. In additional analyses, the adjustment of PAFs for smoking had only small differences in comparison with unadjusted PAFs for lung cancer. Although possible residual confounding cannot be excluded, we have tried to assess the effect of potential confounding using the best available data. Second, the set of studies was heterogeneous, and we could not fully analyse the source of heterogeneity as the individual patient-level data are not available. However, meta-regression showed that geographical region and study design explained 37% of study heterogeneity of lung cancer. Large prospective cohort studies are needed to further examine the association between tuberculosis and cancer at different sites. Also, future studies performing comprehensive subgroup analyses are warranted. Another limitation of this study is that we did not estimate PAFs separately for active tuberculosis and latent tuberculosis infection because tuberculosis in all form was assessed in 48 of 49 studies included.

## Conclusions

In summary, this study comprehensively explores carcinogenic risk and impact of tuberculosis on global cancer incidence. Our findings reveal that the efforts to achieve the SDG to end tuberculosis would potentially gain additional benefits on reduction of the burden of cancer, particularly in China and India. The present study provides insights into further research to resolve the underlying mechanisms, and to recognise the potential of individual countries to formulate efficient integrated strategies for the preventable burden of tuberculosis and cancers.

## Supplementary information

**Additional file 1.** PRISMA 2009 Checklist

**Additional file 2.** Supplementary Notes

## Data Availability

Not applicable.

## Abbreviations

CI: Confidence interval
GHDx: Global health data exchange
HR: Hazard ratio
NOS: Newcastle-Ottawa scale
OR: Odds ratio
PAFs: Population attributable fractions
RR: Relative risk
SDG: Sustainable development goal
SDI: Socio-demographic Index
