# Supplementary material for: Cancer incidence attributable to tuberculosis in 2015: global, regional, and national estimates
Source: BMC Cancer. 2020 May 12;20:412. doi: 10.1186/s12885-020-06891-5 (PMC7218646; doi:10.1186/s12885-020-06891-5)
Supplement: Supplementary file 2 — Additional file 2. Supplementary Notes [file 12885_2020_6891_MOESM2_ESM.docx]

**Supplementary**

**Cancer incidence attributable to tuberculosis in 2015:** **global, regional, and national estimates**

Chi Yan Leung^1,2#^, Hsi-Lan Huang^1,2#*^, Md. Mizanur Rahman^1^, Shuhei Nomura^1,3^, Sarah Krull Abe^1,4^, Eiko Saito^1,2^, Kenji Shibuya^1,5^

1. Department of Global Health Policy, Graduate School of Medicine, The University of Tokyo, Tokyo, Japan
2. Division of Cancer Statistics Integration, Center for Cancer Control and Information Services, National Cancer Center, Tokyo, Japan
3. Department of Health Policy and Management, School of Medicine, Keio University, Tokyo, Japan
4. Epidemiology and Prevention Group, Research Center for Cancer Prevention and Screening, National Cancer Center, Tokyo, Japan
5. University Institute for Population Health, King's College London, UK

# These authors contributed equally to this work

* Correspondence: Hsi-Lan Huang, Department of Global Health Policy, Graduate School of Medicine, The University of Tokyo, 7-3-1 Hongo, Bunkyo-ku, Tokyo 113-0033, Japan

Telephone: +81-3-5841-3688; Fax: +81-3-5841-3637; e-mail: hsilan0728@m.u-tokyo.ac.jp

**Supplementary**

**Supplementary Notes.**

*Supplementary tables*

**Table S1.** Search strategies on the PubMed

**Table S2.** Search strategies on the Web of Science

**Table S3.** Search strategies on the Ovid Embase

**Table S4.** Search strategies on the Cochrane library

**Table S5.** Search strategies on the CINAHL

**Table S6.** List of references with final exclusion reasons

**Table S7.** Study characteristics of included studies

**Table S8.** Newcastle-Ottawa Quality Assessment Scale for cohort studies

**Table S9.** Newcastle-Ottawa Quality Assessment Scale for case-control studies

**Table S10.** Subgroup and meta-regression for lung cancer

**Table S11.** Subgroup and meta-regression for non-Hodgkin’s lymphoma

**Table S12.** Subgroup and meta-regression for leukaemia

**Table S13.** Subgroup and meta-regression for multiple myeloma

**Table S14.** Pooled estimates of cancer risk, publication bias, and trim-and fill estimates; by cancer site

**Table S15.** Sensitivity analyses with summary estimates by with versus without dropping highly influential studies, by cancer type

**Table S16.** Proportion of cancer in 2015 attributable to tuberculosis in male, female and, both sexes; by country

**Table S17.** Tuberculosis attributable cancer cases by region, country, and SDI; both sexes

**Table S18.** Population attributable fraction (PAF) of tuberculosis on lung cancer in current and former smokers, never-smokers, and PAF adjusted and unadjusted for smoking status

**Table S19.** Population attributable fraction (PAF) of tuberculosis on lung cancer and leukaemia in base case analyses and PAF estimations with cohort studies exclusively

*Supplementary figures*

**Figure S1.** Study characteristics by year and the geographical region

**Figure S2.** Distribution of each component in quality assessment of each included study using the Newcastle-Ottawa Scale for quality assessment

**Figure S3.** Forest plot of lung cancer using the random-effects model

**Figure S4.** Forest plot of smoking-adjusted lung cancer using the random-effects model

**Figure S5.** Forest plot of lung cancer of never-smokers using the random-effects model

**Figure S6.** Forest plot of non-Hodgkin’s lymphoma, Hodgkin’s lymphoma, multiple myeloma, and leukaemia using the random-effects model

**Figure S7.** Forest plot of gastrointestinal cancer, hepatobiliary cancer, and pancreatic cancer using the random-effects model

**Figure S8.** Forest plot of malignant melanoma of skin, uterine cancer, ovarian cancer, prostate cancer, and kidney and bladder cancer using the random-effects model

**Figure S9.** Forest plot of head and neck cancer, breast cancer, central nervous system (CNS) cancer, and thyroid cancer using the random-effects model

**Figure S10.** Funnel plot of lung cancer, lung cancer (adjusted for smoking), and lung cancer of never-smokers

**References.**

**Supplementary Notes**

**Cancer outcomes**

In our study, 17 cancer outcomes assessed and their corresponding *International Classification of Diseases and Related Health Problem, 10^th^ revision* (ICD–10) codes were head and neck cancer (C00–14, C32), gastrointestinal cancer (C15–21), hepatobiliary cancer (C22–24), pancreatic cancer (C25), lung cancer (C33–34), malignant melanoma of skin (C43), breast cancer (C50), uterine cancer (C54), ovarian cancer (C56), prostate cancer (C61), kidney and bladder cancer (C64–67), CNS cancer (C70–72), thyroid cancer (C73), Hodgkin’s lymphoma (C81), Non-Hodgkin’s lymphoma (C82–85, C96), multiple myeloma (C88–90), and leukaemia (C91–95).^1^

**A standardized observation form for data extraction**

In data extraction, we used a standardized observation form to enter publication year, study population, country, study participant characteristics (age, sex, smoking status, HIV status), study sample size, and study design (case-control or cohort study). Outcomes were cancer incident cases of different cancer sites. We extracted summary estimate (relative risk, odds ratios, or hazard ratios with 95% confidence intervals) in each outcome measure directly or calculated it from results reported in the study.

**Newcastle-Ottawa Scale assessing risk of bias for case-control and cohort studies**

The Newcastle-Ottawa Scale was used to assess the methodological quality in the selection, comparability, and outcome of all included studies by two independent reviewers (CYL and HLH).^2^ Newcastle-Ottawa Scale is a risk of bias assessment tool for non-randomised studies. It was developed with a star-awarding-system to assess the methodological quality in three perspectives, including: the selection of study groups, the comparability of the groups, and the ascertainment of either the exposure or outcome of interest.

For included studies, “stars” were given to those items which met the pre-specified criteria during risk of bias assessment.^3^ Studies awarded three or four stars for selection, two for comparability, and two or three for ascertainment of the outcome were defined to have low risk of bias. Medium risk of bias defined as those studies that rewarded two stars for selection, and one or two stars for comparability, and two or three for ascertainment of the outcome. Studies with zero or one stars for selection, or zero stars for comparability, or zero or one stars for ascertainment of the outcome were defined to have high risk of bias.

**Definition of never-smokers**

In this study, never-smokers was defined as a person who has never smoked or has smoked <100 cigarettes in a lifetime, in accordance with the United States Department of Health and Human Services, Centers for Disease Control and Prevention definition.

**Grouping of countries and territories**

In our study, countries and territories were grouped into the following 11 geographical regions: Australasia; Central Europe, Eastern Europe, and Central Asia; High-income Asia Pacific; High-income North America; Latin America and Caribbean; North Africa and Middle East; South Asia; Southern Latin America; Southeast Asia, East Asia, and Oceania; Sub-Saharan Africa; and Western Europe.^4^ Countries and territories were grouped into five Socio-demographic Index (SDI) quintiles in 2015: High, High-middle, Middle, Low-middle, and Low.^4^ Countries and territories were also grouped into four World Bank income categories: High, middle-high, middle-low, and low.^5^ World Bank country classifications by income level: low-income economies are countries with a gross national income (GNI) per capita of $1025 or less; lower middle-income economies are countries with a GNI per capita between $1026 and $4035; upper middle-income economies are countries with a GNI per capita between $4036 and $12 475; and high-income economies are countries with a GNI per capita of $12 476 or higher.**Table S1. Search strategies on the PubMed**

| (#1 AND #2 AND #3 = 566 articles) |
| --- |
| #1 Exposure: Tuberculosis (266 381 articles)  tuberculosis [MeSH] OR tuberculosis [tw] OR TB [tw] OR latent tuberculosis OR latent TB [tw] OR tuberculosis infection OR inactive tuberculosis OR active tuberculosis OR active TB [tw] |
| #2 Outcome: Cancer (984 216 articles)  cancer [MeSH] OR lung cancer [MeSH] OR lung cancer [tw] OR neoplasm [MeSH] OR lung neoplasm [MeSH] OR carcinoma, non-small-cell lung [MeSH] OR carcinoma, non-small-cell lung [tw] OR non small cell lung cancer [tw] OR small cell lung cancer [tw] OR pulmonary malignancy [MeSH] OR pulmonary malignanc* [tw] OR lung adenocarcinoma [MeSH] OR lung adenocarcinoma [tw] OR lung squamous cell carcinoma [tw] OR lung large cell carcinoma [tw] OR head and neck neoplasm [MeSH] OR head and neck neoplasm [tw] OR esophageal neoplasm [MeSH] OR oesophageal cancer [tw] OR esophageal neoplasm [tw] OR colorectal neoplasm [MeSH] OR colorectal cancer [tw] OR liver neoplasm [MeSH] OR liver neoplasm [tw] OR liver cancer [tw] OR stomach neoplasm [MeSH] OR stomach neoplasm [tw] OR gastric cancer [tw] OR abdominal cancer [tw] OR abdominal carcinoma [tw] OR carcinomatosis [tw] OR head and neck malignan* [tw] OR oesophageal malignan* [tw] OR esophageal malignan* [tw] OR colorectal malignan* [tw] OR liver malignan* [tw] OR stomach malignan* [tw] OR gastric malignan* [tw] OR abdominal malignan* [tw] OR non-pulmonary malignan* [tw] OR hodgkin* disease [MeSH] OR hodgkin* disease OR lymphoma, non-hodgkin* [MeSH] OR lymphoma, non-hodgkin* OR leukemia [MeSH] OR leukemia [tw] OR haematopoietic cancer [MeSH] OR haematopoietic cancer* [tw] OR pyothorax* lymphoma [MeSH] OR pyothorax* lymphoma [tw] OR melanoma [tw] OR urothelial carcinoma [tw] OR urothelial cancer OR renal cell carcinoma [tw] OR melanoma [MeSH] OR urothelial carcinoma [MeSH] OR renal cell carcinoma [MeSH] OR breast cancer [MeSH] OR ovarian cancer [MeSH] OR uterine cancer [MeSH] OR cervical cancer [MeSH] OR endometrial cancer [MeSH] OR kidney and bladder cancer [MeSH] OR Prostate cancer [MeSH] OR breast cancer [tw] OR ovarian cancer [tw] OR uterine cancer [tw] OR cervical cancer [tw] OR endometrial cancer [tw] OR kidney and bladder cancer [tw] OR Prostate cancer [tw] |
| #3 Study design: Cohort and case-control (3 319 371 articles)  (Cohort studies [MeSH] OR Longitudinal Studies [MeSH] OR follow up studies [MeSH] OR prospective studies [MeSH] OR Case Control Studies [MeSH] OR Retrospective Studies [MeSH] OR Survival Analysis [MeSH] OR population-based stud* OR follow-up OR cohort [tw] OR longitudinal [tw] OR prospective [tw] OR retrospective [tw] OR Incidence Stud* [tw] OR Incidence Stud* [tw] OR Concurrent Stud* [tw] OR Follow Up [tw] OR (case [tw] AND control [tw])) NOT (meta-analysis [MeSH]) |

**Table S2. Search strategies on the Web of Science**

| (#1 AND #2 AND #3 = 497 articles) |
| --- |
| #1 Exposure: Tuberculosis (190 600 articles)  TI=(“tuberculosis” OR “TB” OR “latent tuberc*” OR “latent TB” OR “tuberc* infect*” OR “inactive tuberc*” OR “active tuberc*” OR “active TB”) OR TS=(“tuberculosis” OR “TB” OR “latent tuberc*” OR “latent TB” OR “tuberc* infect*” OR “inactive tuberc*” OR “active tuberc*” OR “active TB”) |
| #2 Outcome: Cancer (1 443 369 articles)  TI=(“cancer” OR “lung cancer” OR “lung neoplasm*” OR “carcinoma, non-small-cell lung” OR “non small cell lung cancer” OR “small cell lung cancer” OR “pulmonary malignan*” OR “lung adenocarcinoma” OR “lung squamous cell carcinoma” OR “lung large cell carcinoma” OR “head and neck neoplasm*” OR “esophageal neoplasm*” OR “oesophageal cancer” OR “colorectal neoplasm*” OR “colorectal cancer” OR “liver neoplasm*” OR “liver cancer” OR “stomach neoplasm*” OR “gastric cancer” OR “abdominal cancer” OR “abdominal carcinoma” OR “carcinomatosis” OR “head and neck malignan*” OR “oesophageal malignan*” OR “esophageal malignan*” OR colorectal malignan* OR liver malignan* OR stomach malignan* OR gastric malignan*” OR “abdominal malignan*” OR “non-pulmonary malignan*” OR “Hodgkin Disease*” OR “Lymphoma, Non-Hodgkin*” OR “Leukemia" OR “Haematopoietic cancer*” OR “Pyothorax* lymphoma” OR “melanoma” OR “urothelial carcinoma” OR “urothelial cancer” OR “renal cell carcinoma” OR “breast cancer” OR “ovarian cancer” OR “uterine cancer” OR “cervical cancer” OR “endometrial cancer” OR “kidney and bladder cancer” OR “prostate cancer”) OR TS=(“cancer” OR “lung cancer” OR “lung neoplasm*” OR “carcinoma, non-small-cell lung” OR “non small cell lung cancer” OR “small cell lung cancer” OR “pulmonary malignan*” OR “lung adenocarcinoma” OR “lung squamous cell carcinoma” OR “lung large cell carcinoma” OR “head and neck neoplasm*” OR “esophageal neoplasm*” OR “oesophageal cancer” OR “colorectal neoplasm*” OR “colorectal cancer” OR “liver neoplasm*” OR “liver cancer” OR “stomach neoplasm*” OR “gastric cancer” OR “abdominal cancer” OR “abdominal carcinoma” OR “carcinomatosis” OR “head and neck malignan*” OR “oesophageal malignan*” OR “esophageal malignan*” OR colorectal malignan* OR liver malignan* OR stomach malignan* OR gastric malignan*” OR “abdominal malignan*” OR “non-pulmonary malignan*” OR “Hodgkin Disease*” OR “Lymphoma, Non-Hodgkin*” OR “Leukemia" OR “Haematopoietic cancer*” OR “Pyothorax* lymphoma” OR “melanoma” OR “urothelial carcinoma” OR “urothelial cancer” OR “renal cell carcinoma” OR “breast cancer” OR “ovarian cancer” OR “uterine cancer” OR “cervical cancer” OR “endometrial cancer” OR “kidney and bladder cancer” OR “prostate cancer”) |
| #3 Study design: Cohort and case-control (3 659 797 articles)  TI=((Cohort stud* OR Longitudinal Stud* OR follow up stud* OR prospective stud* OR Case Control Stud* OR Retrospective Stud* OR Survival Analysis OR population-based stud* OR follow-up* OR cohort* OR longitudinal OR prospective OR retrospective OR Incidence Stud* OR Concurrent Stud* OR Follow Up OR (case AND control)) NOT (meta-analysis)) OR TS=((Cohort stud* OR Longitudinal Stud* OR follow up stud* OR prospective stud* OR Case Control Stud* OR Retrospective Stud* OR Survival Analysis OR population-based stud* OR follow-up* OR cohort* OR longitudinal OR prospective OR retrospective OR Incidence Stud* OR Concurrent Stud* OR Follow Up OR (case AND control)) NOT (meta-analysis)) |

**Table S3. Search strategies on the Ovid Embase**

| (#1 AND #2 AND #3 = 237 articles) |
| --- |
| 1# Exposure: Tuberculosis (239 328 articles)  tuberculosis OR TB OR latent tuberc* OR latent TB OR tuberc* infect* OR inactive tuberc* OR active tuberc* OR active TB |
| 2# Outcome: Cancer (227 207 articles)  cancer OR lung cancer OR lung neoplasm OR carcinoma, non-small-cell lung OR non small cell lung cancer OR small cell lung cancer OR pulmonary malignan* OR lung adenocarcinoma OR lung squamous cell carcinoma OR lung large cell carcinoma OR head and neck neoplasm* OR oesophageal cancer OR esophageal neoplasm* OR colorectal neoplasm* OR colorectal cancer OR liver neoplasm* OR liver cancer OR stomach neoplasm* OR stomach cancer OR gastric cancer OR abdominal cancer OR abdominal carcinoma OR carcinomatosis OR head and neck malignan* OR oesophageal malignan* OR esophageal malignan* OR colorectal malignan* OR liver malignan* OR stomach malignan* OR gastric malignan* OR abdominal malignan* OR non-pulmonary malignan* OR hodgkin* disease* OR hodgkin* disease* OR lymphoma, non-hodgkin* OR lymphoma, non-hodgkin* OR leukemia OR haematopoietic cancer* OR pyothorax* lymphoma OR pyothorax* lymphoma OR melanoma OR urothelial carcinoma OR urothelial cancer OR renal cell carcinoma OR breast cancer OR ovarian cancer OR uterine cancer OR cervical cancer OR endometrial cancer OR kidney and bladder cancer OR prostate cancer |
| #3 Study design: Cohort and case-control (4 227 238 articles)  (Cohort studies OR Longitudinal Studies OR follow up studies OR prospective studies OR Case Control Studies OR Retrospective Studies OR Survival Analysis OR population-based stud* OR follow-up* OR cohort* OR longitudinal OR prospective OR retrospective OR Incidence Stud* OR Incidence Stud* OR Concurrent Stud* OR Follow Up OR (case AND control)) NOT (meta-analysis) |

**Table S4. Search strategies on the Cochrane library**

| (#1 AND #2 AND #3 = 510 article) |
| --- |
| 1# Exposure: Tuberculosis (10 683 articles)  tuberculosis OR TB OR latent tuberc* OR latent TB OR tuberc* infect* OR inactive tuberc* OR active tuberc* OR active TB |
| 2# Outcome: Cancer (183 792 articles)  cancer OR lung cancer OR lung neoplasm OR carcinoma, non-small-cell lung OR non small cell lung cancer OR small cell lung cancer OR pulmonary malignan* OR lung adenocarcinoma OR lung squamous cell carcinoma OR lung large cell carcinoma OR head and neck neoplasm* OR oesophageal cancer OR esophageal neoplasm* OR colorectal neoplasm* OR colorectal cancer OR liver neoplasm* OR liver cancer OR stomach neoplasm* OR stomach cancer OR gastric cancer OR abdominal cancer OR abdominal carcinoma OR carcinomatosis OR head and neck malignan* OR oesophageal malignan* OR esophageal malignan* OR colorectal malignan* OR liver malignan* OR stomach malignan* OR gastric malignan* OR abdominal malignan* OR non-pulmonary malignan* OR hodgkin* disease* OR hodgkin* disease* OR lymphoma, non-hodgkin* OR lymphoma, non-hodgkin* OR leukemia OR haematopoietic cancer* OR pyothorax* lymphoma OR pyothorax* lymphoma OR melanoma OR urothelial carcinoma OR urothelial cancer OR renal cell carcinoma OR breast cancer OR ovarian cancer OR uterine cancer OR cervical cancer OR endometrial cancer OR kidney and bladder cancer OR prostate cancer |
| #3 Study design: Cohort and case-control (501 416 articles)  (Cohort studies OR Longitudinal Studies OR follow up studies OR prospective studies OR Case Control Studies OR Retrospective Studies OR Survival Analysis OR population-based stud* OR follow-up* OR cohort* OR longitudinal OR prospective OR retrospective OR Incidence Stud* OR Incidence Stud* OR Concurrent Stud* OR Follow Up OR (case AND control)) NOT (meta-analysis) |

**Table S5. Search strategies on the CINAHL**

| (S1 AND S2 AND S3 = 183 articles) |
| --- |
| S1 Exposure: Tuberculosis (21 673 articles)  TI (tuberculosis OR TB OR latent tuberc* OR latent TB OR tuberc* infect* OR inactive tuberc* OR active tuberc* OR active TB) OR AB (tuberculosis OR TB OR latent tuberc* OR latent TB OR tuberc* infect* OR inactive tuberc* OR active tuberc* OR active TB) |
| S2 Outcome: Cancer (359 746 articles)  TI (cancer OR lung cancer OR lung neoplasm* OR carcinoma, non-small-cell lung OR non small cell lung cancer OR small cell lung cancer OR pulmonary malignan* OR lung adenocarcinoma OR lung squamous cell carcinoma OR lung large cell carcinoma OR head and neck neoplasm* OR esophageal neoplasm* OR oesophageal cancer OR colorectal neoplasm* OR colorectal cancer OR liver neoplasm* OR liver cancer OR stomach neoplasm* OR gastric cancer OR abdominal cancer OR abdominal carcinoma OR carcinomatosis OR head and neck malignan* OR oesophageal malignan* OR esophageal malignan* OR colorectal malignan* OR liver malignan* OR stomach malignan* OR gastric malignan* OR abdominal malignan* OR non-pulmonary malignan* OR Hodgkin Disease* OR Lymphoma, Non-Hodgkin* OR Leukemia OR Haematopoietic cancer* OR Pyothorax* lymphoma OR melanoma OR urothelial carcinoma OR urothelial cancer OR renal cell carcinoma OR breast cancer OR ovarian cancer OR uterine cancer OR cervical cancer OR endometrial cancer OR kidney and bladder cancer OR prostate cancer) OR AB (cancer OR lung cancer OR lung neoplasm* OR carcinoma, non-small-cell lung OR non small cell lung cancer OR small cell lung cancer OR pulmonary malignan* OR lung adenocarcinoma OR lung squamous cell carcinoma OR lung large cell carcinoma OR head and neck neoplasm* OR esophageal neoplasm* OR oesophageal cancer OR colorectal neoplasm* OR colorectal cancer OR liver neoplasm* OR liver cancer OR stomach neoplasm* OR gastric cancer OR abdominal cancer OR abdominal carcinoma OR carcinomatosis OR head and neck malignan* OR oesophageal malignan* OR esophageal malignan* OR colorectal malignan* OR liver malignan* OR stomach malignan* OR gastric malignan* OR abdominal malignan* OR non-pulmonary malignan* OR Hodgkin Disease* OR Lymphoma, Non-Hodgkin* OR Leukemia OR Haematopoietic cancer* OR Pyothorax* lymphoma OR melanoma OR urothelial carcinoma OR urothelial cancer OR renal cell carcinoma OR breast cancer OR ovarian cancer OR uterine cancer OR cervical cancer OR endometrial cancer OR kidney and bladder cancer OR prostate cancer) |
| S3 Study design: Cohort and case-control (623 792 articles)  TI (Cohort stud* OR Longitudinal Stud* OR follow up stud* OR prospective stud* OR Case Control Stud* OR Retrospective Stud* OR Survival Analysis OR population-based stud* OR follow-up* OR cohort* OR longitudinal OR prospective OR retrospective OR Incidence Stud* OR Concurrent Stud* OR Follow Up OR (case AND control) NOT (meta-analysis)) OR AB (Cohort stud* OR Longitudinal Stud* OR follow up stud* OR prospective stud* OR Case Control Stud* OR Retrospective Stud* OR Survival Analysis OR population-based stud* OR follow-up* OR cohort* OR longitudinal OR prospective OR retrospective OR Incidence Stud* OR Concurrent Stud* OR Follow Up OR (case AND control) NOT (meta-analysis OR)) |

**Table S6. List of references with final exclusion reasons**

| Study | Reason of exclusion |
| --- | --- |
| Huang, J.Y., *et al*. The effects of pulmonary diseases on histologic types of lung cancer in both sexes: a population-based study in Taiwan. *BMC Cancer* 2015; 15:834. | Duplicated cohort |
| Jian, Z.H., *et al*. Post-Inhaled Corticosteroid Pulmonary Tuberculosis Increases Lung Cancer in Patients with Asthma. *PloS* One 2016; 11:e0159683. | Duplicated cohort |
| Lien, Y.C., *et al*. Urinary tuberculosis is associated with the development of urothelial carcinoma but not renal cell carcinoma: a nationwide cohort study in Taiwan. *British journal of cancer* 2013; 109:2933–2940. | Duplicated cohort |
| Su, V.Y., *et al*. Latent Tuberculosis Infection and the Risk of Subsequent Cancer. *Medicine* 2016; 95:e2352. | Duplicated cohort |
| Wu, C.Y., *et al*. Pulmonary tuberculosis increases the risk of lung cancer. *Cancer* 2011; 117:618–624. | Duplicated cohort |
| Wu, M.F., *et al*. Post-inhaled corticosteroid pulmonary tuberculosis and pneumonia increases lung cancer in patients with COPD. *BMC Cancer* 2016; 16:778. | Duplicated cohort |
| Jian, Z.H., *et al*. The coexistence of common pulmonary diseases on the histologic type of lung cancer in both genders in Taiwan: a STROBE-compliant article. *Medicine* 2014; 93:e127. | Duplicated cohort |
| Gao, Y.T., *et al*. Lung cancer among Chinese women. *International journal of cancer* 1987; 40:604–609. | Duplicated cohort |
| Kristinsson, S.Y., *et al*. Hodgkin lymphoma risk following infectious and chronic inflammatory diseases: a large population-based case–control study from Sweden. *International journal of hematology* 2015; 101:563–568. | Duplicated cohort |
| Kubı́k, A.K., Zatloukal, P., Tomášek, L. & Petruželka, L. Lung cancer risk among Czech women: a case–control study. *Preventive medicine* 2002; 34:436–444. | Duplicated cohort |
| Lai, S.W., *et al*. Antidiabetes drugs correlate with decreased risk of lung cancer: a population-based observation in Taiwan. *Clinical lung cancer* 2012; 13:143–148. | Duplicated cohort |
| Lo, Y.L., *et al*. Risk factors for primary lung cancer among never smokers by gender in a matched case–control study. *Cancer causes & control* 2013; 24:567–576. | Duplicated cohort |
| La Vecchia, C., Negri, E. & Franceschi, S. Medical history and the risk of non-Hodgkin's lymphomas. *Cancer epidemiology and prevention biomarkers* 1992; 1:533–536. | Duplicated cohort |
| Nakatsuka, S.I., *et al*. Pyothorax-associated lymphoma: a review of 106 cases. *Journal of clinical oncology* 2002; 20:4255–4260. | Review |
| Tobin, M.J. Chronic obstructive pulmonary disease, pollution, pulmonary vascular disease, transplantation, pleural disease, and lung cancer in AJRCCM 2003. *American journal of respiratory and critical care medicine* 2004; 169:301–313. | Review |
| Aoki, K. Excess incidence of lung cancer among pulmonary tuberculosis patients. *Japanese journal of clinical oncology* 1993; 23:205–220. | Review |
| Christensen, A.S., Roed, C., Andersen, P.H., Andersen, Å.B. & Obel, N. Long-term mortality in patients with pulmonary and extrapulmonary tuberculosis: a Danish nationwide cohort study. *Clinical epidemiology* 2014; 6:405. | Inappropriate outcome, report mortality |
| Engels, E.A., *et al*. Tuberculosis and subsequent risk of lung cancer in Xuanwei, China. *International journal of cancer* 2009; 124:1183–1187. | Inappropriate outcome, report mortality |
| Jian, Z.H., *et al*. Impact of coexisting pulmonary diseases on survival of patients with lung adenocarcinoma: a STROBE-compliant article. *Medicine* 2015; 94:e443. | Inappropriate outcome, report mortality |
| Jian, Z.H., *et al*. Pre-existing pulmonary diseases and survival in patients with stage-dependent lung adenocarcinoma: a STROBE-compliant article. *Medicine* 2016; 95. | Inappropriate outcome, report mortality |
| Leung, C.C., *et al*. Tuberculosis is associated with increased lung cancer mortality. *The international journal of tuberculosis and lung disease* 2013; 17:687–692. | Inappropriate outcome, report mortality |
| Mugusi, S.F., *et al*. Risk factors for mortality among HIV-positive patients with and without active tuberculosis in Dar es Salaam, Tanzania. *Antiviral therapy* 2012; 17:265. | Inappropriate outcome, report mortality |
| Tocque, K., Convrey, R.P., Bellis, M.A., Beeching, N.J. & Davies, P.D. Elevated mortality following diagnosis with a treatable disease: tuberculosis. *The international journal of tuberculosis and lung disease* 2005; 9:797–802. | Inappropriate outcome, report mortality |
| Tse, L.A., *et al*. Pulmonary tuberculosis and lung cancer mortality in a historical cohort of workers with asbestosis. *Public health* 2012; 126:1013–1016. | Inappropriate outcome, report mortality |
| Zhou, Y., *et al*. The presence of old pulmonary tuberculosis is an independent prognostic factor for squamous cell lung cancer survival. *Journal of cardiothoracic surgery* 2013; 8:123. | Inappropriate outcome, report mortality |
| Liu, C.J., *et al*. Risk and impact of tuberculosis in patients with chronic myeloid leukemia: A nationwide population‐based study in Taiwan. *International journal of cancer* 2015; 136:1881–1887. | Inappropriate outcome, report mortality |
| Fan, W.C., *et al*. Latent TB infection in newly diagnosed lung cancer patients–A multicenter prospective observational study. *Lung cancer* 2014; 85:472–478. | Inappropriate population |
| Han, X.Y., Tarrand, J.J., Infante, R., Jacobson, K.L. & Truong, M. Clinical significance and epidemiologic analyses of Mycobacterium avium and Mycobacterium intracellulare among patients without AIDS. *Journal of clinical microbiology* 2005; 43:4407–4412. | Inappropriate population |
| Luo, Y.H., *et al*. Association between tumor epidermal growth factor receptor mutation and pulmonary tuberculosis in patients with adenocarcinoma of the lungs. *Journal of thoracic oncology* 2012; 7:299–305. | Inappropriate population |
| Cocco, P., *et al*. Non-malignant respiratory diseases and lung cancer among Chinese workers exposed to silica. *Journal of occupational and environmental medicine* 2000; 42:639–644. | Inappropriate population |
| Seo, G.H., *et al*. Cancer-specific incidence rates of tuberculosis: A 5-year nationwide population-based study in a country with an intermediate tuberculosis burden. *Medicine* 2016; 95. | Inappropriate population |
| Stein, M.E., *et al*. Epidemic AIDS-related Kaposi's sarcoma in southern Africa: experience at the Johannesburg General Hospital (1980–1990). *Transactions of the royal society of tropical medicine and hygiene* 1994; 88:434–436. | Inappropriate population |
| Tamura, A., *et al*. Lung cancer in patients who had received thoracoplasty for pulmonary tuberculosis. *Japanese journal of clinical oncology* 1999; 29:541–545. | Inappropriate population |
| Shebl, F.M., Engels, E.A., Goedert, J.J. & Chaturvedi, A.K. Pulmonary infections and risk of lung cancer among persons with AIDS. *J acquir immune defic syndr* 2010; 55:375–379. | Inappropriate population |
| Choi, I.J., *et al*. Risk factors for TB in patients with early gastric cancer: is gastrectomy a significant risk factor for TB? *CHEST journal* 2015; 148:774–783. | Inappropriate population |
| Karakas, Z., *et al*. Pulmonary tuberculosis in children with Hodgkin's lymphoma. *The hematology journalI* 2003; 4:78–81. | Inappropriate population |
| Becker, N., Deeg, E. & Nieters, A. Population-based study of lymphoma in Germany: rationale, study design and first results. *Leukemia research* 2004; 28:713–724. | Inappropriate population |
| Silva, D.R., *et al*. Pulmonary tuberculosis and lung cancer: simultaneous and sequential occurrence. *Jornal brasileiro de pneumologia* 2013; 39:484–489. | Small sample size |
| Brincker, H. Interpretation of granulomatous lesions in malignancy. *Acta oncologica* 1992; 31:85–89. | Data cannot be extracted |
| Ernster, V.L. Female lung cancer. *Annual review of public health* 1996; 17:97–114. | Data cannot be extracted |
| Ger, L.P., Hsu, W.L., Chen, K.T. & Chen, C.J. Risk factors of lung cancer by histological category in Taiwan. *Anticancer research* 1993; 13:1491–1500. | Data cannot be extracted |
| Franceschi, S., *et al*. The epidemiology of non-Hodgkin's lymphoma in the north-east of Italy: a hospital-based case-control study. *Leukemia research* 1989; 13:465–472. | Data cannot be extracted |
| Liu, C.J., *et al*. Increased risk of tuberculosis in patients with acute myeloid leukemia in an endemic area: a nationwide population-based study. *EHA learning center* 2016; 134552. | Data cannot be extracted |

**Table S7. Study characteristics of included studies**

| Author | Country | Study year | Study type | Sex | Mean age | Outcome | Incident case | Confounding factors | Never-smokers |
| --- | --- | --- | --- | --- | --- | --- | --- | --- | --- |
| Everatt *et al*. (2017)^6^ | Lithuania | 1998–2012 | Cohort | Both | 47.1 | Extrapulmonary cancer, mouth and pharynx cancer, oesophageal cancer, stomach cancer, colon cancer, rectal cancer, liver cancer, pancreatic cancer, laryngeal cancer, melanoma, breast cancer, uterine cancer, ovarian cancer, prostate cancer, kidney cancer, bladder cancer, brain and CNS cancer, thyroid cancer, Hodgkin’s lymphoma, leukaemia | 2518 | Study controls for age, gender, smoking status, smoking frequency, education level, employment status, alcohol consumption, and TB site |  |
| Everatt *et al*.  (2016)^7^ | Lithuania | 1998–2012 | Cohort | Both | 47.1 | Lung cancer, small cell carcinoma of lung, adenocarcinoma of lung | 477 | Study controls for age, sex, smoking status, smoking frequency, education level, employment status, alcohol consumption, and TB site | Yes |
| Hong *et al.*  (2016)^8^ | Korea | 1997–2000 | Cohort | Both | 50.2 | Lung cancer | 12 819 | Study controls for age at enrolment, smoking status | Yes |
| Simonsen *et al*. (2014)^# 9^ | Denmark | 1977–2011 | Cohort | Both | 43.4 | Cancer, lung cancer, tongue, oral cavity, cancer of pharyngeal tonsil and cavity of pharynx, oesophageal cancer, stomach cancer, large intestinal and rectosigmoid cancer, rectal cancer, anal cancer, liver cancer, gallbladder and biliary tract cancer, pancreatic cancer, cancer of nasal cavity, middle ear and sinuses, cancer of larynx, malignant melanoma of skin, kaposi’s sarcoma, breast cancer, uterine cancer, ovarian cancer, prostate cancer, kidney cancer, urinary bladder cancer, cancer of membrane of brain and spinal meninx, brain cancer, thyroid cancer, Hodgkin’s lymphoma, non-Hodgkin’s lymphoma, lymphoid leukaemia, myeloid leukaemia | 2896 | Study controls for age; sex, comorbidity, TB patient's country of origin, and respiratory vs. non-respiratory TB |  |
| Kuo *et al.*  (2013)^10^ | Taiwan | 2000–2010 | Cohort | Both | 64.3 | Cancer, lung cancer, head and neck cancer, oesophageal cancer, stomach cancer, colon and rectal cancer, anal cancer, liver cancer, biliary tract cancer, pancreatic cancer, breast cancer, uterine cancer, ovarian cancer, prostate cancer, urinary bladder cancer, kidney cancer, CNS cancer, thyroid cancer, non-Hodgkin’s lymphoma, Hodgkin’s lymphoma, multiple myeloma, leukaemia, malignant melanoma of skin | 786 | Study controls for age; sex and follow-up period after tuberculosis diagnosis |  |
| Bae *et al.*  (2013)^11^ | Korea | 1992–1993 | Cohort | Male | 52.9 | Lung cancer | 93 | Study controls for age of entry, intake of coffee, tomatoes |  |
| Shiels *et al.*  (2011)^12^ | Finland | 1985–1988 | Cohort | Male | 57 | Lung cancer, squamous cell carcinoma of lung, small cell carcinoma of lung, adenocarcinoma of lung | 3102 | Study controls for age; baseline of tobacco use |  |
| Fan *et al.*  (2011)^13^ | China | 1992–1998 | Cohort | Both | 54.3 | Lung cancer, squamous cell carcinoma of lung, small cell carcinoma of lung, adenocarcinoma of lung | 502 | Study controls for age, sex, smoking status, smoking frequency, education level, occupational radon and arsenic exposure, and prior pulmonary disease |  |
| Yu *et al*.  (2011)^14^ | Taiwan | 1998–2000 | Cohort | Both | 45.4 | Lung cancer | 1684 | Study controls for age; sex |  |
| Littman *et al.*  (2004)^15^ | USA | 1985–2002 | Cohort | Both | 57.4 | Lung cancer, squamous cell carcinoma of lung, small cell carcinoma of lung, adenocarcinoma of lung | 1028 | Study controls for sex, exposure cohort, years smoked, years smoked squared, average number of cigarettes smoked per day, average number of cigarettes smoked per day squared, all other lung disease, smoking status |  |
| Askling and Ekbom  (2001)^16^ | Sweden | 1939–1996 | Cohort | Both | n/a | Cancer, non-Hodgkin’s lymphoma, Hodgkin’s lymphoma | 1073 | Study controls for age; sex and calendar period |  |
| Yang *et al.*  (2015)^17^ a | China | 2002–2011 | Case-control | Both | 54.3 | Lung cancer | 1056 | Study controls for age; sex, BMI, educational experience |  |
| Yang *et al.*  (2015)^17^ b | China | 2002–2011 | Case-control | Both | 54.3 | Lung cancer | 503 | Study controls for age; sex, BMI, educational experience |  |
| HosgoodIII *et al.*  (2013)^18^ | China | 1985–1990 | Case-control | Both | n/a | Lung cancer | 498 | Study controls for sex, fuel type used for household heating and cooking, educational status, lung cancer in first-degree relatives, cumulative lifetime hours spent at home, ever worked as coal miner, and tobacco use |  |
| Fan *et al.*  (2012)^19^ | China | 2003–2007 | Case-control | Both | 58.3 | Diffuse large B-cell lymphoma | 147 | Study controls for age; sex, medical history, lifestyle, residential, and environmental exposure |  |
| Park *et al*.  (2010)^20^ | Korea | 1997 | Case-control | Both | 56.7 | Lung cancer, squamous cell carcinoma of lung, small cell carcinoma of lung, adenocarcinoma of lung | 4210 | Study controls for age; smoking status | Yes |
| Koshiol *et al.*  (2010)^21^ | Italy | 2002–2005 | Case-control | Both | 66.5 | Lung cancer | 1890 | Study controls for age; sex, region, and chronic bronchitis for never smokers as well as pack–years and smoking intensity for smokers and overall | Yes |
| Brenner *et al.*  (2010)^22^ | Canada | 1997–2002 | Case-control | Both | 57.2 | Lung cancer | 445 | Study controls for age; smoking; sex; education, and ethnicity | Yes |
| Liang *et al.*  (2009)^23^ | China | 2004–2007 | Case-control | Female | 54.62 | Lung cancer, squamous cell carcinoma of lung, small cell carcinoma of lung, adenocarcinoma of lung | 226 | Study controls for age; marital status, years of schooling, ethnicity, 5 years ago BMI, passive smoking exposure, coal use, exposure to coal smoke and cooking fumes, chronic bronchitis, emphysema, asthma, bronchiectasis, other previous non-malignant respiratory conditions | Yes |
| Wang *et al.*  (2009)^24^ | Hong Kong | 2002–2004 | Case-control | Female | 62.9 | Lung cancer | 212 | Study controls for age; employment, total cooking dish-year, and intakes of yellow/orange vegetables, dark green vegetables, and multivitamins |  |
| Galeone *et al.*  (2008)^25^ | China | 1987–1990 | Case-control | Both | n/a | Lung cancer | 218 | Study controls for age; area of residence; smoking; occupation; income; family history of cancer; occupational exposure to recognized lung carcinogens |  |
| Ramanakumar *et al.*  (2006)^26^ a | Canada | 1979–1986 | Case-control | Both | 62.1 | Lung cancer, squamous cell carcinoma of lung, adenocarcinoma of lung | 1205 | Study controls for age; ethnicity, type of respondent, years of schooling, family income, and smoking |  |
| Ramanakumar *et al.*  (2006)^26^ b | Canada | 1979–1986 | Case-control | Male | 58.6 | Lung cancer, squamous cell carcinoma of lung, adenocarcinoma of lung | 755 | Study controls for age; ethnicity, type of respondent, years of schooling, family income, and smoking |  |
| Zatloukal *et al.*  (2003)^27^ | Czech Republic | 1998–2002 | Case-control | Female | 69 | Adenocarcinoma of lung | 366 | Study controls for age; residence; education; pack-year of smoking |  |
| Chan-Yeung *et al.*  (2003)^28^ | Hong Kong | 1999–2001 | Case-control | Both | 57.5 | Lung cancer | 331 | Study controls for smoking amount and duration |  |
| Kreuzer *et al.*  (2002)^29^ | Germany | 1991–1996 | Case-control | Female | 62 | Lung cancer | 234 | Study controls for age; region | Yes |
| Brenner *et al.*  (2001)^30^ | China | 1994–1998 | Case-control | Both | 55.7 | Lung cancer | 886 | Study controls for age; sex, prefecture, active smoking, passive smoking, radon exposure, socioeconomic status, coal combustion, and fume |  |
| Lee *et al.*  (2001)^31^ | Taiwan | 1993–1999 | Case-control | Both | 62.3 | Lung cancer | 527 | Study controls for smoking; residential area; education, and socioeconomic status |  |
| Kreuzer *et al.*  (2001)^32^ | Germany | 1990–1996 | Case-control | Male | 58.9 | Lung cancer | 58 | Study controls for age; area | Yes |
| Brownson *et al.*  (2000)^33^ | USA | 1993–1994 | Case-control | Female | 66.2 | Lung cancer | 676 | Study controls for pack-year of smoking |  |
| Osann *et al.*  (2000)^34^ | USA | 1990–1993 | Case-control | Female | 61.7 | Small cell carcinoma of lung | 98 | Study controls for age; education; smoking |  |
| Vineis *et al.*  (2000)^35^ | Italy | 1990–1993 | Case-control | Both | 56.1 | Haematolymphopoietic cancer, non-Hodgkin’s lymphoma, Hodgkin’s lymphoma, multiple myeloma, lymphoid leukaemia, myeloid leukaemia | 2669 | Study controls for age; sex |  |
| *Tavani et al.*  (2000)^36^ | Italy | 1983–1992 | Case-control | Both | 52.8 | Non-Hodgkin’s lymphoma, Hodgkin’s lymphoma | 587 | Study controls for age; sex, and education |  |
| Kolmel *et al.*  (1999)^37^ | Germany, Estonia, Austria, Bulgaria, Israel, Italy, France | 1994–1997 | Case-control | Both | 43.4 | Cutaneous malignant melanoma | 603 | Study controls for age; sex, centre, socioeconomic status and melanoma risk factors |  |
| Ko *et al.*  (1997)^38^ | Taiwan | 1992–1993 | Case- control | Female | 58.1 | Lung cancer | 105 | Study controls for socioeconomic status, residential area, and education | Yes |
| Schwartz *et al.*  (1996)^39^ | USA | 1984–1987 | Case-control | Both | 58.9 | Lung cancer | 257 | Study controls for age; race; sex | Yes |
| Wu *et al.*  (1995)^40^ | USA | 1985–1990 | Case-control | Female | n/a | Lung cancer | 653 | Study controls for age; area; ethnicity; education, and exposure to environmental tobacco smoking | Yes |
| Zheng *et al.* (1993)^41^ | China | 1987–1989 | Case-control | Both | 47.2 | Acute lymphocytic leukaemia, Acute nonlymphocytic leukaemia, Chronic myelocytic leukaemia | 486 | Study controls for age; sex, income, and occupation |  |
| Ger *et al.*  (1993)^42^ | Taiwan | 1990–1991 | Case-control | Both | 60.1 | Lung cancer, squamous cell carcinoma of lung, small cell carcinoma of lung, adenocarcinoma of lung | 143 | Study controls for multiple variables but not listed detail in the article |  |
| Alavanja *et al.*  (1992)^43^ | USA | 1986–1991 | Case-control | Female | 71.5 | Lung cancer | 618 | Study controls for age; smoking | Yes |
| Doody *et al.*  (1992)^44^ | USA | 1956–1982 | Case-control | Both | 47 | Leukaemia, acute myelocytic leukaemia, chronic myelocytic leukaemia, unclassified leukaemia, chronic lymphocytic leukaemia, multiple myeloma, non-Hodgkin’s lymphoma | 574 | Study controls for age; sex, region, number of years as a member in the program, and calendar year in which membership began |  |
| Rosenblatt *et al.*  (1991)^45^ | USA | 1977–1981 | Case-control | Both | 63.8 | Chronic lymphocytic leukaemia | 430 | Study controls for age; race, sex, study area, and educational level |  |
| Gramenzi *et al*. (1991)^46^ | Italy | 1983–1989 | Case-control | Both | 59.8 | Multiple myeloma | 117 | Study controls for age; sex |  |
| Wu *et al.*  (1990)^47^ | China | 1985–1987 | Case-control | Female | 61.7 | Lung cancer | 965 | Study controls for age; education; personal smoking; study area |  |
| Wu *et al.*  (1988)^48^ | USA | 1983–1986 | Case-control | Female | 62 | Adenocarcinoma of lung | 336 | Study controls for pack-year of smoking; years since smoking stopped; depth of inhalation |  |
| Zheng *et al.*  (1987)^49^ | China | 1984–1986 | Case-control | Both | 57.85 | Lung cancer, squamous cell carcinoma of lung, adenocarcinoma of lung | 1405 | Study controls for age; sex, education, and smoking status |  |
| Samet *et al.*  (1986)^50^ | USA | 1980–1982 | Case-control | Both | n/a | Lung cancer | 518 | Study controls for age; sex; ethnicity; smoking |  |
| *Bernard et al*.  (1984)^51^ | UK | 1979–1981 | Case-control | Both | n/a | Non-Hodgkin’s lymphoma, lymphoid Leukaemia | 285 | Study controls for age; sex, and area of residence |  |
| Hinds *et al.*  (1982)^52^ | USA | 1968–1978 | Case-control | Female | n/a | Lung cancer | 210 | Unadjusted | Yes |

Note: ^#^This study reported only active tuberculosis. TB: tuberculosis, CNS: central nervous system.

The study characteristics by year and geographical region are presented in Figure S1. The studies were published between 1982 and 2017, with two-thirds (33/49) published after 2000. The incident cases in each study ranged from 44 to 12 819, and studies with larger sample sizes were clustered in recent decades. 18 in studies were conducted in Southeast Asia, East Asia, and Oceania; 14 studies in High-income North America; 11 in Western Europe; three in High-income Asia Pacific; and three in Central Europe, Eastern Europe, and Central Asia.

**Figure S1. Study characteristics by year and the geographical region**

**Table S8. Newcastle-Ottawa Quality Assessment Scale for cohort studies**

| Author | Year | Selection |  |  |  | Comparability | Outcome |  |  | Total score | Risk of bias |
| --- | --- | --- | --- | --- | --- | --- | --- | --- | --- | --- | --- |
|  |  | **Representativeness of the exposed cohort** | **Selection of the non-exposed cohort** | **Ascertainment of exposure** | **Demonstration that outcome of interest was not present at start of study** | **Comparability of cohorts on the basis of the design or analysis** | **Assessment of outcome** | **Was follow-up long enough for outcomes to occur** | **Adequacy of follow up of cohorts** |  |  |
| Everatt *et al*. ^6^ | 2017 | * | * | * | * | ** | * | * | * | 9 | Low |
| Everatt *et al*. ^7^ | 2016 | * | * | * | * | ** | * | * | * | 9 | Low |
| Hong *et al*. ^8^ | 2016 | * | * | * |  | ** | * | * | * | 8 | Low |
| Simonsen *et al*. ^9^ | 2014 | * | * | * | * | ** | * | * | * | 9 | Low |
| Kuo *et al*. ^10^ | 2013 | * | * | * | * | ** | * | * | * | 9 | Low |
| Bae *et al*. ^11^ | 2013 | * | * | * | * | ** | * | * | * | 9 | Low |
| Shiels *et al*. ^12^ | 2011 | * | * | * | * | ** | * | * | * | 9 | Low |
| Fan *et al*. ^13^ | 2011 |  |  | * |  | ** | * | * |  | 5 | High |
| Yu *et al*. ^14^ | 2011 | * | * | * |  | ** | * | * | * | 8 | Low |
| Littman *et al*. ^15^ | 2004 | * | * | * | * | * | * | * | * | 8 | Low |
| Askling and Ekbom ^16^ | 2001 | * | * | * |  | ** | * | * | * | 8 | Low |

Note: Studies awarded three or four stars for selection, two for comparability, and two or three for ascertainment of the outcome were defined to have low risk of bias. Medium risk of bias defined as those studies that rewarded two stars for selection, and one or two stars for comparability, and two or three for ascertainment of the outcome. Studies with zero or one stars for selection, or zero stars for comparability, or zero or one stars for ascertainment of the outcome were defined to have high risk of bias.

**Table S9. Newcastle-Ottawa Quality Assessment Scale for case-control studies**

| Author | Year | Selection |  |  |  | Comparability | Exposure |  |  | Total score | Risk of bias |
| --- | --- | --- | --- | --- | --- | --- | --- | --- | --- | --- | --- |
|  |  | **Adequate case definition** | **Cases representativeness** | **Controls selection** | **Controls definition** | **Comparability** | **Ascertainment of exposure** | **Same method of ascertainment for cases and controls** | **Non-response rate** |  |  |
| Yang *et al.* ^17^ | 2015 | * | * | * | * | ** |  | * |  | 7 | High |
| HosgoodIII *et al.* ^18^ | 2013 | * | * | * |  | * |  | * | * | 6 | Low |
| Fan *et al.* ^19^ | 2012 | * | * |  | * | ** | * | * |  | 7 | Low |
| Park *et al.* ^20^ | 2010 | * | * | * | * | ** |  | * |  | 7 | High |
| Koshiol *et al.* ^21^ | 2010 | * | * | * |  | ** |  | * | * | 7 | Low |
| Brenner *et al.* ^22^ | 2010 | * | * | * | * | ** |  | * | * | 8 | Low |
| Liang *et al.* ^23^ | 2009 | * | * | * | * | ** |  | * | * | 8 | Low |
| Wang *et al*. ^24^ | 2009 | * | * | * | * | ** | * | * |  | 8 | Low |
| Galeone *et al.* ^25^ | 2008 | * | * | * |  | ** |  | * |  | 6 | High |
| Ramanakumar *et al.* ^26^ | 2006 | * | * | * | * | ** |  | * | * | 8 | Low |
| Zatloukal *et al*. ^27^ | 2003 | * | * | * |  | ** |  | * | * | 7 | Low |
| Chan-Yeung *et al.* ^28^ | 2003 | * | * |  | * | * |  | * |  | 5 | High |
| Kreuzer *et al.* ^29^ | 2002 | * | * | * | * | ** |  | * |  | 7 | High |
| Brenner *et al.* ^30^ | 2001 | * | * | * | * | ** |  | * | * | 8 | Low |
| Lee *et al.* ^31^ | 2001 | * | * |  | * | * |  | * | * | 6 | Low |
| Kreuzer *et al.* ^32^ | 2001 | * | * | * |  | ** |  | * |  | 6 | High |
| Brownson *et al.* ^33^ | 2000 | * | * | * | * | * |  | * | * | 7 | Low |
| Osann *et al.* ^34^ | 2000 | * | * | * | * | ** |  | * | * | 8 | Low |
| Vineis *et al.* ^35^ | 2000 | * | * | * |  | ** | * | * |  | 7 | Low |
| Tavani *et al.* ^36^ | 2000 | * | * |  |  | ** |  | * | * | 6 | Medium |
| Kolmel *et al.* ^37^ | 1999 | * | * | * | * | ** |  | * | * | 8 | Low |
| Ko *et al.* ^38^ | 1997 | * | * |  |  | * | * | * | * | 6 | Medium |
| Schwartz *et al*. ^39^ | 1996 |  | * | * | * | ** |  | * | * | 7 | Low |
| Wu *et al.* ^40^ | 1995 | * | * | * |  | ** |  | * | * | 7 | Low |
| Zheng *et al.* ^41^ | 1993 | * | * | * |  | ** | * | * |  | 7 | Low |
| Ger *et al.* ^42^ | 1993 | * | * | * |  | * |  | * | * | 6 | Low |
| Alavanja *et al.* ^43^ | 1992 | * | * | * | * | ** |  | * | * | 8 | Low |
| Doody *et al.* ^44^ | 1992 | * | * | * |  | ** |  | * |  | 6 | High |
| Rosenblatt *et al.* ^45^ | 1991 |  | * | * |  | ** |  | * |  | 5 | High |
| Gramenzi *et al.* ^46^ | 1991 | * | * |  | * | ** |  | * | * | 6 | Low |
| Wu *et al*. ^47^ | 1990 | * | * | * |  | ** |  | * | * | 7 | Low |
| Wu *et al*. ^48^ | 1988 | * | * | * |  | * |  | * | * | 6 | Low |
| Zheng *et al.* ^49^ | 1987 | * | * | * |  | ** |  | * | * | 7 | Low |
| Samet *et al*. ^50^ | 1986 | * | * | * |  | ** |  | * | * | 7 | Low |
| Bernard *et al.* ^51^ | 1984 | * | * |  | * | ** |  | * | * | 7 | Low |
| Hinds *et al*. ^52^ | 1982 | * | * | * |  |  |  | * |  | 4 | High |

Note: Studies awarded three or four stars for selection, two for comparability, and two or three for ascertainment of the outcome were defined to have low risk of bias. Medium risk of bias defined as those studies that rewarded two stars for selection, and one or two stars for comparability, and two or three for ascertainment of the outcome. Studies with zero or one stars for selection, or zero stars for comparability, or zero or one stars for ascertainment of the outcome were defined to have high risk of bias.

**Figure S2. Distribution of each component in quality assessment of each included study using the Newcastle-Ottawa Scale for quality assessment**

Note: Three components include selection, with a maximum 4 stars; comparability, with maximum 2 stars; and outcome, with maximum 3 stars.

**Figure S3. Forest plot of lung cancer using the random-effects model**


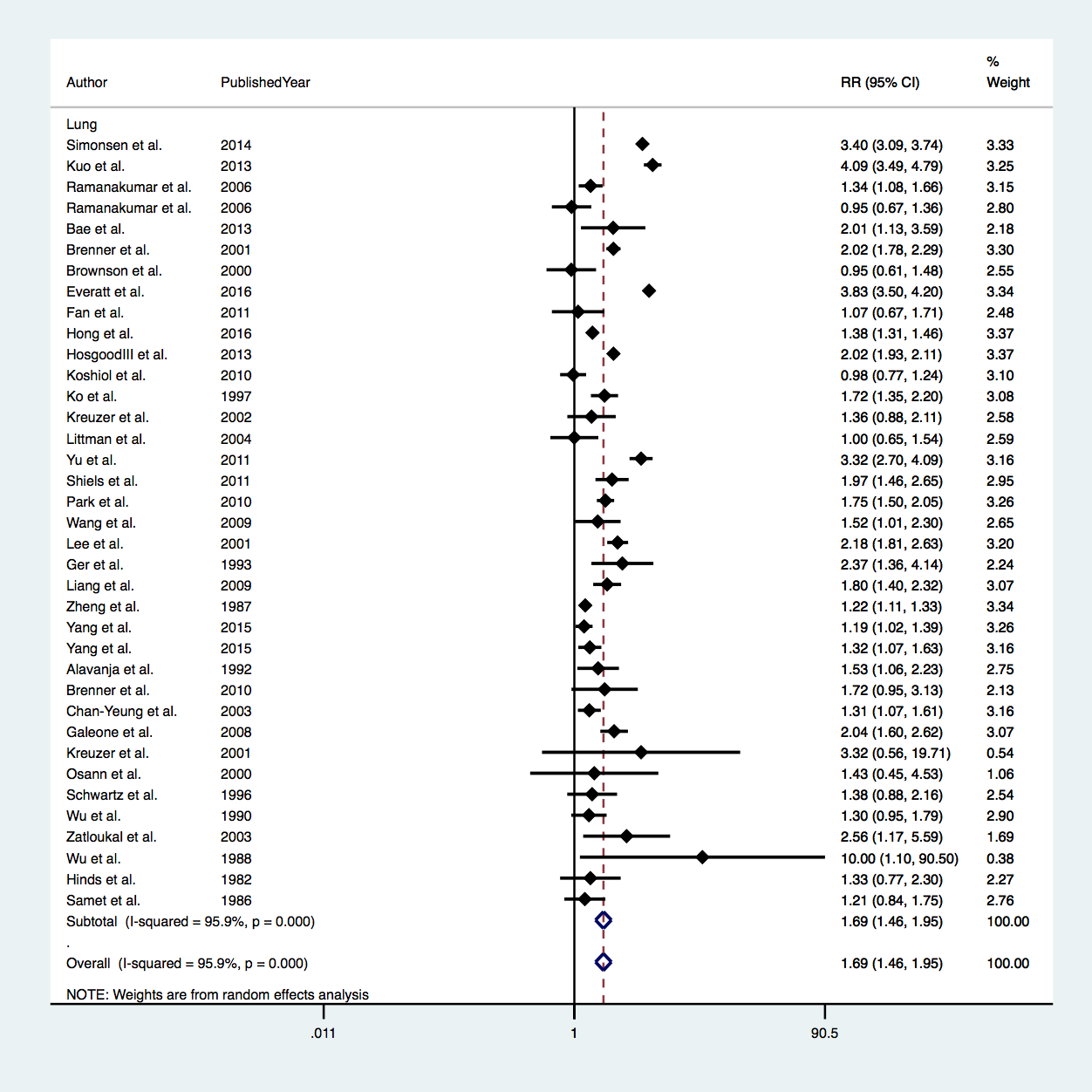


Note: RR: relative risk, CI: confidence interval, p: p-value.

**Figure S4. Forest plot of smoking-adjusted lung cancer using the random-effects model**


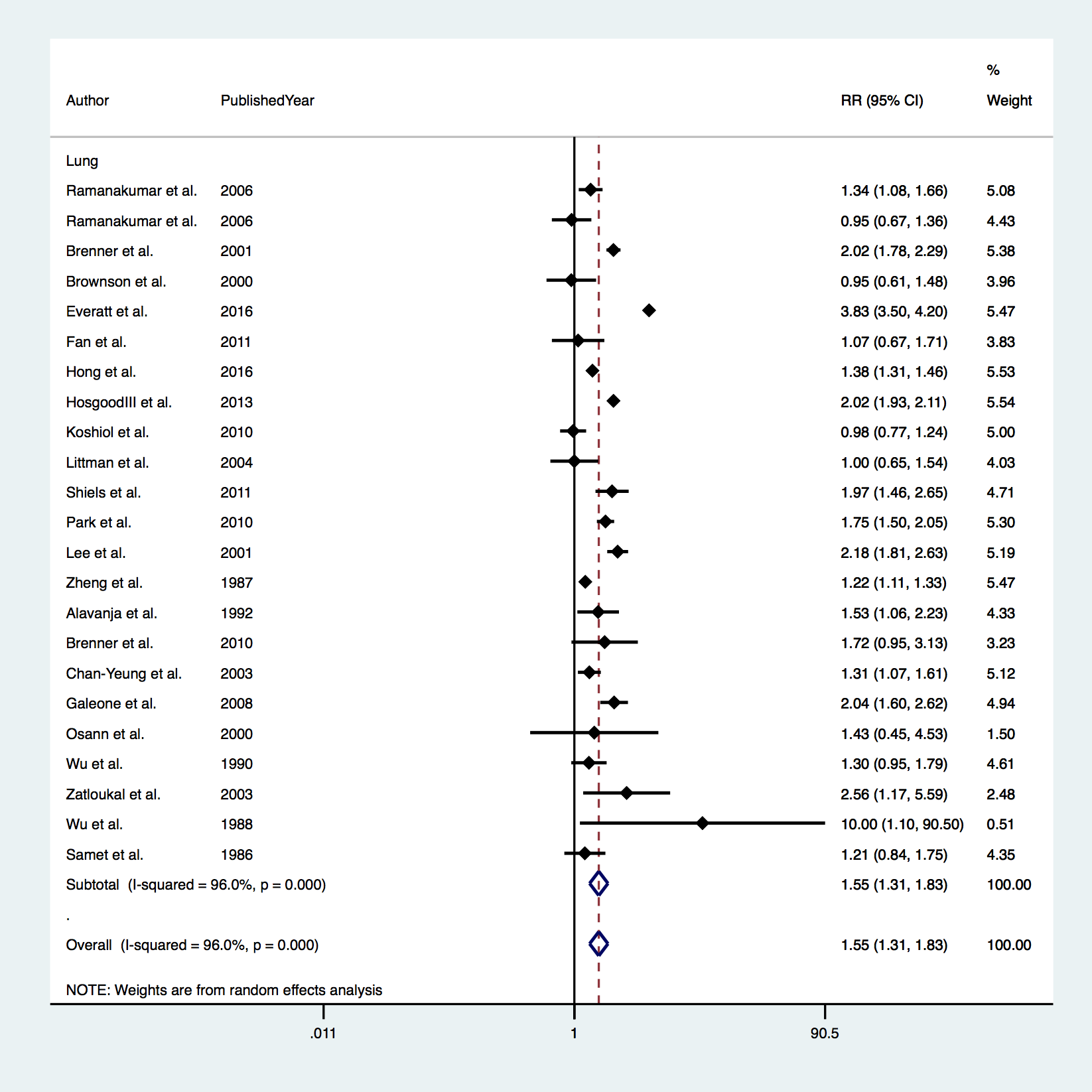


Note: RR: relative risk, CI: confidence interval, p: p-value.

**Figure S5. Forest plot of lung cancer of never-smokers using the random-effects model**


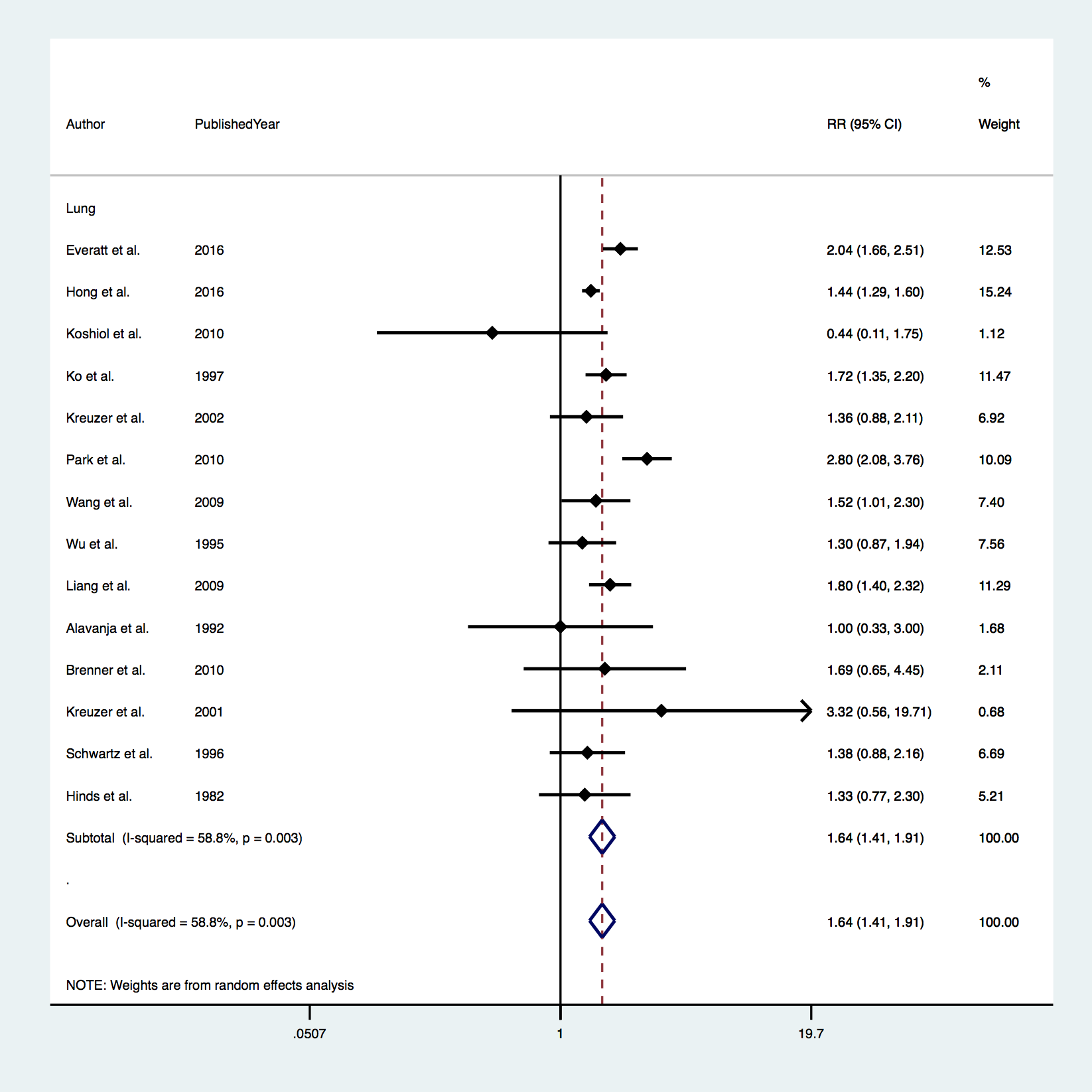


Note: RR: relative risk, CI: confidence interval, p: p-value.

**Figure S6. Forest plot of non-Hodgkin’s lymphoma, Hodgkin’s lymphoma, multiple myeloma, and leukaemia using the random-effects model**


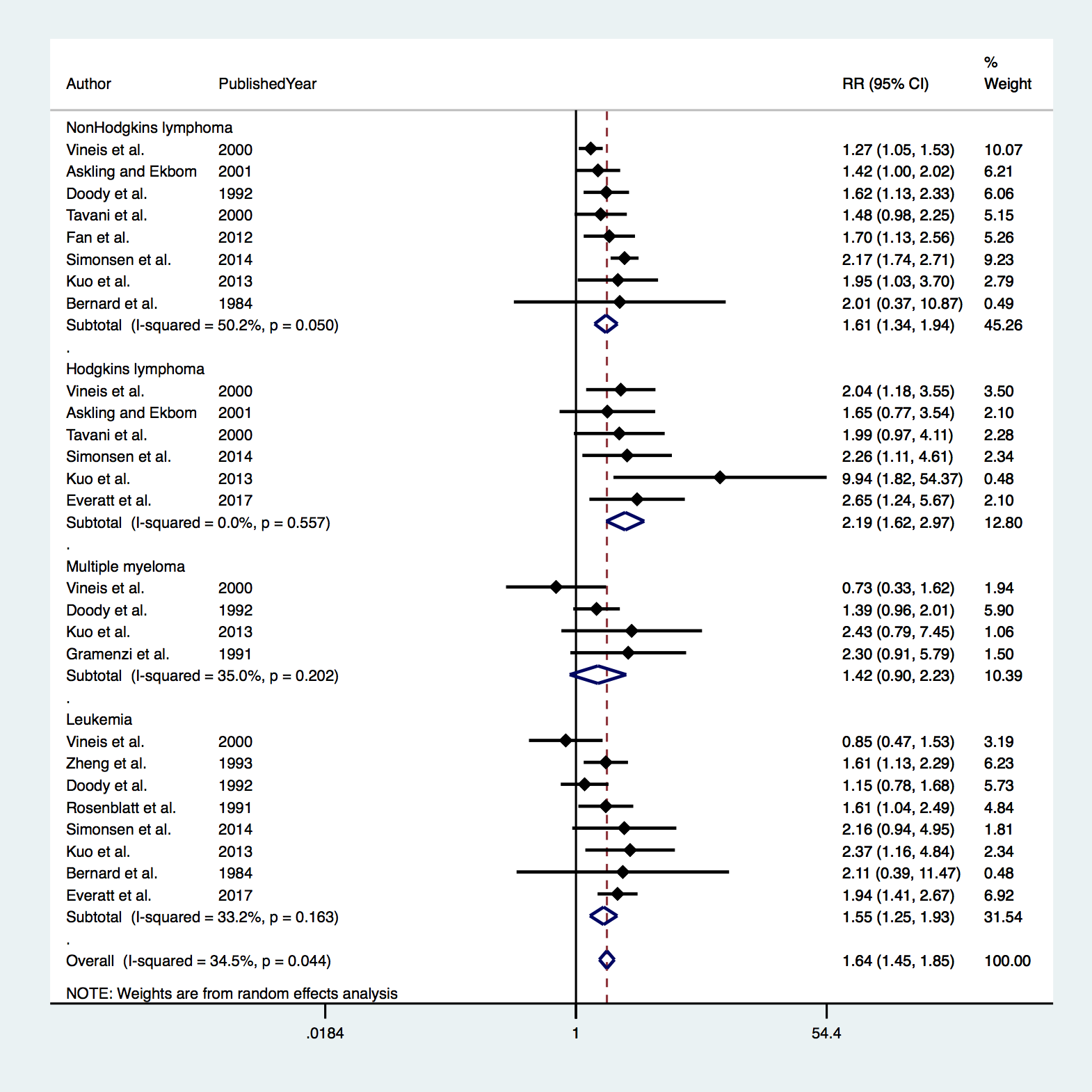


Note: RR: relative risk, CI: confidence interval, p: p-value.

**Figure S7. Forest plot of gastrointestinal cancer, hepatobiliary cancer, and pancreatic cancer using the random-effects model**


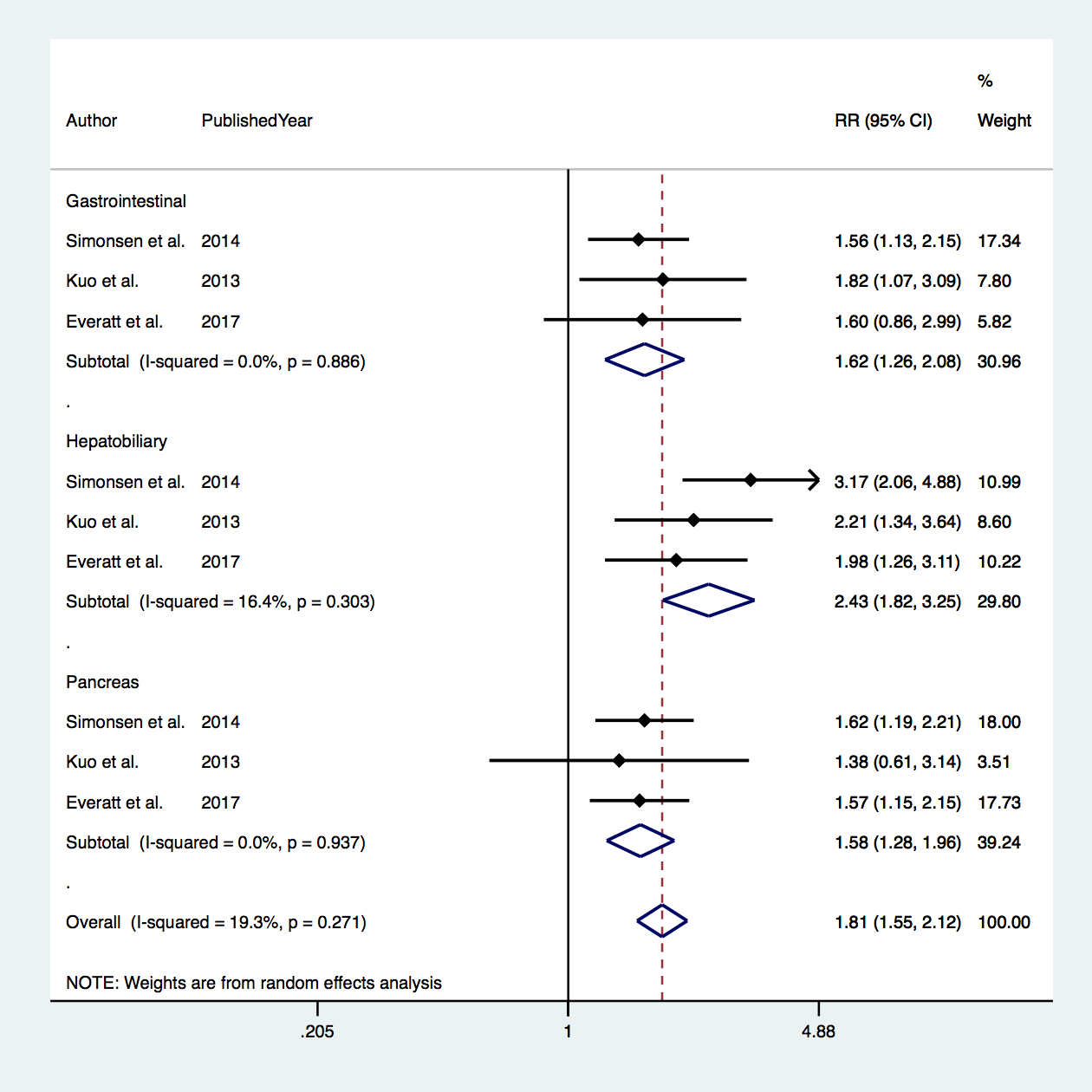


Note: RR: relative risk, CI: confidence interval, p: p-value.

**Figure S8. Forest plot of malignant melanoma of skin, uterine cancer, ovarian cancer, prostate cancer, and kidney and bladder cancer using the random-effects model**


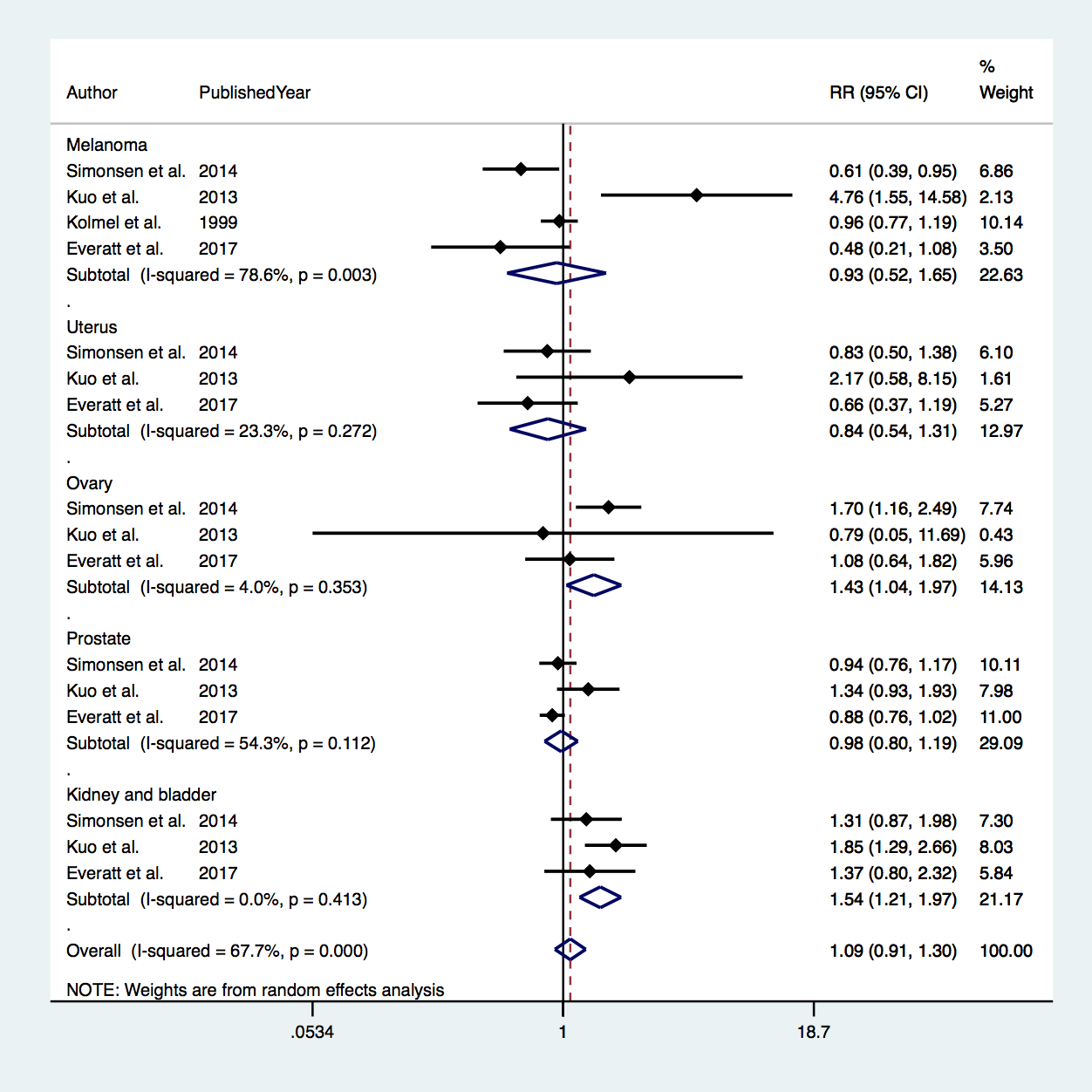


Note: RR: relative risk, CI: confidence interval, p: p-value.

**Figure S9. Forest plot of head and neck cancer, breast cancer, central nervous system (CNS) cancer, and thyroid cancer using the random-effects model**


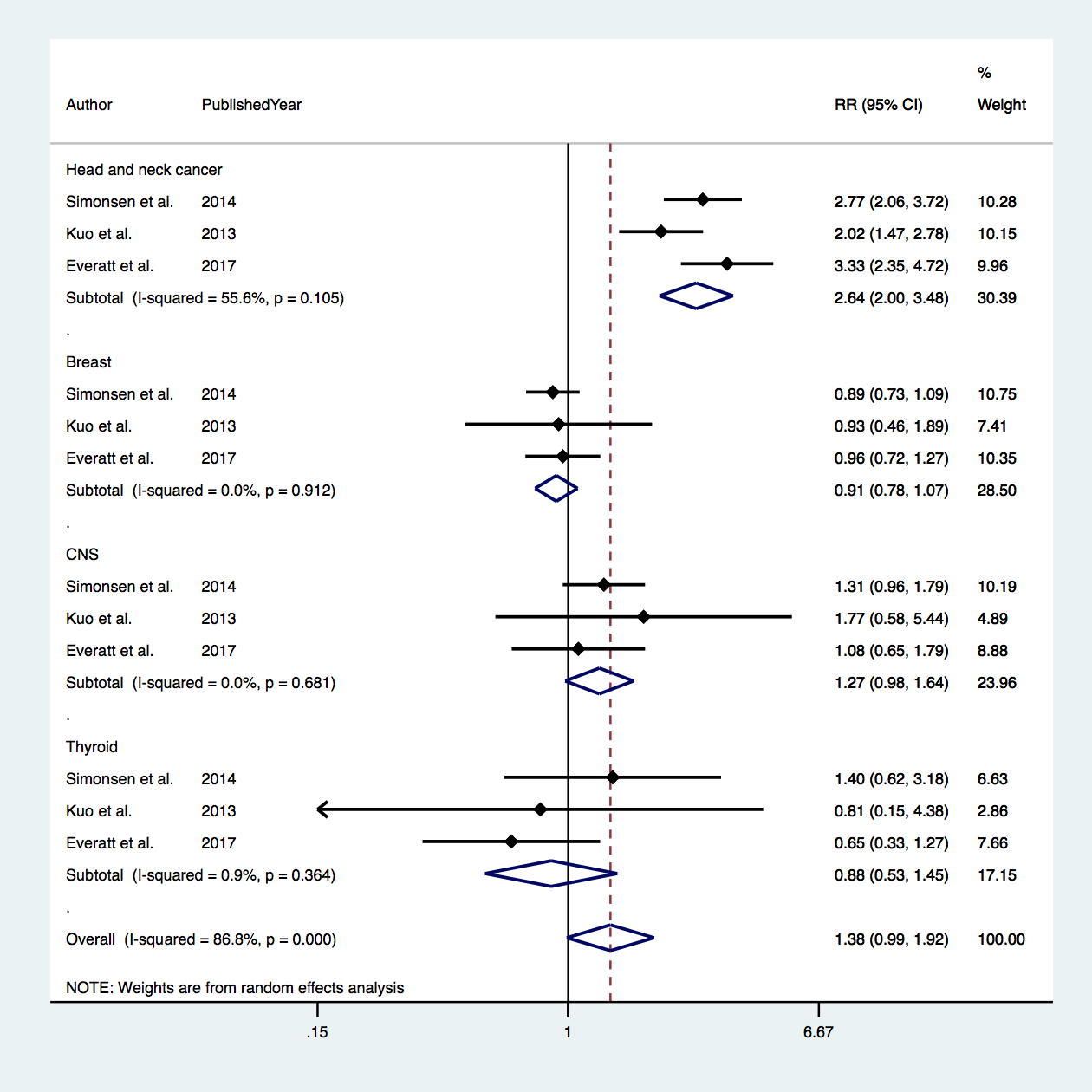


Note: RR: relative risk, CI: confidence interval, CNS: central nervous system, p: p-value.

**Table S10. Subgroup and meta-regression for lung cancer**

|  |  |  |  | P-value | |
| --- | --- | --- | --- | --- | --- |
| Characteristic | **Studies, n** | **Pooled RR**  **(95% CI)** | ***I^2^,* %** | **Heterogeneity** | **Meta-regression** |
| Lung cancer | 37 |  |  |  |  |
| Study design | 37 |  |  |  | 0.0227 |
| Case-control | 28 | 1.51 (1.34, 1.70) | 87.6 | 0 |  |
| Cohort | 9 | 2.21 (1.48, 3.30) | 98.7 | 0 |  |
| World Bank Country-income | 37 |  |  |  | 0.4142 |
| High | 28 | 1.76 (1.42, 2.17) | 96.2 | 0 |  |
| Upper middle | 9 | 1.53 (1.25, 1.87) | 94.6 | 0 |  |
| Region | 37 |  |  |  | 0.0305 |
| Central Europe, Eastern Europe, and Central Asia | 2 | 3.80 (3.43, 4.21) | 0.7 | 0.315 |  |
| High-income Asia Pacific | 3 | 1.58 (1.28, 1.98) | 78.2 | 0.010 |  |
| High-income North America | 11 | 1.25 (1.09, 1.43) | 10.8 | 0.342 |  |
| Southeast Asia, East Asia, and Oceania | 16 | 1.79 (1.50, 2.13) | 94.9 | 0 |  |
| Western Europe | 5 | 1.84 (0.96, 3.54) | 96.3 | 0 |  |
| Socio-demographic Index | 37 |  |  |  | 0.2863 |
| High | 26 | 1.79 (1.44, 2.24) | 96.4 | 0 |  |
| Middle | 11 | 1.51 (1.26, 1.80) | 93.6 | 0 |  |
| Sex | 37 |  |  |  | 0.7507 |
| Both | 22 | 1.74 (1.45, 2.08) | 97.5 | 0 |  |
| Male | 4 | 1.69 (1.27, 2.25) | 47.3 | 0.127 |  |
| Female | 11 | 1.52 (1.32, 1.76) | 22.9 | 0.226 |  |
| Mean age (years) | 33 |  |  |  | 0.8214 |
| >57.9 | 16 | 1.66 (1.24, 2.23) | 91.1 | 0 |  |
| <=57.9 | 17 | 1.73 (1.36, 2.18) | 97.7 | 0 |  |
| Sample Size | 37 |  |  |  | 0.5245 |
| >1,384 | 19 | 1.76 (1.39, 2.24) | 97.9 | 0 |  |
| <=1,384 | 18 | 1.59 (1.39, 1.82) | 75.9 | 0 |  |
| Confounding by smoking | 37 |  |  |  | 0.1247 |
| Yes | 23 | 1.55 (1.32, 1.83) | 96.0 | 0 |  |
| No | 14 | 1.94 (1.45, 2.59) | 94.8 | 0 |  |
| Risk of bias in NOS | 37 |  |  |  | 0.4616 |
| Low | 27 | 1.78 (1.49, 2.11) | 96.8 | 0 |  |
| Medium | 1 | 1.72 (1.35, 2.20) | n/a | n/a |  |
| High | 9 | 1.44 (1.23, 1.68) | 66.2 | 0.030 |  |

Note: n: number, RR: relative risk, CI: confidence interval, NOS: Newcastle-Ottawa Scale.

**Table S11. Subgroup and meta-regression for non-Hodgkin’s lymphoma**

|  |  |  |  | P-value | |
| --- | --- | --- | --- | --- | --- |
| Characteristic | **Studies, n** | **Pooled RR**  **(95% CI)** | ***I^2^,* %** | **Heterogeneity** | **Meta-regression** |
| non-Hodgkin’s lymphoma | 8 |  |  |  |  |
| Study design | 8 |  |  |  | 0.0317 |
| Case-control | 5 | 1.40 (1.21, 1.62) | 0 | 0.590 |  |
| Cohort | 3 | 1.84 (1.37, 2.48) | 49.4 | 0.139 |  |
| World Bank Country-income | 8 |  |  |  | 0.8486 |
| High | 7 | 1.61 (1.30, 1.98) | 56.9 | 0.030 |  |
| Upper middle | 1 | 1.70 (1.13, 2.56) | n/a | n/a |  |
| Region | 8 |  |  |  | 0.8932 |
| High-income North America | 1 | 1.62 (1.13, 2.33) | n/a | n/a |  |
| Southeast Asia, East Asia, and Oceania | 2 | 1.77 (1.26, 2.50) | 0 | 0.726 |  |
| Western Europe | 5 | 1.57 (1.19, 2.07) | 70.3 | 0.009 |  |
| Socio-demographic Index | 8 |  |  |  | 0.8486 |
| High | 7 | 1.61 (1.30, 1.98) | 56.9 | 0.030 |  |
| Middle | 1 | 1.70 (1.13, 2.56) | n/a | n/a |  |
| Mean age (years) | 6 |  |  |  | 0.2061 |
| >54.4 | 3 | 1.45 (1.13, 1.86) | 31.3 | 0.233 |  |
| <=54.4 | 3 | 1.82 (1.43, 2.33) | 42.2 | 0.177 |  |
| Sample Size | 8 |  |  |  | 0.9845 |
| >2,179 | 4 | 1.63 (1.18, 2.24) | 78.2 | 0.003 |  |
| <=2,179 | 4 | 1.61 (1.29, 2.02) | 0 | 0.960 |  |
| Confounding factors | 8 |  |  |  | 0.9918 |
| Adjusted | 7 | 1.62 (1.31, 2.00) | 57.2 | 0.029 |  |
| Unadjusted | 1 | 1.62 (1.13, 2.33) | n/a | n/a |  |
| Risk of bias in NOS | 8 |  |  |  | 0.9546 |
| Low | 6 | 1.64 (1.28, 2.11) | 64.1 | 0.016 |  |
| Medium | 1 | 1.48 (0.98, 2.25) | n/a | n/a |  |
| High | 1 | 1.62 (1.13, 2.33) | n/a | n/a |  |

Note: n: number, RR: relative risk, CI: confidence interval, NOS: Newcastle-Ottawa Scale.

**Table S12. Subgroup and meta-regression for leukaemia**

|  |  |  |  | P-value | |
| --- | --- | --- | --- | --- | --- |
| Characteristic | **Studies, n** | **Pooled RR**  **(95% CI)** | ***I^2^,* %** | **Heterogeneity** | **Meta-regression** |
| Leukaemia | 8 |  |  |  |  |
| Study design | 8 |  |  |  | 0.0261 |
| Case-control | 5 | 1.34 (1.06, 1.70) | 18.8 | 0.295 |  |
| Cohort | 3 | 2.02 (1.54, 2.66) | 0 | 0.870 |  |
| World Bank Country-income | 8 |  |  |  | 0.8952 |
| High | 7 | 1.54 (1.18, 2.03) | 42.6 | 0.107 |  |
| Upper middle | 1 | 1.61 (1.13, 2.29) | n/a | n/a |  |
| Region | 8 |  |  |  | 0.5346 |
| Central Europe, Eastern Europe, and Central Asia | 1 | 1.94 (1.41, 2.67) | n/a | n/a |  |
| High-income North America | 2 | 1.34 (0.96, 1.86) | 23.2 | 0.254 |  |
| Southeast Asia, East Asia, and Oceania | 2 | 1.74 (1.27, 2.38) | 0 | 0.341 |  |
| Western Europe | 3 | 1.36 (0.67, 2.78) | 45.6 | 0.159 |  |
| Socio-demographic Index | 8 |  |  |  | 0.8952 |
| High | 7 | 1.54 (1.18, 2.03) | 42.6 | 0.107 |  |
| Middle | 1 | 1.61 (1.13, 2.29) | n/a | n/a |  |
| Mean age (years) | 7 |  |  |  | 0.6920 |
| >51.6 | 3 | 1.45 (0.85, 2.47) | 61.7 | 0.074 |  |
| <=51.6 | 4 | 1.60 (1.23, 2.08) | 37.8 | 0.185 |  |
| Sample Size | 8 |  |  |  | 0.5266 |
| >2,111 | 4 | 1.67 (1.07, 2.62) | 58.1 | 0.067 |  |
| <=2,111 | 4 | 1.44 (1.16, 1.80) | 0 | 0.534 |  |
| Confounding factors | 8 |  |  |  | 0.1451 |
| Adjusted | 7 | 1.67 (1.34, 2.07) | 18.6 | 0.290 |  |
| Unadjusted | 1 | 1.15 (0.78, 1.68) | n/a | n/a |  |
| Risk of bias in NOS | 8 |  |  |  | 0.3418 |
| Low | 6 | 1.67 (1.27, 2.20) | 31.7 | 0.198 |  |
| High | 2 | 1.34 (0.96, 1.86) | 23.2 | 0.254 |  |

Note: n: number, RR: relative risk, CI: confidence interval, NOS: Newcastle-Ottawa Scale.

**Table S13. Subgroup and meta-regression for multiple myeloma**

|  |  |  |  | P-value | |
| --- | --- | --- | --- | --- | --- |
| Characteristic | **Studies, n** | **Pooled RR**  **(95% CI)** | ***I^2^,* %** | **Heterogeneity** | **Meta-regression** |
| Multiple myeloma | 4 |  |  |  |  |
| Study design | 4 |  |  |  | 0.3760 |
| Case-control | 3 | 1.31 (0.78, 2.18) | 44.3 | 0.166 |  |
| Cohort | 1 | 2.43 (0.79, 7.45) | n/a | n/a |  |
| Region | 4 |  |  |  | 0.8213 |
| High-income North America | 1 | 1.39 (0.96, 2.01) | n/a | n/a |  |
| Southeast Asia, East Asia, and Oceania | 1 | 2.43 (0.79, 7.45) | n/a | n/a |  |
| Western Europe | 2 | 1.26 (0.41, 3.89) | 70.7 | 0.065 |  |
| Mean age (years) | 4 |  |  |  | 0.1115 |
| >58 | 2 | 2.35 (1.15, 4.80) | 0 | 0.941 |  |
| <=58 | 2 | 1.11 (0.61, 2.03) | 51.5 | 0.151 |  |
| Sample Size | 4 |  |  |  | 0.5613 |
| >2,874 | 2 | 1.24 (0.39, 4.02) | 66.0 | 0.090 |  |
| <=2,874 | 2 | 1.49 (1.06, 2.10) | 0 | 0.320 |  |
| Confounding factors | 4 |  |  |  | 0.9062 |
| Adjusted | 3 | 1.51 (0.66, 3.44) | 56.6 | 0.100 |  |
| Unadjusted | 1 | 1.39 (0.96, 2.01) | n/a | n/a |  |
| Risk of bias in NOS | 4 |  |  |  | 0.9062 |
| Low | 3 | 1.51 (0.66, 3.44) | 56.6 | 0.100 |  |
| High | 1 | 1.39 (0.96, 2.01) | n/a | n/a |  |

Note: n: number, RR: relative risk, CI: confidence interval, NOS: Newcastle-Ottawa Scale.

We used visual inspection of funnel plot and Egger’s test to detect funnel plot asymmetric,^53^ which suggests the presence of publication bias and small-study effects. We only considered cancer sites with more than ten studies, given the power of Egger’s test to detect real asymmetry is low when number of studies is small. To address funnel plot asymmetry, we conducted trim and fill method to evaluate the number of missing studies and their influence on the pooled estimates.

The results showed that publication bias was not evident for lung cancer (Figure S10). The trim and fill method in a random-effects model suggested that overall estimates were not greatly modified by publication bias (Table S14).

**Table S14. Pooled estimates of cancer risk, publication bias, and trim-and fill estimates; by cancer site**

| Cancer site | Studies, n | Pooled estimate | Publication bias | Trim and fill estimate | |
| --- | --- | --- | --- | --- | --- |
|  |  | **RR (95% CI)** | ***P*-bias test** | **Missing**  **studies, n** | **RR (95% CI)** |
| Lung cancer | 37 | 1.69 (1.46, 1.95) | 0.533 | 0 | 1.69 (1.46, 1.95) |
| Lung cancer, adjusted for smoking | 23 | 1.55 (1.31, 1.83) | 0.442 | 0 | 1.55 (1.31, 1.83) |
| Lung cancer, never-smokers | 14 | 1.64 (1.41, 1.91) | 0.976 | 0 | 1.64 (1.41, 1.91) |

Note: n: number, RR: relative risk, CI: confidence interval.

**Figure S10. Funnel plot of lung cancer, lung cancer (adjusted for smoking), and lung cancer of never-smokers**

| 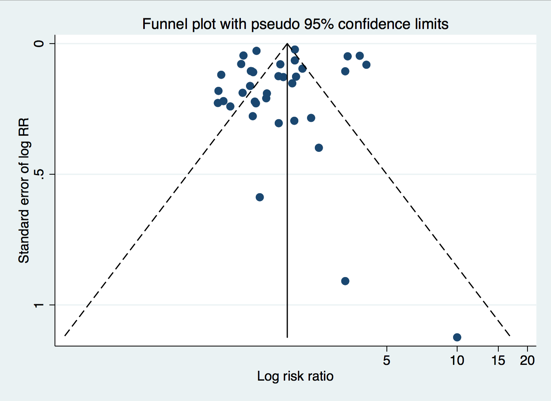 | 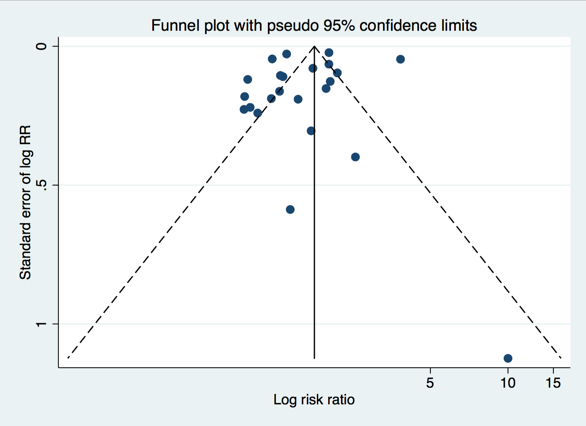 | 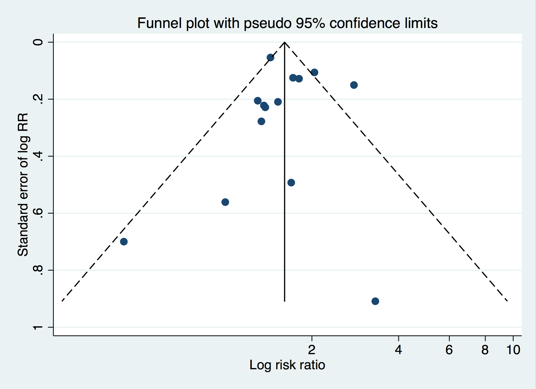 |
| --- | --- | --- |
| Lung cancer | Lung cancer,  adjusted for smoking | Lung cancer,  never-smokers |

Note: RR: relative risk.

**Table S15. Sensitivity analyses with summary estimates by with versus without dropping highly influential studies, by cancer type**

| Outcome | (Before sensitivity analyses) | | | | (Excluded highly influential studies) | | | |  |
| --- | --- | --- | --- | --- | --- | --- | --- | --- | --- |
|  | **Studies, n** | **Pooled estimate** | **Heterogeneity** | | **Studies, n** | **Pooled estimate** | **Heterogeneity** | | **Excluded**  **study** |
|  |  | **RR**  **(95% CI)** | ***I^2^* (%)** | **p** |  | **RR**  **(95% CI)** | ***I^2^***  **(%)** | **p** | **Reference** |
| Lung cancer | 37 | 1.69  (1.46, 1.95) | 95.9 | 0 | 32 | 1.61  (1.38, 1.87) | 89.7 | 0 | 7, 8,  9, 18, 49 |
| Lung cancer  (never-smokers) | 11 | 1.66  (1.36, 2.02) | 67.3 | 0.001 | 9 | 1.62  (1.35, 1.95) | 47.4 | 0.034 | 7, 8 |
| Hodgkin’s lymphoma | 6 | 2.19  (1.62, 2.97) | 0 | 0.557 | 4 | 2.32  (1.42, 3.78) | 22.2 | 0.277 | 9, 35 |
| Non-Hodgkin’s lymphoma | 8 | 1.61  (1.34, 1.94) | 50.2 | 0.050 | 6 | 1.58  (1.32, 1.90) | 0 | 0.956 | 9, 35 |
| Leukaemia | 8 | 1.55  (1.25, 1.93) | 33.2 | 0.163 | 6 | 1.54  (1.26, 1.89) | 0 | 0.472 | 6, 41 |

Note: n: number, RR: relative risk, CI: confidence interval, p: p-value.

**Table S16. Proportion of cancer in 2015 attributable to tuberculosis in male, female and, both sexes; by country**

|  | Male | | Female | | Both sexes | |
| --- | --- | --- | --- | --- | --- | --- |
|  | **TB-related**  **cancers** | **All cancers** | **TB-related**  **cancers** | **All cancers** | **TB-related**  **cancers** | **All cancers** |
|  | **PAF, %**  **(95% CI)** | **PAF, %**  **(95% CI)** | **PAF, %**  **(95% CI)** | **PAF, %**  **(95% CI)** | **PAF, %**  **(95% CI)** | **PAF, %**  **(95% CI)** |
| Australasia |  |  |  |  |  |  |
| Australia | 7.04  (2.97–13.11) | 0.71  (0.30–1.32) | 6.95  (2.75–13.64) | 0.76  (0.30–1.50) | 7.01  (2.88–13.32) | 0.73  (0.30–1.38) |
| New Zealand | 7.41  (3.12–13.80) | 1.01  (0.43–1.88) | 7.39  (2.92–14.45) | 0.97  (0.38–1.90) | 7.40  (3.03–14.08) | 0.99  (0.41–1.89) |
| Central Europe, Eastern Europe, and Central Asia |  |  |  |  |  |  |
| Albania | 11.55  (5.44–19.61) | 2.80  (1.32–4.76) | 11.05  (5.10–19.00) | 1.23  (0.57–2.11) | 11.43  (5.36–19.47) | 2.18  (1.02–3.72) |
| Armenia | 13.63  (6.47–22.88) | 3.22  (1.53–5.40) | 12.69  (5.86–21.67) | 0.96  (0.44–1.63) | 13.41  (6.33–22.60) | 2.12  (1.00–3.58) |
| Azerbaijan | 13.34  (6.20–22.95) | 2.45  (1.14–4.22) | 12.57  (5.67–21.94) | 0.87  (0.39–1.53) | 13.15  (6.07–22.70) | 1.71  (0.79–2.96) |
| Belarus | 20.42  (9.98–32.74) | 4.40  (2.15–7.06) | 19.16  (9.16–31.07) | 1.53  (0.73–2.48) | 20.09  (9.77–32.31) | 3.01  (1.46–4.84) |
| Bosnia and Herzegovina | 14.03  (6.75–23.38) | 3.97  (1.91–6.62) | 13.40  (6.36–22.50) | 1.49  (0.71–2.50) | 13.88  (6.66–23.17) | 2.88  (1.38–4.81) |
| Bulgaria | 16.09  (7.72–26.62) | 3.44  (1.65–5.69) | 15.37  (7.28–25.67) | 1.34  (0.63–2.23) | 15.89  (7.60–26.36) | 2.42  (1.16–4.02) |
| Croatia | 16.06  (7.79–26.31) | 3.65  (1.77–5.98) | 15.40  (7.42–25.42) | 2.00  (0.96–3.30) | 15.83  (7.66–26.01) | 2.86  (1.39–4.70) |
| Czech Republic | 15.70  (7.53–25.89) | 2.79  (1.34–4.59) | 14.88  (7.09–24.73) | 1.91  (0.91–3.18) | 15.39  (7.36–25.46) | 2.39  (1.15–3.96) |
| Estonia | 17.11  (8.31–27.83) | 3.11  (1.51–5.06) | 15.95  (7.62–6.14) | 2.14  (1.02–3.50) | 16.61  (8.01–27.11) | 2.62  (1.26–4.28) |
| Georgia | 13.69  (6.50–23.09) | 2.76  (1.31–4.65) | 13.13  (6.01–22.56) | 0.88  (0.40–1.51) | 13.57  (6.39–22.97) | 1.89  (0.89–3.19) |
| Hungary | 16.58  (7.98–27.17) | 3.68  (1.77–6.03) | 15.58  (7.52–25.61) | 2.47  (1.19–4.06) | 16.18  (7.80–26.55) | 3.10  (1.49–5.08) |
| Kazakhstan | 14.98  (7.17–25.03) | 2.86  (1.37–4.79) | 14.21  (6.57–24.21) | 0.89  (0.41–1.52) | 14.78  (7.02–24.82) | 1.84  (0.87–3.09) |
| Kyrgyzstan | 13.70  (6.42–23.20) | 2.01  (0.94–3.41) | 12.84  (5.81–22.23) | 0.77  (0.35–1.34) | 13.46  (6.25–22.93) | 1.41  (0.65–2.39) |
| Latvia | 16.29  (7.92–26.61) | 3.57  (1.74–5.84) | 15.30  (7.21–25.42) | 1.65  (0.78–2.73) | 15.95  (7.68–26.21) | 2.58  (1.24–4.24) |
| Lithuania | 16.71  (8.05–27.36) | 3.08  (1.48–5.04) | 15.70  (7.39–26.10) | 1.73  (0.81–2.87) | 16.37  (7.83–26.94) | 2.46  (1.18–4.04) |
| Macedonia | 15.04  (7.23–24.99) | 3.58  (1.72–5.95) | 14.44  (6.77–24.37) | 1.13  (0.53–1.91) | 14.91  (7.13–24.85) | 2.46  (1.18–4.11) |
| Moldova | 15.83  (7.54–26.34) | 3.16  (1.50–5.26) | 15.07  (7.05–25.38) | 1.13  (0.53–1.90) | 15.64  (7.41–26.10) | 2.19  (1.04–3.66) |
| Mongolia | 11.78  (5.54–20.11) | 1.59  (0.75–2.71) | 11.01  (5.05–19.06) | 0.61  (0.28–1.06) | 11.59  (5.42–19.85) | 1.15  (0.54–1.97) |
| Montenegro | 14.15  (6.75–23.67) | 4.14  (1.98–6.92) | 13.74  (6.46–23.18) | 1.84  (0.86–3.11) | 14.04  (6.67–23.53) | 3.08  (1.46–5.16) |
| Poland | 16.06  (7.74–26.43) | 3.89  (1.87–6.40) | 15.41  (7.34–25.68) | 2.14  (1.02–3.57) | 15.83  (7.60–26.17) | 3.05  (1.47–5.04) |
| Romania | 16.55  (7.99–27.16) | 3.45  (1.67–5.67) | 15.77  (7.50–26.17) | 1.54  (0.73–2.55) | 16.34  (7.86–26.89) | 2.61  (1.25–4.29) |
| Russian Federation | 19.05  (9.37–30.86) | 4.11  (2.02–6.66) | 18.30  (8.72–29.97) | 1.37  (0.65–2.25) | 18.84  (9.19–30.60) | 2.64  (1.29–4.29) |
| Serbia | 15.14  (7.29–25.06) | 3.62  (1.74–5.98) | 14.46  (6.90–24.18) | 1.82  (0.87–3.05) | 14.93  (7.17–24.79) | 2.80  (1.34–4.64) |
| Slovakia | 15.63  (7.43–25.95) | 3.43  (1.63–5.70) | 15.14  (7.10–25.37) | 1.86  (0.87–3.12) | 15.46  (7.32–25.75) | 2.68  (1.27–4.47) |
| Slovenia | 15.08  (7.18–25.01) | 3.33  (1.58–5.52) | 14.16  (6.77–23.52) | 2.11  (1.01–3.51) | 14.76  (7.04–24.48) | 2.78  (1.33–4.61) |
| Tajikistan | 11.66  (5.22–20.41) | 1.25  (0.56–2.19) | 11.13  (4.93–19.82) | 0.80  (0.35–1.42) | 11.48  (5.12–20.20) | 1.05  (0.47–1.85) |
| Turkmenistan | 12.40  (5.65–21.50) | 1.55  (0.71–2.69) | 11.73  (5.19–20.80) | 0.63  (0.28–1.12) | 12.18  (5.50–21.27) | 1.06  (0.48–1.85) |
| Ukraine | 18.69  (9.06–30.30) | 4.16  (2.02–6.75) | 17.67  (8.42–29.04) | 1.34  (0.64–2.20) | 18.43  (8.90–29.98) | 2.77  (1.34–4.50) |
| Uzbekistan | 11.83  (5.35–20.69) | 1.85  (0.84–3.23) | 11.42  (5.03–20.30) | 0.75  (0.33–1.34) | 11.69  (5.24–20.56) | 1.25  (0.56–2.19) |
| High-income Asia Pacific |  |  |  |  |  |  |
| Brunei | 9.64  (4.30–17.24) | 1.91  (0.85–3.42) | 11.38  (4.95–20.53) | 1.67  (0.73–3.02) | 10.43  (4.59–18.73) | 1.79  (0.79–3.21) |
| Japan | 8.40  (4.17–13.84) | 1.67  (0.83–2.75) | 9.16  (4.52–15.11) | 1.33  (0.66–2.19) | 8.66  (4.29–14.28) | 1.53  (0.76–2.52) |
| Singapore | 7.55  (3.39–13.53) | 1.72  (0.77–3.09) | 8.46  (3.74–15.22) | 1.24  (0.55–2.22) | 7.91  (3.53–14.20) | 1.47  (0.66–2.65) |
| South Korea | 10.23  (4.70–17.68) | 2.09  (0.96–3.62) | 11.19  (5.06–19.38) | 1.65  (0.74–2.85) | 10.56  (4.82–18.26) | 1.90  (0.87–3.29) |
| High-income North America |  |  |  |  |  |  |
| Canada | 5.29  (2.54–9.01) | 1.20  (0.57–2.04) | 3.90  (1.85–6.75) | 0.86  (0.41–1.49) | 4.65  (2.22–7.97) | 1.04  (0.50–1.78) |
| Greenland | 6.66  (3.26–11.19) | 1.87  (0.92–3.15) | 5.03  (2.45–8.53) | 1.18  (0.58–2.01) | 6.00  (2.93–10.12) | 1.57  (0.76–2.64) |
| United States | 5.71  (2.80–9.55) | 0.97  (0.47–1.61) | 4.16  (2.03–7.02) | 0.68  (0.33–1.14) | 5.01  (2.45–8.41) | 0.83  (0.41–1.40) |
| Latin America and Caribbean |  |  |  |  |  |  |
| Antigua and Barbuda | 16.08  (7.07–28.05) | 1.32  (0.58–2.31) | 15.01  (6.44–26.67) | 0.96  (0.41–1.70) | 15.65  (6.82–27.50) | 1.16  (0.50–2.03) |
| Barbados | 16.14  (7.17–27.92) | 1.29  (0.57–2.22) | 15.33  (6.69–26.97) | 1.05  (0.46–1.85) | 15.78  (6.95–27.49) | 1.17  (0.52–2.04) |
| Belize | 16.64  (7.42–28.70) | 3.01  (1.34–5.19) | 15.81  (6.78–28.05) | 1.37  (0.59–2.44) | 16.35  (7.20–28.48) | 2.16  (0.95–3.76) |
| Bermuda | 15.91  (7.15–27.33) | 2.48  (1.11–4.26) | 15.11  (6.72–26.22) | 1.53  (0.68–2.65) | 15.64  (7.00–26.95) | 2.05  (0.92–3.54) |
| Bolivia | 14.13  (6.48–24.13) | 1.98  (0.91–3.39) | 13.24  (5.98–22.92) | 1.13  (0.51–1.96) | 13.73  (6.25–23.58) | 1.49  (0.68–2.56) |
| Brazil | 17.12  (8.43–27.65) | 1.93  (0.95–3.11) | 15.61  (7.58–25.64) | 1.36  (0.66–2.24) | 16.47  (8.07–26.79) | 1.65  (0.81–2.68) |
| Colombia | 16.30  (7.30–27.99) | 2.01  (0.90–3.45) | 16.76  (7.52–28.71) | 1.42  (0.64–2.43) | 16.50  (7.39–28.30) | 1.70  (0.76–2.92) |
| Costa Rica | 16.56  (7.36–28.52) | 1.78  (0.79–3.06) | 17.25  (7.63–29.74) | 1.27  (0.56–2.18) | 16.83  (7.47–28.99) | 1.53  (0.68–2.64) |
| Cuba | 15.71  (7.24–26.63) | 2.95  (1.36–5.00) | 15.28  (6.95–26.30) | 1.99  (0.91–3.43) | 15.55  (7.13–26.51) | 2.51  (1.15–4.28) |
| Dominica | 16.71  (7.48–28.60) | 1.78  (0.80–3.04) | 15.50  (6.80–27.10) | 1.47  (0.64–2.57) | 16.28  (7.24–28.08) | 1.66  (0.74–2.86) |
| Dominican Republic | 14.52  (6.53–25.01) | 1.69  (0.76–2.91) | 13.97  (6.20–24.34) | 1.43  (0.63–2.49) | 14.30  (6.39–24.73) | 1.57  (0.70–2.72) |
| Ecuador | 13.38  (6.04–23.13) | 1.67  (0.75–2.89) | 12.52  (5.59–21.91) | 1.06  (0.47–1.85) | 13.00  (5.84–22.59) | 1.34  (0.60–2.32) |
| El Salvador | 15.40  (6.66–27.03) | 2.10  (0.91–3.69) | 15.91  (6.91–27.85) | 1.33  (0.58–2.33) | 15.64  (6.78–27.41) | 1.65  (0.71–2.89) |
| Grenada | 16.99  (7.59–29.34) | 2.29  (1.03–3.96) | 15.72  (6.84–27.55) | 1.58  (0.69–2.77) | 16.48  (7.29–28.62) | 1.96  (0.87–3.40) |
| Guatemala | 14.79  (6.33–26.18) | 1.76  (0.75–3.11) | 15.47  (6.52–27.64) | 1.08  (0.45–1.93) | 15.08  (6.41–26.82) | 1.37  (0.58–2.44) |
| Guyana | 18.12  (7.92–31.36) | 2.04  (0.89–3.52) | 17.18  (7.35–30.19) | 1.20  (0.51–2.10) | 17.71  (7.67–30.85) | 1.57  (0.68–2.73) |
| Haiti | 15.97  (7.06–27.71) | 3.17  (1.40–5.51) | 15.24  (6.45–27.18) | 1.67  (0.71–2.98) | 15.65  (6.79–27.48) | 2.29  (0.99–4.03) |
| Honduras | 15.73  (6.72–27.82) | 2.75  (1.17–4.87) | 16.43  (6.92–29.18) | 1.97  (0.83–3.50) | 16.06  (6.81–28.46) | 2.31  (0.98–4.09) |
| Jamaica | 15.80  (7.13–27.16) | 2.78  (1.25–4.78) | 15.13  (6.63–26.51) | 1.32  (0.58–2.31) | 15.59  (6.97–26.95) | 2.07  (0.92–3.57) |
| Mexico | 19.41  (9.59–30.98) | 1.62  (0.80–2.58) | 20.24  (9.88–32.35) | 1.16  (0.57–1.86) | 19.74  (9.70–31.53) | 1.39  (0.68–2.22) |
| Nicaragua | 15.20  (6.62–26.74) | 1.91  (0.83–3.36) | 15.87  (6.84–27.91) | 1.04  (0.45–1.82) | 15.48  (6.71–27.22) | 1.41  (0.61–2.48) |
| Panama | 15.73  (7.04–27.19) | 1.68  (0.75–2.91) | 16.35  (7.22–28.29) | 1.18  (0.52–2.04) | 15.96  (7.11–27.60) | 1.45  (0.65–2.50) |
| Paraguay | 17.50  (7.98–29.63) | 3.45  (1.57–5.85) | 15.72  (6.98–27.29) | 1.30  (0.58–2.25) | 16.94  (7.67–28.90) | 2.33  (1.05–3.98) |
| Peru | 13.68  (6.24–23.48) | 1.78  (0.81–3.06) | 12.79  (5.79–22.22) | 1.18  (0.53–2.04) | 13.26  (6.03–22.89) | 1.44  (0.66–2.49) |
| Puerto Rico | 16.97  (7.69–28.94) | 2.49  (1.13–4.25) | 16.19  (7.22–27.90) | 1.79  (0.80–3.08) | 16.67  (7.51–28.54) | 2.17  (0.98–3.72) |
| Saint Lucia | 17.78  (7.96–30.41) | 2.13  (0.95–3.64) | 16.59  (7.26–28.84) | 1.46  (0.64–2.54) | 17.34  (7.70–29.83) | 1.83  (0.81–3.15) |
| Saint Vincent and the Grenadines | 17.05  (7.55–29.43) | 2.03  (0.90–3.50) | 15.87  (6.84–27.93) | 1.37  (0.59–2.40) | 16.59  (7.27–28.84) | 1.72  (0.75–2.98) |
| Suriname | 16.16  (7.19–27.90) | 2.64  (1.18–4.56) | 15.21  (6.64–26.71) | 1.47  (0.64–2.59) | 15.80  (6.98–27.45) | 2.05  (0.91–3.56) |
| The Bahamas | 16.44  (7.31–28.51) | 1.67  (0.74–2.90) | 15.70  (6.79–27.63) | 0.98  (0.42–1.73) | 16.14  (7.10–28.15) | 1.31  (0.58–2.28) |
| Trinidad and Tobago | 19.51  (8.91–32.76) | 2.29  (1.05–3.85) | 18.76  (8.31–32.08) | 1.14  (0.51–1.95) | 19.26  (8.71–32.53) | 1.73  (0.78–2.92) |
| Venezuela | 17.48  (7.89–29.90) | 2.21  (1.00–3.78) | 17.95  (8.12–30.45) | 1.54  (0.70–2.61) | 17.68  (7.99–30.13) | 1.87  (0.85–3.19) |
| Virgin Islands, U.S. | 16.32  (7.35–27.91) | 1.67  (0.75–2.86) | 15.60  (6.91–27.06) | 1.71  (0.76–2.96) | 16.04  (7.18–27.58) | 1.68  (0.75–2.90) |
| North Africa and Middle East |  |  |  |  |  |  |
| Afghanistan | 20.48  (8.31–36.28) | 6.91  (2.80–12.25) | 19.17  (7.33–35.38) | 2.98  (1.14–5.51) | 19.96  (7.92–35.92) | 4.60  (1.82–8.27) |
| Algeria | 11.89  (4.65–22.91) | 2.77  (1.08–5.33) | 11.43  (4.34–22.35) | 1.00  (0.38–1.96) | 11.74  (4.55–22.73) | 1.77  (0.69–3.43) |
| Bahrain | 22.52  (6.74–42.79) | 5.46  (1.63–10.38) | 21.74  (6.62–42.10) | 2.16  (0.66–4.19) | 22.28  (6.70–42.58) | 3.75  (1.13–7.16) |
| Egypt | 20.70  (7.79–38.08) | 3.25  (1.22–5.97) | 18.82  (6.76–35.66) | 1.93  (0.69–3.66) | 20.05  (7.43–37.24) | 2.66  (0.99–4.94) |
| Iran | 11.76  (5.24–20.72) | 2.34  (1.04–4.13) | 13.66  (6.12–3.66) | 1.66  (0.74–2.87) | 12.40  (5.54–21.71) | 2.03  (0.91–3.56) |
| Iraq | 21.53  (6.41–41.48) | 6.76  (2.01–13.02) | 20.70  (6.07–40.76) | 2.97  (0.87–5.84) | 21.23  (6.29–41.22) | 4.69  (1.39–9.10) |
| Jordan | 5.72  (1.73–13.72) | 1.66  (0.50–3.99) | 4.84  (1.57–11.06) | 0.60  (0.19–1.36) | 5.43  (1.68–12.86) | 1.10  (0.34–2.60) |
| Kuwait | 21.63  (6.34–42.28) | 4.85  (1.42–9.48) | 20.96  (6.19–41.55) | 1.85  (0.55–3.67) | 21.40  (6.28–42.02) | 3.11  (0.91–6.11) |
| Lebanon | 13.21  (5.41–24.47) | 3.14  (1.29–5.82) | 13.12  (5.35–24.40) | 2.14  (0.87–3.99) | 13.18  (5.39–24.44) | 2.70  (1.11–5.01) |
| Libya | 20.27  (6.06–39.89) | 5.78  (1.73–11.38) | 21.12  (6.10–42.24) | 2.58  (0.74–5.16) | 20.51  (6.07–40.55) | 4.25  (1.26–8.41) |
| Morocco | 21.66  (6.59–41.31) | 7.23  (2.20–13.79) | 21.04  (6.25–41.27) | 1.77  (0.53–3.48) | 21.51  (6.51–41.30) | 4.20  (1.27–8.05) |
| Oman | 21.40  (6.31–42.05) | 5.41  (1.60–10.64) | 20.18  (5.84–40.77) | 2.26  (0.65–4.57) | 21.13  (6.21–41.77) | 4.20  (1.23–8.30) |
| Palestine | 19.16  (5.45–38.80) | 6.75  (1.92–13.67) | 18.31  (5.08–38.17) | 2.76  (0.77–5.75) | 18.92  (5.34–38.62) | 4.84  (1.37–9.89) |
| Qatar | 21.77  (6.40–42.26) | 5.02  (1.47–9.74) | 20.64  (5.99–41.18) | 1.38  (0.40–2.76) | 21.57  (6.33–42.07) | 3.45  (1.01–6.73) |
| Saudi Arabia | 21.24  (10.93–32.71) | 4.21  (2.16–6.48) | 19.24  (9.83–29.94) | 2.10  (1.07–3.26) | 20.51  (10.53–31.71) | 3.13  (1.61–4.84) |
| Sudan | 33.32  (17.89–46.86) | 8.40  (4.51–11.82) | 32.04  (16.15–46.73) | 4.55  (2.29–6.64) | 32.79  (17.17–46.81) | 6.25  (3.27–8.92) |
| Syria | 19.52  (5.34–39.75) | 7.21  (1.97–14.69) | 18.32  (4.92–38.57) | 4.31  (1.16–9.08) | 19.08  (5.19–39.32) | 5.84  (1.59–12.02) |
| Tunisia | 21.51  (6.57–40.88) | 6.75  (2.06–12.82) | 21.01  (6.33–41.44) | 2.01  (0.61–3.97) | 21.41  (6.52–40.99) | 4.63  (1.41–8.86) |
| Turkey | 15.35  (6.60–27.38) | 4.85  (2.08–8.64) | 14.04  (6.00–25.26) | 1.71  (0.73–3.08) | 15.07  (6.47–26.92) | 3.54  (1.52–6.32) |
| United Arab Emirates | 17.82  (8.05–30.40) | 3.58  (1.62–6.11) | 15.61  (6.84–27.22) | 1.36  (0.59–2.37) | 17.38  (7.81–29.77) | 2.77  (1.24–4.74) |
| Yemen | 20.61  (5.99–40.64) | 6.56  (1.91–12.94) | 19.70  (5.55–40.28) | 2.93  (0.83–5.99) | 20.26  (5.82–40.50) | 4.49  (1.29–8.98) |
| South Asia |  |  |  |  |  |  |
| Bangladesh | 11.07  (5.33–18.65) | 3.98  (1.92–6.71) | 9.63  (4.40–16.81) | 1.47  (0.67–2.56) | 10.70  (5.09–18.18) | 2.86  (1.36–4.85) |
| Bhutan | 11.08  (4.83–19.91) | 1.71  (0.75–3.08) | 10.08  (4.36–18.16) | 0.91  (0.39–1.65) | 10.75  (4.67–19.34) | 1.35  (0.59–2.42) |
| India | 22.23  (11.75–33.61) | 3.60  (1.90–5.44) | 20.68  (10.63–31.90) | 1.54  (0.79–2.38) | 21.71  (11.38–33.04) | 2.53  (1.33–3.85) |
| Nepal | 17.50  (8.36–28.87) | 3.16  (1.51–5.20) | 16.00  (7.36–27.05) | 1.73  (0.79–2.92) | 16.87  (7.94–28.10) | 2.37  (1.11–3.94) |
| Pakistan | 15.94  (6.21–30.06) | 3.77  (1.47–7.11) | 14.73  (5.43–28.80) | 1.15  (0.42–2.25) | 15.64  (6.02–29.75) | 2.47  (0.95–4.70) |
| Southern Latin America |  |  |  |  |  |  |
| Argentina | 7.59  (3.25–14.05) | 1.33  (0.57–2.47) | 7.44  (2.94–14.52) | 0.73  (0.29–1.43) | 7.54  (3.14–14.22) | 1.03  (0.43–1.95) |
| Chile | 7.72  (3.30–14.28) | 0.98  (0.42–1.82) | 7.56  (3.00–14.82) | 0.80  (0.32–1.57) | 7.65  (3.17–14.52) | 0.90  (0.37–1.70) |
| Uruguay | 7.90  (3.39–14.51) | 1.58  (0.68–2.90) | 7.65  (3.05–14.78) | 0.76  (0.30–1.47) | 7.82  (3.29–14.59) | 1.20  (0.50–2.24) |
| Southeast Asia, East Asia, and Oceania |  |  |  |  |  |  |
| American Samoa | 19.00  (9.43–30.32) | 3.39  (1.68–5.41) | 18.27  (8.94–29.50) | 1.47  (0.72–2.37) | 18.70  (9.23–29.99) | 2.22  (1.10–3.57) |
| Cambodia | 19.17  (8.96–31.79) | 6.01  (2.81–9.97) | 17.94  (8.09–30.64) | 2.14  (0.96–3.65) | 18.75  (8.66–31.39) | 3.76  (1.74–6.29) |
| China | 18.44  (10.10–27.59) | 4.76  (2.61–7.13) | 16.77  (9.05–25.46) | 2.91  (1.57–4.42) | 17.94  (9.79–26.96) | 4.05  (2.21–6.09) |
| Federated States of Micronesia | 17.52  (8.54–28.35) | 4.80  (2.34–7.76) | 16.56  (7.90–27.24) | 1.54  (0.73–2.53) | 17.22  (8.34–28.00) | 2.93  (1.42–4.77) |
| Fiji | 19.58  (9.33–31.92) | 2.95  (1.41–4.82) | 18.10  (8.55–29.76) | 0.82  (0.39–1.34) | 19.02  (9.04–31.11) | 1.53  (0.73–2.50) |
| Guam | 17.06  (8.44–27.53) | 4.27  (2.12–6.90) | 15.72  (7.72–25.49) | 2.78  (1.36–4.50) | 16.58  (8.19–26.81) | 3.62  (1.79–5.85) |
| Indonesia | 17.84  (8.67–29.08) | 3.06  (1.49–5.00) | 16.80  (8.02–27.82) | 0.97  (0.46–1.61) | 17.49  (8.45–28.66) | 1.81  (0.88–2.97) |
| Kiribati | 21.07  (10.49–33.35) | 4.65  (2.31–7.36) | 20.01  (9.87–31.83) | 2.06  (1.01–3.27) | 20.74  (10.30–32.89) | 3.40  (1.69–5.38) |
| Laos | 16.94  (7.08–30.60) | 5.27  (2.20–9.52) | 15.49  (6.28–28.34) | 2.08  (0.84–3.81) | 16.43  (6.80–29.80) | 3.49  (1.44–6.33) |
| Malaysia | 18.44  (6.97–34.45) | 4.95  (1.87–9.25) | 17.38  (6.30–33.38) | 2.16  (0.78–4.15) | 18.08  (6.74–34.08) | 3.47  (1.29–6.54) |
| Maldives | 17.56  (6.65–33.10) | 4.14  (1.57–7.80) | 16.51  (5.83–32.52) | 1.40  (0.49–2.76) | 17.30  (6.44–32.96) | 2.81  (1.05–5.36) |
| Marshall Islands | 19.58  (9.67–31.41) | 5.41  (2.67–8.67) | 18.49  (8.81–30.24) | 1.66  (0.79–2.72) | 19.24  (9.40–31.04) | 3.21  (1.57–5.18) |
| Mauritius | 22.03  (8.60–38.85) | 3.72  (1.45–6.56) | 20.92  (7.90–38.15) | 1.57  (0.59–2.87) | 21.65  (8.36–38.61) | 2.56  (0.99–4.57) |
| Myanmar | 18.99  (7.32–35.05) | 6.97  (2.69–12.87) | 18.00  (6.53–34.57) | 1.78  (0.65–3.42) | 18.79  (7.16–34.95) | 4.45  (1.70–8.28) |
| North Korea | 16.59  (7.97–27.53) | 6.39  (3.07–10.60) | 15.45  (7.21–26.04) | 4.53  (2.12–7.64) | 16.12  (7.66–26.92) | 5.51  (2.61–9.19) |
| Northern Mariana Islands | 19.18  (9.25–31.11) | 5.47  (2.64–8.88) | 18.27  (8.79–29.73) | 2.05  (0.98–3.33) | 18.88  (9.10–30.64) | 3.54  (1.70–5.74) |
| Papua New Guinea | 18.95  (9.25–30.56) | 5.84  (2.85–9.42) | 17.91  (8.55–29.35) | 1.92  (0.91–3.14) | 18.57  (8.99–30.12) | 3.39  (1.64–5.49) |
| Philippines | 20.76  (9.58–34.41) | 5.38  (2.48–8.92) | 19.14  (8.62–32.44) | 2.04  (0.92–3.46) | 20.20  (9.24–33.73) | 3.50  (1.60–5.85) |
| Samoa | 19.75  (9.61–31.85) | 3.21  (1.56–5.18) | 18.74  (8.97–30.51) | 1.41  (0.68–2.30) | 19.36  (9.36–31.33) | 2.17  (1.05–3.52) |
| Seychelles | 21.72  (8.32–38.99) | 2.67  (1.02–4.79) | 20.71  (7.80–38.00) | 1.74  (0.65–3.19) | 21.36  (8.13–38.64) | 2.25  (0.86–4.08) |
| Solomon Islands | 17.58  (8.59–28.55) | 4.72  (2.31–7.67) | 16.69  (7.90–27.68) | 1.63  (0.77–2.71) | 17.29  (8.36–28.27) | 2.95  (1.43–4.82) |
| Sri Lanka | 12.89  (4.99–24.74) | 2.47  (0.96–4.75) | 11.55  (4.37–22.58) | 1.23  (0.46–2.40) | 12.41  (4.76–23.96) | 1.84  (0.71–3.56) |
| Taiwan | 20.86  (10.74–32.22) | 3.82  (1.97–5.91) | 20.38  (10.39–31.80) | 3.10  (1.58–4.84) | 20.67  (10.61–32.06) | 3.51  (1.80–5.44) |
| Thailand | 20.22  (7.87–36.51) | 4.78  (1.86–8.64) | 18.49  (6.99–34.48) | 2.64  (1.00–4.92) | 19.60  (7.55–35.78) | 3.75  (1.45–6.85) |
| Timor-Leste | 18.70  (7.08–35.07) | 4.52  (1.71–8.48) | 17.48  (6.39–33.64) | 2.23  (0.81–4.29) | 18.21  (6.80–34.49) | 3.24  (1.21–6.13) |
| Tonga | 17.63  (8.79–28.25) | 4.42  (2.20–7.08) | 16.82  (8.41–26.99) | 1.83  (0.91–2.93) | 17.35  (8.66–27.81) | 2.99  (1.49–4.79) |
| Vanuatu | 17.30  (8.44–28.01) | 4.90  (2.39–7.94) | 16.31  (7.76–26.90) | 1.73  (0.82–2.86) | 16.96  (8.21–27.63) | 3.07  (1.49–5.00) |
| Vietnam | 25.10  (10.90–41.17) | 7.98  (3.46–13.08) | 23.35  (10.13–39.10) | 5.42  (2.35–9.07) | 24.41  (10.60–40.35) | 6.77  (2.94–11.19) |
| Sub-Saharan Africa |  |  |  |  |  |  |
| Angola | 19.25  (8.80–32.40) | 2.99  (1.37–5.04) | 18.81  (8.50–32.03) | 1.44  (0.65–2.46) | 19.06  (8.67–32.24) | 2.07  (0.94–3.50) |
| Benin | 20.40  (8.28–36.28) | 3.23  (1.31–5.74) | 19.13  (7.61–34.78) | 1.43  (0.57–2.60) | 19.93  (8.03–35.73) | 2.24  (0.90–4.01) |
| Botswana | 21.85  (9.40–37.26) | 3.71  (1.60–6.34) | 19.16  (7.88–34.08) | 1.37  (0.56–2.43) | 21.02  (8.93–36.28) | 2.51  (1.07–4.33) |
| Burkina Faso | 22.94  (9.43–39.54) | 2.68  (1.10–4.63) | 21.70  (8.88–38.07) | 1.27  (0.52–2.22) | 22.41  (9.19–38.92) | 1.84  (0.76–3.20) |
| Burundi | 22.83  (9.71–39.20) | 2.98  (1.27–5.11) | 21.18  (8.91–36.84) | 1.49  (0.63–2.59) | 22.11  (9.36–38.17) | 2.10  (0.89–3.63) |
| Cameroon | 21.99  (9.44–37.53) | 3.26  (1.40–5.57) | 20.58  (8.63–35.87) | 1.56  (0.65–2.72) | 21.47  (9.14–36.92) | 2.35  (1.00–4.05) |
| Cape Verde | 21.27  (8.95–36.58) | 2.91  (1.23–5.01) | 20.57  (8.61–35.63) | 2.09  (0.87–3.62) | 21.01  (8.83–36.24) | 2.56  (1.07–4.41) |
| Central African Republic | 19.73  (9.12–32.96) | 3.59  (1.66–6.00) | 19.04  (8.57–32.40) | 1.31  (0.59–2.23) | 19.50  (8.94–32.77) | 2.28  (1.04–3.83) |
| Chad | 21.24  (8.70–37.25) | 3.29  (1.35–5.77) | 20.01  (7.98–35.96) | 1.56  (0.62–2.81) | 20.77  (8.43–36.77) | 2.35  (0.95–4.16) |
| Comoros | 19.19  (7.90–34.36) | 2.39  (0.98–4.27) | 17.85  (7.29–32.49) | 1.13  (0.46–2.05) | 18.65  (7.65–33.61) | 1.67  (0.69–3.02) |
| Congo | 19.11  (8.81–31.95) | 2.53  (1.17–4.23) | 18.74  (8.52–31.57) | 1.47  (0.67–2.48) | 18.93  (8.67–31.77) | 1.88  (0.86–3.16) |
| Cote d'Ivoire | 21.99  (9.09–38.24) | 3.07  (1.27–5.35) | 20.82  (8.23–37.42) | 1.75  (0.69–3.14) | 21.53  (8.75–37.92) | 2.38  (0.97–4.20) |
| Democratic Republic of the Congo | 18.56  (8.43–31.43) | 2.77  (1.26–4.68) | 18.07  (8.10–31.00) | 1.34  (0.60–2.29) | 18.36  (8.30–31.26) | 1.95  (0.88–3.31) |
| Djibouti | 24.46  (11.56–39.25) | 3.12  (1.47–5.01) | 22.61  (10.51–37.09) | 1.47  (0.69–2.42) | 23.79  (11.18–38.47) | 2.25  (1.06–3.65) |
| Equatorial Guinea | 20.24  (9.37–33.53) | 2.73  (1.26–4.52) | 19.71  (9.08–32.90) | 1.66  (0.76–2.77) | 20.02  (9.26–33.27) | 2.16  (1.00–3.60) |
| Eritrea | 19.94  (8.22–35.38) | 2.82  (1.16–5.00) | 18.58  (7.57–33.42) | 1.17  (0.47–2.10) | 19.37  (7.95–34.56) | 1.79  (0.74–3.20) |
| Ethiopia | 15.16  (6.48–27.13) | 2.01  (0.86–3.59) | 14.30  (6.08–25.88) | 0.92  (0.39–1.66) | 14.82  (6.32–26.64) | 1.38  (0.59–2.48) |
| Gabon | 20.87  (9.74–34.40) | 2.35  (1.10–3.88) | 20.32  (9.40–33.85) | 1.32  (0.61–2.20) | 20.64  (9.60–34.18) | 1.79  (0.83–2.96) |
| Ghana | 16.52  (6.64–30.51) | 2.71  (1.09–5.00) | 15.76  (6.17–29.61) | 0.92  (0.36–1.72) | 16.27  (6.48–30.21) | 1.65  (0.66–3.07) |
| Guinea | 19.44  (7.81–35.07) | 2.88  (1.15–5.19) | 18.01  (7.01–33.23) | 0.89  (0.35–1.65) | 19.03  (7.57–34.54) | 1.79  (0.71–3.24) |
| Guinea-Bissau | 21.50  (8.82–37.59) | 3.38  (1.39–5.91) | 20.20  (8.02–36.21) | 1.35  (0.54–2.42) | 21.04  (8.54–37.10) | 2.24  (0.91–3.95) |
| Kenya | 22.07  (10.92–34.82) | 2.29  (1.13–3.61) | 20.76  (10.28–32.96) | 1.65  (0.82–2.62) | 21.45  (10.61–33.94) | 1.94  (0.96–3.07) |
| Lesotho | 22.39  (9.43–38.20) | 5.37  (2.26–9.16) | 20.49  (8.23–36.38) | 1.56  (0.63–2.77) | 21.86  (9.10–37.70) | 3.29  (1.37–5.68) |
| Liberia | 21.51  (8.78–37.74) | 2.66  (1.08–4.66) | 20.42  (8.12–36.50) | 1.29  (0.51–2.30) | 21.07  (8.52–37.24) | 1.88  (0.76–3.32) |
| Madagascar | 22.47  (8.26–40.67) | 2.84  (1.04–5.13) | 21.71  (7.94–39.94) | 1.41  (0.52–2.60) | 22.16  (8.13–40.37) | 2.01  (0.74–3.67) |
| Malawi | 17.21  (8.09–28.77) | 1.61  (0.76–2.69) | 14.96  (6.96–25.43) | 0.81  (0.37–1.37) | 16.24  (7.60–27.33) | 1.15  (0.54–1.94) |
| Mali | 19.70  (7.86–35.63) | 1.84  (0.73–3.33) | 18.49  (7.21–34.07) | 1.02  (0.40–1.88) | 19.20  (7.59–34.98) | 1.39  (0.55–2.54) |
| Mauritania | 19.19  (7.68–34.59) | 2.74  (1.10–4.95) | 18.06  (7.16–33.06) | 1.25  (0.49–2.28) | 18.73  (7.47–33.97) | 1.87  (0.75–3.39) |
| Mozambique | 20.55  (8.58–36.17) | 2.61  (1.09–4.60) | 18.99  (7.85–33.92) | 1.12  (0.46–2.00) | 19.90  (8.28–35.23) | 1.71  (0.71–3.03) |
| Namibia | 21.96  (9.04–38.31) | 2.30  (0.95–4.01) | 20.44  (8.09–36.75) | 1.40  (0.55–2.51) | 21.30  (8.63–37.63) | 1.81  (0.73–3.20) |
| Niger | 19.20  (7.66–34.81) | 2.82  (1.12–5.11) | 17.75  (6.92–32.76) | 1.30  (0.51–2.40) | 18.69  (7.39–34.08) | 2.02  (0.80–3.67) |
| Nigeria | 24.14  (10.17–40.96) | 2.33  (0.98–3.95) | 23.12  (9.48–40.21) | 1.22  (0.50–2.13) | 23.77  (9.92–40.69) | 1.76  (0.74–3.02) |
| Rwanda | 22.54  (9.62–38.62) | 2.87  (1.23–4.92) | 21.12  (8.91–36.81) | 1.59  (0.67–2.77) | 21.85  (9.28–37.74) | 2.09  (0.89–3.61) |
| Sao Tome and Principe | 20.84  (8.74–36.08) | 5.01  (2.10–8.68) | 19.95  (8.23–35.44) | 1.69  (0.70–3.00) | 20.56  (8.58–35.88) | 3.13  (1.30–5.46) |
| Senegal | 20.10  (8.19–35.71) | 3.27  (1.33–5.81) | 19.04  (7.55–34.45) | 1.34  (0.53–2.42) | 19.70  (7.95–35.23) | 2.14  (0.86–3.83) |
| Sierra Leone | 23.30  (9.82–39.59) | 3.56  (1.50–6.05) | 22.14  (9.03–38.66) | 1.51  (0.61–2.63) | 22.86  (9.52–39.24) | 2.37  (0.98–4.06) |
| Somalia | 16.02  (6.45–29.56) | 2.05  (0.83–3.79) | 15.13  (6.00–28.37) | 0.84  (0.33–1.57) | 15.64  (6.26–29.06) | 1.29  (0.51–2.39) |
| South Africa | 27.73  (15.07–40.47) | 3.93  (2.14–5.74) | 25.93  (13.65–38.72) | 1.88  (0.99–2.81) | 27.08  (14.56–39.83) | 2.85  (1.53–4.20) |
| South Sudan | 21.38  (8.95–37.29) | 2.74  (1.15–4.77) | 19.82  (8.15–35.35) | 1.33  (0.55–2.37) | 20.72  (8.61–36.46) | 1.91  (0.79–3.36) |
| Swaziland | 21.66  (9.01–37.32) | 4.40  (1.83–7.59) | 19.78  (7.94–35.48) | 1.76  (0.71–3.16) | 21.06  (8.67–36.74) | 3.04  (1.25–5.31) |
| Tanzania | 19.98  (9.19–33.43) | 2.71  (1.25–4.53) | 18.25  (8.32–30.94) | 1.45  (0.66–2.46) | 19.28  (8.84–32.42) | 2.03  (0.93–3.41) |
| The Gambia | 25.26  (11.76–40.47) | 2.62  (1.22–4.20) | 23.55  (10.55–38.77) | 1.26  (0.56–2.07) | 24.71  (11.37–39.92) | 1.97  (0.90–3.18) |
| Togo | 20.37  (8.30–36.25) | 3.43  (1.40–6.10) | 19.17  (7.54–34.78) | 1.36  (0.54–2.47) | 19.95  (8.03–35.74) | 2.27  (0.91–4.07) |
| Uganda | 27.82  (13.63–43.11) | 3.65  (1.79–5.66) | 26.85  (13.00–42.08) | 2.05  (0.99–3.22) | 27.40  (13.36–42.66) | 2.74  (1.34–4.27) |
| Zambia | 24.45  (11.63–39.24) | 3.03  (1.44–4.87) | 22.16  (10.39–36.24) | 1.48  (0.69–2.41) | 23.56  (11.15–38.07) | 2.19  (1.04–3.54) |
| Zimbabwe | 15.84  (6.54–28.79) | 2.17  (0.89–3.93) | 14.85  (5.99–27.60) | 1.12  (0.45–2.08) | 15.43  (6.31–28.28) | 1.57  (0.64–2.88) |
| Western Europe |  |  |  |  |  |  |
| Andorra | 8.44  (3.00–17.68) | 1.53  (0.54–3.21) | 7.51  (2.68–15.91) | 1.00  (0.36–2.13) | 8.11  (2.88–17.05) | 1.31  (0.46–2.75) |
| Austria | 9.30  (3.35–19.33) | 1.76  (0.63–3.65) | 8.28  (2.96–17.30) | 1.34  (0.48–2.80) | 8.88  (3.18–18.49) | 1.57  (0.56–3.27) |
| Belgium | 8.27  (2.96–17.45) | 1.84  (0.66–3.88) | 7.47  (2.63–16.00) | 1.06  (0.37–2.26) | 8.00  (2.85–16.95) | 1.49  (0.53–3.15) |
| Cyprus | 8.60  (3.04–18.00) | 1.83  (0.65–3.84) | 7.82  (2.72–16.66) | 0.82  (0.29–1.75) | 8.38  (2.95–17.63) | 1.39  (0.49–2.91) |
| Denmark | 9.71  (3.46–20.00) | 1.95  (0.70–4.02) | 8.66  (3.11–17.95) | 1.72  (0.62–3.56) | 9.23  (3.30–19.06) | 1.84  (0.66–3.81) |
| Finland | 7.68  (2.75–16.21) | 1.25  (0.45–2.64) | 6.78  (2.43–14.44) | 0.88  (0.32–1.87) | 7.32  (2.62–15.51) | 1.08  (0.39–2.30) |
| France | 8.25  (2.90–17.42) | 1.51  (0.53–3.19) | 7.18  (2.52–15.24) | 0.89  (0.31–1.88) | 7.90  (2.78–16.71) | 1.25  (0.44–2.64) |
| Germany | 8.37  (3.02–17.67) | 1.49  (0.54–3.16) | 7.42  (2.66–15.73) | 1.05  (0.38–2.23) | 8.00  (2.88–16.92) | 1.30  (0.47–2.75) |
| Greece | 8.56  (3.08–17.74) | 1.89  (0.68–3.92) | 7.80  (2.76–16.37) | 0.92  (0.32–1.93) | 8.34  (2.99–17.36) | 1.48  (0.53–3.08) |
| Iceland | 6.71  (2.34–14.42) | 1.06  (0.37–2.27) | 6.02  (2.13–13.00) | 1.06  (0.38–2.30) | 6.37  (2.23–13.73) | 1.06  (0.37–2.28) |
| Ireland | 8.35  (2.94–17.58) | 1.33  (0.47–2.81) | 7.49  (2.68–15.88) | 1.09  (0.39–2.30) | 7.98  (2.83–16.85) | 1.22  (0.43–2.58) |
| Israel | 6.45  (2.27–13.91) | 1.22  (0.43–2.63) | 5.61  (1.96–12.21) | 0.67  (0.24–1.47) | 6.13  (2.15–13.26) | 0.95  (0.33–2.05) |
| Italy | 9.92  (4.22–18.38) | 1.83  (0.78–3.40) | 8.82  (3.65–16.56) | 1.11  (0.46–2.08) | 9.53  (4.02–17.73) | 1.51  (0.64–2.81) |
| Luxembourg | 8.26  (2.91–17.44) | 1.74  (0.62–3.68) | 7.45  (2.60–15.95) | 1.06  (0.37–2.27) | 7.95  (2.79–16.87) | 1.42  (0.50–3.01) |
| Malta | 6.99  (2.48–14.86) | 1.37  (0.49–2.91) | 6.47  (2.28–14.03) | 0.57  (0.20–1.24) | 6.86  (2.43–14.65) | 1.03  (0.36–2.20) |
| Netherlands | 7.98  (2.85–16.75) | 1.61  (0.57–3.37) | 7.30  (2.59–15.47) | 1.24  (0.44–2.64) | 7.70  (2.74–16.23) | 1.44  (0.51–3.04) |
| Norway | 7.29  (2.57–15.54) | 1.11  (0.39–2.36) | 6.45  (2.28–13.90) | 1.00  (0.35–2.16) | 6.93  (2.45–14.83) | 1.06  (0.38–2.28) |
| Portugal | 9.11  (3.25–18.84) | 1.40  (0.50–2.90) | 7.95  (2.84–16.47) | 0.82  (0.29–1.70) | 8.72  (3.12–18.06) | 1.15  (0.41–2.39) |
| Spain | 5.01  (1.79–10.65) | 1.02  (0.36–2.16) | 4.51  (1.57–9.89) | 0.50  (0.17–1.09) | 4.87  (1.73–10.44) | 0.80  (0.29–1.72) |
| Sweden | 9.46  (4.10–17.10) | 1.08  (0.47–1.95) | 8.40  (3.65–15.21) | 1.09  (0.47–1.96) | 8.96  (3.89–16.21) | 1.08  (0.47–1.96) |
| Switzerland | 8.04  (2.86–16.97) | 1.18  (0.42–2.49) | 7.25  (2.57–15.40) | 0.95  (0.34–2.02) | 7.73  (2.74–16.34) | 1.08  (0.38–2.29) |
| United Kingdom | 7.27  (2.99–13.75) | 1.34  (0.55–2.53) | 6.40  (2.65–12.14) | 1.16  (0.48–2.20) | 6.88  (2.84–13.04) | 1.26  (0.52–2.38) |

Note: TB: tuberculosis, PAF: population attributable fraction, CI: confidence interval.

**Table S17. Tuberculosis attributable cancer cases by region, country, and SDI; both sexes**

|  | **Hodgkin's lymphoma** | **Leukaemia** | **Lung cancer** | **Non-Hodgkin's lymphoma** | **TB-related cancer** |
| --- | --- | --- | --- | --- | --- |
|  | **Case**  **(95% CI)** | **Case**  **(95% CI)** | **Case**  **(95% CI)** | **Case**  **(95% CI)** | **Case**  **(95% CI)** |
| **Region** |  |  |  |  |  |
| **Australasia** | 107  (43–206) | 436  (154–886) | 1025  (442–1895) | 513  (217–968) | 2080  (855–3955) |
| Australia | 92  (37–177) | 360  (127–731) | 862  (372–1594) | 437  (185–824) | 1750  (720–3327) |
| New Zealand | 15  (6–29) | 76  (27–154) | 163  (70–301) | 76  (32–144) | 330  (135–628) |
| **Central Europe, Eastern Europe, and Central Asia** | 2275  (1106–3621) | 5200  (2105–9182) | 26 065  (12 930–42 249) | 4952  (2433–8106) | 38 492  (18 573–63 158) |
| Albania | 5  (2–8) | 14  (5–26) | 106  (51–178) | 7  (3–13) | 132  (62–225) |
| Armenia | 4  (2–6) | 24  (9–43) | 152  (74–253) | 13  (6–22) | 192  (91–324) |
| Azerbaijan | 20  (9–33) | 41  (16–77) | 212  (101–361) | 29  (14–51) | 302  (140–522) |
| Belarus | 45  (23–69) | 168  (69–289) | 645  (323–1024) | 152  (76–243) | 1010  (491–1625) |
| Bosnia and Herzegovina | 8  (4–13) | 32  (13–57) | 315  (154–522) | 23  (11–39) | 378  (181–631) |
| Bulgaria | 56  (27–90) | 98  (39–175) | 559  (274–917) | 144  (70–239) | 857  (409–1421) |
| Croatia | 39  (19–62) | 100  (41–176) | 508  (252–824) | 112  (55–184) | 759  (367–1246) |
| Czech Republic | 82  (40–133) | 316  (129–559) | 960  (478–1558) | 249  (123–408) | 1607  (769–2658) |
| Estonia | 9  (5–15) | 36  (15–63) | 106  (53–170) | 42  (21–67) | 193  (93–315) |
| Georgia | 17  (8–27) | 29  (12–54) | 158  (76–264) | 21  (10–36) | 225  (106–381) |
| Hungary | 33  (16–53) | 208  (85–367) | 1374  (676–2232) | 207  (102–338) | 1823  (879–2991) |
| Kazakhstan | 31  (15–51) | 78  (31–142) | 463  (226–767) | 50  (24–85) | 622  (295–1045) |
| Kyrgyzstan | 3  (2–5) | 14  (5–26) | 62  (30–103) | 8  (4–13) | 87  (40–148) |
| Latvia | 14  (7–23) | 45  (18–79) | 176  (88–285) | 42  (21–69) | 278  (134–456) |
| Lithuania | 20  (10–33) | 93  (38–162) | 234  (117–378) | 70  (35–114) | 418  (200–687) |
| Macedonia | 7  (3–12) | 17  (7–31) | 140  (68–232) | 12  (6–21) | 177  (85–295) |
| Moldova | 11  (5–18) | 21  (8–39) | 151  (73–250) | 23  (11–39) | 207  (98–346) |
| Mongolia | 1  (0–2) | 6  (2–11) | 51  (24–86) | 6  (3–11) | 64  (30–110) |
| Montenegro | 4  (2–6) | 6  (2–10) | 50  (24–84) | 4  (2–7) | 64  (30–107) |
| Poland | 227  (108–367) | 719  (290–1279) | 3577  (1767–5832) | 614  (301–1012) | 5138  (2466–8490) |
| Romania | 90  (43–144) | 260  (105–461) | 1641  (808–2672) | 257  (126–423) | 2248  (1081–3700) |
| Russian Federation | 1208  (592–1905) | 1840  (751–3218) | 9563  (4796–15 370) | 1960  (968–3184) | 14 572  (7108–23 676) |
| Serbia | 50  (24–82) | 100  (40–179) | 866  (423–1426) | 107  (52–179) | 1123  (540–1865) |
| Slovakia | 43  (20–69) | 141  (57–252) | 436  (215–713) | 134  (65–222) | 754  (357–1256) |
| Slovenia | 9  (4–14) | 60  (25–107) | 171  (85–280) | 82  (40–134) | 322  (154–535) |
| Tajikistan | 5  (2–8) | 14  (5–27) | 31  (15–53) | 9  (4–15) | 58  (26–103) |
| Turkmenistan | 8  (4–14) | 12  (4–22) | 36  (17–61) | 8  (4–14) | 64  (29–111) |
| Ukraine | 191  (94–297) | 627  (254–1096) | 3106  (1541–4988) | 499  (245–811) | 4422  (2135–7193) |
| Uzbekistan | 36  (17–62) | 79  (30–151) | 215  (101–368) | 65  (30–114) | 396  (178–696) |
| **High-income Asia Pacific** | 273  (127–465) | 1688  (673–3088) | 11 764  (5861–19 337) | 3194  (1563–5358) | 16 918  (8225–28 247) |
| Brunei | 2  (1–3) | 2  (1–4) | 9  (4–16) | 5  (2–8) | 17  (8–31) |
| Japan | 197  (93–332) | 1288  (524–2327) | 8787  (4467–14 260) | 2529  (1261–4193) | 12 801  (6346–21 111) |
| Singapore | 5  (2–9) | 21  (8–42) | 124  (57–218) | 39  (17–70) | 189  (84–339) |
| South Korea | 69  (30–121) | 377  (141–715) | 2844  (1333–4843) | 622  (283–1087) | 3912  (1787–6766) |
| **High-income North America** | 1092  (513–1925) | 2512  (1024–4594) | 13 008  (6541–21 420) | 4874  (2424–8184) | 21 487  (10 501–36 124) |
| Canada | 87  (40–156) | 279  (112–518) | 1139  (563–1909) | 520  (254–889) | 2025  (968–3472) |
| Greenland | 0  (0–0) | 0  (0–0) | 3  (1–4) | 0  (0–0) | 3  (1–5) |
| United States | 1006  (473–1769) | 2233  (912–4076) | 11 867  (5977–19 507) | 4353  (2170–7294) | 19 459  (9532–32 647) |
| **Latin America and Caribbean** | 944  (461–1492) | 3218  (1279–5754) | 9957  (4916–16 187) | 3160  (1539–5201) | 17 278  (8195–28 633) |
| Antigua and Barbuda | 0  (0–0) | 1  (0–1) | 1  (0–2) | 1  (0–1) | 2  (1–4) |
| Barbados | 1  (0–1) | 4  (1–7) | 5  (3–9) | 6  (3–10) | 15  (7–27) |
| Belize | 0  (0–1) | 2  (1–3) | 5  (2–8) | 1  (0–2) | 8  (3–14) |
| Bermuda | 0  (0–0) | 1  (0–2) | 3  (1–5) | 1  (0–2) | 5  (2–9) |
| Bolivia | 11  (5–18) | 46  (18–86) | 104  (50–174) | 51  (24–87) | 212  (96–364) |
| Brazil | 302  (150–476) | 1116  (458–1960) | 4665  (2361–7464) | 1181  (589–1916) | 7265  (3558–11 815) |
| Colombia | 60  (28–98) | 291  (110–537) | 725  (341–1215) | 265  (122–451) | 1341  (601–2300) |
| Costa Rica | 13  (6–22) | 38  (14–69) | 55  (26–92) | 37  (17–64) | 143  (63–247) |
| Cuba | 55  (25–91) | 93  (35–172) | 822  (384–1389) | 129  (59–221) | 1099  (504–1874) |
| Dominica | 0  (0–0) | 1  (0–1) | 2  (1–3) | 1  (0–2) | 4  (2–6) |
| Dominican Republic | 5  (2–8) | 37  (14–69) | 122  (57–206) | 23  (10–39) | 186  (83–322) |
| Ecuador | 16  (7–26) | 67  (25–125) | 118  (56–199) | 70  (33–120) | 270  (121–469) |
| El Salvador | 9  (4–14) | 48  (18–89) | 60  (28–101) | 21  (10–36) | 137  (59–240) |
| Grenada | 0  (0–0) | 1  (0–2) | 2  (1–3) | 2  (1–3) | 5  (2–9) |
| Guatemala | 11  (5–18) | 76  (28–144) | 81  (38–139) | 29  (13–50) | 197  (84–351) |
| Guyana | 1  (1–2) | 4  (2–8) | 7  (3–12) | 3  (1–6) | 16  (7–28) |
| Haiti | 21  (9–35) | 62  (23–118) | 134  (61–229) | 44  (20–76) | 261  (113–458) |
| Honduras | 12  (5–19) | 71  (26–132) | 60  (28–102) | 22  (10–38) | 164  (70–291) |
| Jamaica | 3  (1–4) | 22  (8–42) | 78  (36–131) | 27  (12–47) | 130  (58–224) |
| Mexico | 283  (146–427) | 793  (329–1362) | 1491  (774–2322) | 758  (386–1198) | 3325  (1634–5310) |
| Nicaragua | 3  (2–6) | 22  (8–42) | 34  (16–58) | 12  (6–21) | 72  (31–126) |
| Panama | 5  (2–9) | 24  (9–45) | 51  (24–87) | 21  (10–36) | 102  (45–177) |
| Paraguay | 8  (4–13) | 37  (14–69) | 124  (58–207) | 26  (12–44) | 195  (88–333) |
| Peru | 17  (8–28) | 115  (44–214) | 281  (134–474) | 145  (68–248) | 559  (254–964) |
| Puerto Rico | 26  (12–43) | 67  (26–122) | 130  (62–217) | 92  (43–157) | 315  (142–539) |
| Saint Lucia | 0  (0–1) | 1  (1–3) | 3  (1–5) | 2  (1–3) | 7  (3–12) |
| Saint Vincent and the Grenadines | 0  (0–0) | 1  (0–2) | 2  (1–3) | 1  (1–2) | 4  (2–8) |
| Suriname | 1  (0–2) | 3  (1–6) | 10  (5–17) | 3  (2–6) | 18  (8–31) |
| The Bahamas | 1  (0–2) | 3  (1–6) | 8  (4–14) | 4  (2–7) | 16  (7–28) |
| Trinidad and Tobago | 3  (2–5) | 11  (4–19) | 29  (14–48) | 15  (7–26) | 59  (26–99) |
| Venezuela | 74  (34–121) | 158  (59–292) | 739  (344–1242) | 163  (75–277) | 1134  (512–1932) |
| Virgin Islands, U.S. | 0  (0–1) | 3  (1–5) | 7  (3–11) | 4  (2–6) | 14  (6–23) |
| **North Africa and Middle East** | 1159  (478–1951) | 3252  (1092–6284) | 8780  (3599–15 670) | 2594  (1047–4660) | 15 785  (6216–28 565) |
| Afghanistan | 104  (45–174) | 211  (71–408) | 382  (159–675) | 77  (32–136) | 774  (307–1393) |
| Algeria | 52  (20–98) | 78  (26–164) | 194  (79–368) | 115  (46–222) | 440  (171–852) |
| Bahrain | 4  (1–7) | 8  (2–15) | 17  (5–33) | 7  (2–13) | 36  (11–68) |
| Egypt | 247  (97–421) | 434  (136–869) | 849  (330–1555) | 293  (113–540) | 1822  (675–3385) |
| Iran | 138  (64–234) | 478  (184–894) | 801  (381–1361) | 205  (96–354) | 1623  (725–2842) |
| Iraq | 61  (19–105) | 198  (49–412) | 381  (119–730) | 161  (50–308) | 801  (237–1555) |
| Jordan | 1  (0–2) | 15  (4–37) | 28  (9–65) | 14  (5–33) | 58  (18–138) |
| Kuwait | 5  (2–10) | 16  (4–33) | 23  (7–46) | 26  (8–51) | 71  (21–139) |
| Lebanon | 45  (18–81) | 45  (16–91) | 169  (71–310) | 126  (52–232) | 385  (157–714) |
| Libya | 41  (13–75) | 44  (11–95) | 152  (46–299) | 68  (21–134) | 305  (90–604) |
| Morocco | 86  (28–150) | 122  (30–255) | 811  (249–1550) | 318  (97–610) | 1336  (404–2565) |
| Oman | 7  (2–11) | 19  (5–40) | 30  (9–59) | 23  (7–45) | 79  (23–156) |
| Palestine | 6  (2–10) | 27  (6–59) | 73  (22–146) | 12  (3–23) | 117  (33–239) |
| Qatar | 2  (1–3) | 9  (2–19) | 15  (5–29) | 8  (3–16) | 34  (10–67) |
| Saudi Arabia | 56  (30–83) | 142  (63–234) | 219  (118–331) | 211  (112–323) | 628  (323–971) |
| Sudan | 90  (51–119) | 361  (163–546) | 567  (314–792) | 192  (105–270) | 1210  (633–1727) |
| Syria | 4  (1–7) | 269  (66–574) | 169  (51–333) | 34  (10–66) | 476  (129–980) |
| Tunisia | 45  (15–78) | 72  (18–149) | 430  (133–819) | 126  (39–243) | 673  (205–1289) |
| Turkey | 105  (46–180) | 531  (191–1024) | 3165  (1391–5591) | 459  (201–818) | 4260  (1829–7613) |
| United Arab Emirates | 19  (9–31) | 33  (13–61) | 52  (25–87) | 48  (22–82) | 152  (68–261) |
| Yemen | 41  (13–73) | 141  (34–304) | 251  (76–492) | 70  (21–139) | 504  (145–1007) |
| **South Asia** | 2330  (1237–3480) | 6174  (2657–10 376) | 18 820  (9692–29 667) | 6312  (3237–10 000) | 33 636  (16 823–53 523) |
| Bangladesh | 90  (42–152) | 220  (85–412) | 2774  (1340–4669) | 232  (110–398) | 3315  (1577–5632) |
| Bhutan | 0  (0–1) | 1  (0–2) | 3  (1–5) | 1  (1–2) | 6  (2–10) |
| India | 1876  (1047–2692) | 5260  (2346–8551) | 13 392  (7276–20 063) | 4929  (2672–7430) | 25 457  (13 341–38 736) |
| Nepal | 24  (12–38) | 77  (30–137) | 227  (111–372) | 64  (31–105) | 392  (184–653) |
| Pakistan | 339  (136–597) | 616  (196–1272) | 2424  (963–4558) | 1086  (424–2065) | 4465  (1718–8492) |
| **Southern Latin America** | 72  (29–138) | 221  (77–453) | 1141  (490–2113) | 299  (126–567) | 1733  (722–3270) |
| Argentina | 49  (20–93) | 146  (51–301) | 797  (342–1476) | 183  (77–347) | 1175  (490–2216) |
| Chile | 19  (8–36) | 56  (19–115) | 233  (100–433) | 92  (39–176) | 400  (166–760) |
| Uruguay | 5  (2–9) | 18  (7–37) | 111  (48–203) | 24  (10–45) | 158  (66–294) |
| **Southeast Asia, East Asia, and Oceania** | 2958  (1559–4435) | 18 368  (7963–30 795) | 149 139  (80 292–226 666) | 16 033  (8448–24 837) | 186 498  (98 261–286 733) |
| American Samoa | 0  (0–0) | 0  (0–0) | 2  (1–3) | 0  (0–1) | 2  (1–4) |
| Cambodia | 20  (9–31) | 69  (26–125) | 305  (145–502) | 51  (24–85) | 443  (205–742) |
| China | 1919  (1073–2790) | 14 398  (6514–23 464) | 125 007  (69 443–186 070) | 11 935  (6571–17 974) | 153 259  (83 601–230 298) |
| Federated States of Micronesia | 0  (0–0) | 0  (0–1) | 2  (1–3) | 0  (0–0) | 3  (1–4) |
| Fiji | 2  (1–3) | 7  (3–12) | 9  (4–14) | 4  (2–7) | 22  (10–36) |
| Guam | 1  (0–1) | 2  (1–3) | 11  (5–17) | 2  (1–3) | 15  (7–24) |
| Indonesia | 403  (201–630) | 604  (244–1068) | 2101  (1047–3397) | 1123  (554–1836) | 4231  (2045–6931) |
| Kiribati | 0  (0–0) | 0  (0–1) | 2  (1–2) | 0  (0–1) | 3  (1–4) |
| Laos | 8  (3–13) | 25  (9–50) | 113  (48–202) | 19  (8–34) | 165  (68–299) |
| Malaysia | 57  (22–97) | 244  (76–494) | 818  (317–1522) | 239  (91–447) | 1357  (506–2559) |
| Maldives | 0  (0–0) | 1  (0–3) | 3  (1–6) | 1  (0–2) | 6  (2–11) |
| Marshall Islands | 0  (0–0) | 0  (0–1) | 1  (1–2) | 0  (0–0) | 2  (1–3) |
| Mauritius | 3  (1–5) | 10  (3–19) | 33  (13–59) | 7  (3–12) | 53  (21–95) |
| Myanmar | 86  (34–145) | 304  (95–612) | 1754  (686–3231) | 232  (90–432) | 2376  (905–4420) |
| North Korea | 23  (11–37) | 177  (69–322) | 2367  (1139–3928) | 88  (42–147) | 2655  (1261–4434) |
| Northern Mariana Islands | 0  (0–0) | 1  (0–1) | 3  (1–4) | 1  (0–1) | 4  (2–7) |
| Papua New Guinea | 9  (5–14) | 35  (14–62) | 116  (58–184) | 18  (9–29) | 179  (87–290) |
| Philippines | 56  (27–86) | 616  (237–1102) | 2417  (1144–3977) | 414  (195–684) | 3502  (1603–5848) |
| Samoa | 0  (0–0) | 1  (0–1) | 1  (1–2) | 1  (0–1) | 3  (1–5) |
| Seychelles | 0  (0–1) | 1  (0–2) | 2  (1–4) | 1  (0–2) | 5  (2–9) |
| Solomon Islands | 1  (0–1) | 2  (1–4) | 8  (4–13) | 1  (1–2) | 12  (6–20) |
| Sri Lanka | 29  (12–52) | 118  (39–244) | 159  (65–297) | 68  (28–129) | 374  (143–721) |
| Taiwan | 84  (45–124) | 499  (214–832) | 2418  (1275–3702) | 601  (314–928) | 3602  (1848–5585) |
| Thailand | 118  (49–192) | 849  (273–1658) | 3666  (1462–6615) | 350  (138–634) | 4983  (1921–9099) |
| Timor-Leste | 1  (0–2) | 4  (1–8) | 12  (5–23) | 3  (1–7) | 21  (8–39) |
| Tonga | 0  (0–0) | 0  (0–1) | 3  (2–5) | 1  (0–1) | 4  (2–7) |
| Vanuatu | 0  (0–0) | 1  (0–2) | 4  (2–6) | 1  (0–1) | 6  (3–10) |
| Vietnam | 140  (64–210) | 397  (140–704) | 7803  (3421–12 875) | 872  (374–1438) | 9211  (3999–15 228) |
| **Sub-Saharan Africa** | 1010  (470–1602) | 2095  (792–3780) | 5589  (2698–9023) | 3006  (1366–5094) | 11 700  (5326–19 500) |
| Angola | 15  (7–25) | 40  (15–74) | 135  (64–224) | 56  (26–95) | 247  (112–418) |
| Benin | 5  (2–9) | 24  (8–47) | 64  (27–112) | 24  (10–43) | 118  (47–211) |
| Botswana | 2  (1–3) | 8  (3–16) | 40  (17–68) | 6  (3–11) | 56  (24–97) |
| Burkina Faso | 9  (4–15) | 31  (11–57) | 60  (26–102) | 39  (16–68) | 139  (57–242) |
| Burundi | 19  (8–30) | 21  (8–40) | 39  (17–67) | 53  (23–91) | 132  (56–228) |
| Cameroon | 12  (5–19) | 62  (22–115) | 171  (76–287) | 65  (28–112) | 310  (132–533) |
| Cape Verde | 0  (0–0) | 2  (1–4) | 8  (4–14) | 2  (1–4) | 13  (5–22) |
| Central African Republic | 8  (4–13) | 14  (5–25) | 52  (25–86) | 14  (7–24) | 88  (40–148) |
| Chad | 7  (3–11) | 29  (10–56) | 71  (30–122) | 28  (12–49) | 134  (55–238) |
| Comoros | 1  (0–1) | 1  (0–2) | 2  (1–4) | 3  (1–6) | 8  (3–14) |
| Congo | 3  (2–5) | 10  (4–17) | 36  (17–59) | 14  (7–23) | 63  (29–105) |
| Cote d'Ivoire | 17  (8–28) | 58  (20–110) | 97  (42–166) | 73  (31–129) | 245  (100–432) |
| Democratic Republic of the Congo | 56  (26–91) | 128  (48–237) | 361  (170–602) | 161  (75–272) | 706  (319–1202) |
| Djibouti | 1  (1–2) | 2  (1–4) | 5  (2–8) | 6  (3–10) | 15  (7–24) |
| Equatorial Guinea | 1  (0–1) | 2  (1–3) | 8  (4–14) | 4  (2–6) | 14  (7–24) |
| Eritrea | 8  (3–13) | 9  (3–17) | 16  (7–28) | 22  (9–39) | 54  (22–97) |
| Ethiopia | 103  (45–176) | 120  (43–233) | 282  (125–496) | 321  (139–578) | 825  (352–1483) |
| Gabon | 1  (1–2) | 4  (2–8) | 14  (7–22) | 8  (4–13) | 27  (13–45) |
| Ghana | 8  (3–13) | 46  (5–93) | 92  (38–167) | 94  (38–173) | 240  (96–445) |
| Guinea | 7  (3–12) | 24  (8–47) | 84  (35–150) | 24  (10–43) | 138  (55–251) |
| Guinea-Bissau | 1  (1–2) | 5  (2–10) | 14  (6–24) | 5  (2–9) | 26  (10–45) |
| Kenya | 39  (20–58) | 100  (42–168) | 143  (74–221) | 117  (60–183) | 398  (197–629) |
| Lesotho | 3  (1–5) | 10  (4–19) | 54  (23–92) | 5  (2–8) | 72  (30–125) |
| Liberia | 2  (1–4) | 11  (4–21) | 20  (8–34) | 10  (4–18) | 43  (18–77) |
| Madagascar | 36  (14–60) | 49  (15–95) | 82  (31–149) | 123  (46–224) | 290  (106–529) |
| Malawi | 8  (4–13) | 22  (9–39) | 47  (23–76) | 81  (38–136) | 158  (74–265) |
| Mali | 8  (4–14) | 30  (10–59) | 50  (21–89) | 30  (12–53) | 118  (47–216) |
| Mauritania | 1  (1–2) | 8  (3–16) | 20  (8–36) | 9  (4–17) | 39  (15–70) |
| Mozambique | 34  (15–57) | 45  (16–87) | 99  (43–173) | 54  (23–95) | 233  (97–412) |
| Namibia | 1  (1–2) | 5  (2–9) | 10  (4–18) | 8  (3–14) | 25  (10–43) |
| Niger | 8  (4–14) | 35  (12–68) | 75  (31–135) | 32  (13–58) | 151  (60–275) |
| Nigeria | 252  (113–398) | 373  (131–689) | 669  (292–1127) | 348  (149–596) | 1642  (685–2811) |
| Rwanda | 13  (6–21) | 20  (7–37) | 42  (18–70) | 57  (24–98) | 131  (56–226) |
| Sao Tome and Principe | 0  (0–0) | 0  (0–1) | 2  (1–4) | 0  (0–1) | 3  (1–5) |
| Senegal | 7  (3–11) | 31  (11–61) | 84  (36–148) | 31  (13–55) | 154  (62–275) |
| Sierra Leone | 4  (2–6) | 18  (6–32) | 42  (18–71) | 17  (7–30) | 81  (34–139) |
| Somalia | 16  (7–28) | 16  (5–32) | 25  (10–45) | 32  (13–59) | 89  (35–165) |
| South Africa | 91  (51–129) | 396  (179–624) | 1818  (1006–2639) | 306  (168–450) | 2612  (1404–3842) |
| South Sudan | 20  (9–32) | 24  (8–46) | 43  (19–75) | 55  (23–97) | 142  (59–250) |
| Swaziland | 1  (0–2) | 5  (2–9) | 24  (10–42) | 3  (1–5) | 33  (13–57) |
| Tanzania | 53  (25–84) | 83  (32–150) | 206  (98–340) | 237  (110–399) | 579  (265–974) |
| The Gambia | 1  (0–1) | 4  (1–7) | 8  (4–13) | 4  (2–7) | 17  (8–28) |
| Togo | 4  (2–6) | 17  (6–32) | 45  (19–80) | 17  (7–30) | 83  (33–148) |
| Uganda | 85  (45–124) | 97  (40–162) | 184  (92–282) | 254  (126–397) | 620  (302–965) |
| Zambia | 26  (13–40) | 26  (10–45) | 66  (32–105) | 89  (43–145) | 207  (98–334) |
| Zimbabwe | 10  (4–17) | 28  (10–56) | 79  (34–142) | 67  (28–123) | 184  (75–337) |
| **Western Europe** | 1790  (659–3609) | 4891  (1581–10 626) | 21 156  (8261–42 186) | 7591  (2947–15 275) | 35 428  (13 448–71 697) |
| Andorra | 0  (0–1) | 1  (0–3) | 3  (1–6) | 2  (1–4) | 7  (2–14) |
| Austria | 28  (10–56) | 130  (40–290) | 454  (168–929) | 162  (60–334) | 773  (277–1610) |
| Belgium | 49  (17–101) | 156  (47–355) | 694  (254–1449) | 196  (71–414) | 1094  (389–2319) |
| Cyprus | 4  (1–8) | 10  (3–23) | 28  (10–58) | 12  (4–25) | 54  (19–114) |
| Denmark | 24  (8–48) | 123  (37–272) | 425  (157–862) | 127  (47–261) | 699  (250–1443) |
| Finland | 18  (6–39) | 52  (16–119) | 187  (69–388) | 108  (39–228) | 366  (131–774) |
| France | 233  (80–488) | 692  (210–1568) | 2930  (1056–6118) | 968  (352–2027) | 4823  (1697–10 202) |
| Germany | 356  (125–738) | 814  (250–1854) | 4823  (1773–10 091) | 1744  (638–3678) | 7738  (2786–16 361) |
| Greece | 98  (35–201) | 133  (41–297) | 612  (226–1256) | 75  (27–157) | 918  (329–1911) |
| Iceland | 1  (0–3) | 3  (1–7) | 11  (4–24) | 4  (2–9) | 20  (7–43) |
| Ireland | 21  (7–44) | 41  (12–93) | 162  (60–336) | 70  (25–148) | 295  (105–622) |
| Israel | 12  (4–25) | 34  (10–79) | 121  (44–258) | 50  (18–108) | 218  (77–471) |
| Italy | 335  (137–625) | 889  (316–1783) | 4048  (1764–7398) | 1657  (705–3085) | 6929  (2922–12 892) |
| Luxembourg | 3  (1–6) | 9  (3–21) | 26  (10–54) | 12  (4–24) | 49  (17–105) |
| Malta | 1  (0–3) | 2  (1–6) | 13  (5–28) | 5  (2–10) | 22  (8–47) |
| Netherlands | 88  (30–184) | 249  (75–565) | 1108  (406–2298) | 372  (135–781) | 1817  (646–3827) |
| Norway | 17  (6–36) | 56  (17–128) | 202  (74–424) | 78  (28–167) | 353  (125–755) |
| Portugal | 25  (9–51) | 75  (23–167) | 340  (124–694) | 153  (56–315) | 593  (212–1227) |
| Spain | 89  (30–196) | 285  (86–663) | 1199  (441–2515) | 358  (130–766) | 1931  (687–4140) |
| Sweden | 33  (14–61) | 97  (36–188) | 352  (158–626) | 183  (81–329) | 665  (289–1203) |
| Switzerland | 28  (10–59) | 93  (28–212) | 308  (113–639) | 136  (50–285) | 565  (201–1195) |
| United Kingdom | 326  (128–636) | 946  (330–1933) | 3109  (1342–5735) | 1121  (472–2120) | 5502  (2272–10 423) |
| **SDI** |  |  |  |  |  |
| High | 3690  (1568–6815) | 11 212  (4166–21 922) | 54 014  (24 889–95 337) | 17 652  (7966–31 897) | 86 568  (38 588–155 972) |
| High-middle | 2494  (1168–4099) | 5796  (2226–10 711) | 28 039  (13 292–47 239) | 5878  (2717–10 138) | 42 208  (19 403–72 188) |
| Middle | 3974  (2036–6069) | 20 928  (8986–35 348) | 154 014  (82 858–234 181) | 18 542  (9657–28 991) | 197 458  (103 537–304 588) |
| Low-middle | 3110  (1580–4727) | 8629  (3505–14 992) | 27 074  (13 253–43 942) | 8372  (4094–13 604) | 47 184  (22 432–77 265) |
| Low | 742  (328–1214) | 1489  (514–2845) | 3302  (1431–5714) | 2084  (912–3619) | 7616  (3185–13 392) |
| **Global** |  |  |  |  |  |
|  | 14 010  (6681–22 923) | 48 053  (1939–85 817) | 266 442  (135 723–426 414) | 52 529  (25 346–88 250) | 381 035  (187 145–623 404) |

Note: TB: tuberculosis, CI: confidence interval, SDI: Socio-demographic Index.

We estimated adjusted PAF for lung cancer, stratified by smoking status (never and current/former smokers), using data published by Sun et al for 16 countries.^54^ We derived prevalence of tuberculosis for never and current/former smokers from Global Health Data Exchange (GHDx) ^55^ and Bates *et al*.^56^ The risk estimates were obtained from our meta-analysis for never-smokers (RR=1.64 [95% CI 1.41–1.91]) and Bae *et al*. ^11^ for current/former smokers (RR=1.85 [95% CI 1.08–3.19]). The cancer incidence of lung stratified by smoking status was taken from Global Health Data Exchange (GHDx) ^55^ and Sun et al.^54^ The PAF estimations with no stratification are referred to unadjusted PAF. The results of adjusted PAF for lung cancer are compared with unadjusted PAF.

The result of adjusted and unadjusted PAF of lung cancer are shown in Table S18. We observed 0.34%–3.72% point difference when adjusted PAFs were compared to unadjusted PAFs. The difference was the largest in India and the smallest in the United States.

**Table S18. Population attributable fraction (PAF) of tuberculosis on lung cancer in current and former smokers, never-smokers, and PAF adjusted and unadjusted for smoking status**

| Country | PAF, lung cancer | | | | |
| --- | --- | --- | --- | --- | --- |
|  | **Current and former smokers, %** | **Never-smokers, %** | **Unadjusted PAF, %** | **Adjusted PAF, %** | **Point difference, %** |
| China | 17.69 | 8.08 | 17.77 | 15.30 | 2.47 |
| Czech Republic | 14.75 | 6.75 | 14.97 | 14.11 | 0.86 |
| Germany | 7.76 | 3.36 | 7.88 | 7.38 | 0.49 |
| Hungary | 15.82 | 7.31 | 16.06 | 15.05 | 1.00 |
| India | 20.75 | 9.70 | 20.76 | 17.04 | 3.72 |
| Japan | 8.05 | 3.81 | 8.27 | 6.94 | 1.33 |
| Pakistan | 14.74 | 6.70 | 14.86 | 12.81 | 2.05 |
| Poland | 15.24 | 7.05 | 15.48 | 14.62 | 0.87 |
| Romania | 15.80 | 7.22 | 16.04 | 15.29 | 0.75 |
| Russian Federation | 17.88 | 8.19 | 18.14 | 17.33 | 0.81 |
| Singapore | 7.27 | 3.51 | 7.54 | 6.17 | 1.37 |
| Slovakia | 14.71 | 6.70 | 14.94 | 14.18 | 0.76 |
| South Korea | 9.90 | 4.75 | 10.18 | 8.54 | 1.64 |
| Sweden | 8.56 | 3.77 | 8.69 | 8.01 | 0.68 |
| Taiwan | 19.93 | 9.62 | 20.18 | 16.87 | 3.31 |
| United States | 4.75 | 1.96 | 4.81 | 4.47 | 0.34 |

Note: PAF: population attributable fraction.

**Table S19. Population attributable fraction (PAF) of tuberculosis on lung cancer and leukaemia in base case analyses and PAF estimations with cohort studies exclusively**

|  | **Lung cancer** | | |  | **Leukaemia** | | |
| --- | --- | --- | --- | --- | --- | --- | --- |
|  | **PAF ***  **(95% CI)** | **PAF cohort-study #**  **(95% CI)** | **Point different, %** |  | **PAF ***  **(95% CI)** | **PAF cohort-study #**  **(95% CI)** | **Point different, %** |
| **Male** |  |  |  |  |  |  |  |
| **Region** |  |  |  |  |  |  |  |
| Australasia | 6.76 (3.01–12.20) | 13.73 (4.58–27.59) | 6.96 |  | 6.67 (2.41–13.29) | 11.68 (5.06–21.39) | 5.01 |
| Central Europe, Eastern Europe, and Central Asia | 16.86 (8.37–27.31) | 30.76 (12.38–50.73) | 13.89 |  | 16.31 (6.60–28.78) | 26.47 (13.22–41.72) | 10.15 |
| High-income Asia Pacific | 8.49 (4.23–13.98) | 16.89 (6.39–30.76) | 8.40 |  | 8.79 (3.50–16.16) | 15.13 (7.25–25.47) | 6.34 |
| High-income North America | 5.45 (2.75–8.93) | 11.24 (4.20–21.30) | 5.79 |  | 5.32 (2.18–9.69) | 9.43 (4.58–16.03) | 4.11 |
| Latin America and Caribbean | 16.40 (8.12–26.56) | 30.03 (12.03–49.72) | 13.64 |  | 15.89 (6.34–28.34) | 25.86 (12.72–41.20) | 9.97 |
| North Africa and Middle East | 16.75 (6.81–29.95) | 30.36 (10.13–53.40) | 13.62 |  | 16.41 (5.52–31.65) | 26.38 (11.08–44.62) | 9.97 |
| South Asia | 17.54 (9.01–27.69) | 31.54 (13.23–50.88) | 14.00 |  | 19.85 (8.60–33.10) | 31.35 (16.82–46.77) | 11.50 |
| Southern Latin America | 7.50 (3.30–13.64) | 15.11 (5.02–30.29) | 7.61 |  | 6.96 (2.49–14.00) | 12.16 (5.23–22.43) | 5.20 |
| Southeast Asia, East Asia, and Oceania | 18.50 (10.00–28.00) | 33.27 (14.67–51.73) | 14.77 |  | 18.24 (7.96–30.36) | 29.25 (15.73–43.69) | 11.01 |
| Sub-Saharan Africa | 21.91 (10.60–35.22) | 37.87 (15.44–59.70) | 15.96 |  | 19.72 (7.46–35.48) | 31.07 (14.72–49.25) | 11.36 |
| Western Europe | 7.83 (3.05–15.61) | 15.66 (4.65–33.49) | 7.83 |  | 7.36 (2.38–15.94) | 12.78 (4.99–25.10) | 5.43 |
| **SDI** |  |  |  |  |  |  |  |
| High | 8.02 (3.69–14.15) | 15.89 (5.58–30.58) | 7.87 |  | 7.63 (2.84–14.89) | 13.15 (5.90–23.43) | 5.52 |
| High-middle | 14.47 (6.87–24.34) | 26.67 (10.19–46.01) | 12.20 |  | 13.38 (5.13–24.72) | 21.93 (10.35–36.30) | 8.55 |
| Middle | 18.40 (9.94–27.85) | 33.11 (14.59–51.51) | 14.70 |  | 17.87 (7.72–29.99) | 28.69 (15.27–43.20) | 10.82 |
| Low-middle | 17.95 (8.76–29.17) | 32.18 (12.88–52.65) | 14.24 |  | 19.75 (8.09–34.04) | 31.18 (15.86–47.73) | 11.43 |
| Low | 19.01 (8.22–32.84) | 33.90 (12.16–57.15) | 14.89 |  | 17.97 (6.24–34.08) | 28.75 (12.51–47.71) | 10.78 |
| **Global** |  |  |  |  |  |  |  |
|  | 14.44 (7.38–23.01) | 26.52 (10.90–44.01) | 12.09 |  | 13.35 (5.42–23.71) | 21.78 (10.84–34.87) | 8.44 |
| **Female** |  |  |  |  |  |  |  |
| **Region** |  |  |  |  |  |  |  |
| Australasia | 6.75 (2.78–12.94) | 13.67 (4.23–28.79) | 6.92 |  | 6.75 (2.78–12.94) | 13.67 (4.23–28.79) | 6.92 |
| Central Europe, Eastern Europe, and Central Asia | 15.36 (7.61–24.98) | 28.44 (11.31–47.66) | 13.07 |  | 15.36 (7.61–24.98) | 28.44 (11.31–47.66) | 13.07 |
| High-income Asia Pacific | 9.04 (4.51–14.81) | 17.82 (6.81–32.02) | 8.78 |  | 9.04 (4.51–14.81) | 17.82 (6.81–32.02) | 8.78 |
| High-income North America | 4.00 (2.00–6.64) | 8.37 (3.06–16.37) | 4.38 |  | 4.00 (2.00–6.64) | 8.37 (3.06–16.37) | 4.38 |
| Latin America and Caribbean | 15.61 (7.67–25.53) | 28.81 (11.39–48.33) | 13.19 |  | 15.61 (7.67–25.53) | 28.81 (11.39–48.33) | 13.19 |
| North Africa and Middle East | 16.25 (6.84–28.76) | 29.54 (10.16–51.90) | 13.30 |  | 16.25 (6.84–28.76) | 29.54 (10.16–51.90) | 13.30 |
| South Asia | 16.66 (8.63–26.14) | 30.23 (12.71–48.92) | 13.57 |  | 16.66 (8.63–26.14) | 30.23 (12.71–48.92) | 13.57 |
| Southern Latin America | 7.33 (2.99–14.04) | 14.75 (4.55–30.78) | 7.42 |  | 7.33 (2.99–14.04) | 14.75 (4.55–30.78) | 7.42 |
| Southeast Asia, East Asia, and Oceania | 17.00 (9.06–26.12) | 31.01 (13.35–49.28) | 14.02 |  | 17.00 (9.06–26.12) | 31.01 (13.35–49.28) | 14.02 |
| Sub-Saharan Africa | 20.29 (9.73–33.07) | 35.59 (14.24–57.32) | 15.31 |  | 20.29 (9.73–33.07) | 35.59 (14.24–57.32) | 15.31 |
| Western Europe | 7.10 (2.77–14.13) | 14.30 (4.23–30.86) | 7.21 |  | 7.10 (2.77–14.13) | 14.30 (4.23–30.86) | 7.21 |
| **SDI** |  |  |  |  |  |  |  |
| High | 6.55 (3.02–11.56) | 13.10 (4.58–25.59) | 6.56 |  | 6.76 (2.51–13.27) | 11.68 (5.21–20.99) | 4.92 |
| High-middle | 13.11 (6.19–22.22) | 24.52 (9.22–43.15) | 11.41 |  | 12.89 (4.96–23.81) | 21.20 (10.02–35.14) | 8.31 |
| Middle | 16.90 (8.99–26.02) | 30.85 (13.26–49.11) | 13.94 |  | 16.55 (7.04–28.24) | 26.83 (14.03–41.11) | 10.28 |
| Low-middle | 16.88 (8.35–27.28) | 30.59 (12.30–50.36) | 13.71 |  | 18.24 (7.32–32.04) | 29.10 (14.47–45.46) | 10.87 |
| Low | 17.84 (7.77–31.00) | 32.15 (11.52–54.99) | 14.31 |  | 17.17 (5.88–33.11) | 27.65 (11.85–46.63) | 10.47 |
| **Global** |  |  |  |  |  |  |  |
|  | 11.98 (6.05–19.37) | 22.41 (8.98–38.23) | 10.43 |  | 11.98 (6.05–19.37) | 22.41 (8.98–38.23) | 10.43 |
| **Both** |  |  |  |  |  |  |  |
| **Region** |  |  |  |  |  |  |  |
| Australasia | 6.76 (2.92–12.50) | 13.70 (4.44–28.08) | 6.95 |  | 6.60 (2.33–13.42) | 11.55 (4.89–21.54) | 4.95 |
| Central Europe, Eastern Europe, and Central Asia | 16.52 (8.19–26.77) | 30.22 (12.13–50.03) | 13.70 |  | 15.92 (6.44–28.10) | 25.90 (12.92–40.91) | 9.98 |
| High-income Asia Pacific | 8.66 (4.31–14.23) | 17.17 (6.52–31.15) | 8.51 |  | 9.20 (3.67–16.84) | 15.76 (7.59–26.36) | 6.56 |
| High-income North America | 4.78 (2.40–7.87) | 9.91 (3.67–19.01) | 5.13 |  | 4.72 (1.92–8.63) | 8.40 (4.06–14.38) | 3.67 |
| Latin America and Caribbean | 16.09 (7.94–26.15) | 29.55 (11.77–49.17) | 13.46 |  | 15.77 (6.27–28.20) | 25.67 (12.59–41.01) | 9.90 |
| North Africa and Middle East | 16.64 (6.82–29.69) | 30.19 (10.14–53.08) | 13.55 |  | 16.41 (5.51–31.71) | 26.39 (11.07–44.72) | 9.98 |
| South Asia | 17.32 (8.92–27.31) | 31.22 (13.11–50.40) | 13.90 |  | 19.08 (8.21–32.06) | 30.29 (16.11–45.58) | 11.22 |
| Southern Latin America | 7.44 (3.20–13.78) | 14.99 (4.86–30.45) | 7.55 |  | 6.86 (2.39–14.09) | 11.99 (5.01–22.52) | 5.13 |
| Southeast Asia, East Asia, and Oceania | 18.07 (9.73–27.46) | 32.62 (14.29–51.03) | 14.56 |  | 17.66 (7.65–29.60) | 28.42 (15.17–42.79) | 10.77 |
| Sub-Saharan Africa | 21.38 (10.32–34.53) | 37.13 (15.05–58.93) | 15.75 |  | 19.40 (7.34–35.01) | 30.65 (14.50–48.74) | 11.25 |
| Western Europe | 7.58 (2.96–15.12) | 15.21 (4.51–32.61) | 7.63 |  | 6.87 (2.22–14.94) | 11.99 (4.67–23.66) | 5.11 |
| **SDI** |  |  |  |  |  |  |  |
| High | 7.46 (3.44–13.16) | 14.82 (5.20–28.67) | 7.37 |  | 7.27 (2.70–14.21) | 12.53 (5.61–22.41) | 5.26 |
| High-middle | 14.17 (6.72–23.87) | 26.19 (9.97–45.38) | 12.02 |  | 13.17 (5.06–24.34) | 21.62 (10.21–35.82) | 8.45 |
| Middle | 17.97 (9.67–27.32) | 32.45 (14.20–50.81) | 14.48 |  | 17.34 (7.44–29.28) | 27.94 (14.77–42.36) | 10.60 |
| Low-middle | 17.67 (8.65–28.68) | 31.78 (12.73–52.07) | 14.10 |  | 19.10 (7.76–33.18) | 30.28 (15.26–46.75) | 11.19 |
| Low | 18.63 (8.08–32.25) | 33.34 (11.95–56.46) | 14.71 |  | 17.59 (6.07–33.61) | 28.22 (12.19–47.20) | 10.63 |
| **Global** |  |  |  |  |  |  |  |
|  | 13.66 (6.96–21.86) | 25.22 (10.29–42.19) | 11.56 |  | 12.90 (5.21–23.03) | 21.11 (10.44–33.97) | 8.21 |

Note: PAF: population attributable fraction. * Proportion of cancers attributable to tuberculosis were calculated using pooled estimates from case-control and cohort studies. # Proportion of cancers attributable to tuberculosis were calculated using pooled estimates from cohort studies.

**References**

1. World Health Organization. International Statistical Classification of Diseases and Related Health Problems 10th Revision: ICD-10 Version: 2015. Geneva: World Health Organization. 2015.
2. Wells G, Shea B, O’Connell D, Peterson J, Welch V, Losos M, et al. The Newcastle-Ottawa Scale (NOS) for assessing the quality of non-randomized studies in meta-analyses. 2017. http://www.ohri.ca/programs/clinical_epidemiology/oxford.asp. Accessed 15 Sept 2018.
3. Hartling L, Hamm M, Milne A, et al. Validity and inter-rater reliability 32 testing of quality assessment instruments: methods research reports. Report No. 12-EHC039-EF. Rockville, MD: Agency for Healthcare Research and Quality (US). 2012.
4. Global Burden of Disease Collaborative Network. Global Burden of Disease Study 2016 (GBD 2016) Socio-demographic Index (SDI) 1970–2016. Seattle, United States: Institute for Health Metrics and Evaluation (IHME). 2017. Accessed 12 Jan 2018.
5. The World Bank. World Bank analytical classifications. 2015. https://datahelpdesk.worldbank.org/knowledgebase/articles/906519-world-bank-country-and-lending-groups. Accessed 16 Dec 2016.
6. Everatt R, Kuzmickiene I, Davidaviciene E, Cicenas S. Non-pulmonary cancer risk following tuberculosis: a nationwide retrospective cohort study in Lithuania. Infect Agent Cancer. 2017;12:33.
7. Everatt R, Kuzmickiene I, Davidaviciene E, Cicenas S. Incidence of lung cancer among patients with tuberculosis: a nationwide cohort study in Lithuania. Int J Tuberc Lung Dis. 2016;20:757-763.
8. Hong S, Mok Y, Jeon C, Jee SH, Samet JM. Tuberculosis, smoking and risk for lung cancer incidence and mortality. Int J Cancer. 2016;139:2447-2455.
9. Simonsen DF,  Farkas DK, Søgaard M, Horsburgh CR, Sørensen HT, Thomsen RW. Tuberculosis and risk of cancer: a Danish nationwide cohort study. Int J Tuberc Lung Dis. 2014;18:1211-1219.
10. Kuo SC, Hu YW, Liu CJ, Lee YT, Chen YT, Chen TL, et al. Association between tuberculosis infections and non-pulmonary malignancies: a nationwide population-based study. BJC. 2013;109:229-234.
11. Bae JM, Li ZM, Shin MH, Kim DH, Lee MS, Ahn YO. Pulmonary tuberculosis and lung cancer risk in current smokers: the Seoul Male Cancer Cohort Study. J Korean Med Sci. 2013;28:896-900.
12. Shiels MS, Albanes D, Virtamo J, Engels EA. Increased risk of lung cancer in men with tuberculosis in the alpha-tocopherol, beta-carotene cancer prevention study. Cancer Epidemiol Biomarkers Prev. 2011;20:672-678.
13. Fan YG, Jiang Y, Chang RS, Yao SX, Jin P, Wang W, et al. Prior lung disease and lung cancer risk in an occupational-based cohort in Yunnan, China. Lung Cancer. 2011;72:258-263.
14. Yu YH, Liao CC, Hsu WH, Chen HJ, Liao WC, Muo CH, et al. Increased lung cancer risk among patients with pulmonary tuberculosis: a population cohort study. J Thorac Oncol. 2011;6:32-37.
15. Littman AJ, Thornquist MD, White E, Jackson LA, Goodman GE, Vaughan TL. Prior lung disease and risk of lung cancer in a large prospective study. Cancer Causes Control. 2004;15:819-827.
16. Askling J, Ekbom A. Risk of non-Hodgkin's lymphoma following tuberculosis. Br J Cancer. 2001;84:113-115.
17. Yang L, Lu X, Deng J, Zhou Y, Huang D, Qiu F, et al. Risk factors shared by COPD and lung cancer and mediation effect of COPD: two center case–control studies. Cancer Causes Control. 2015;26:11-24.
18. HosgoodIII HD, Chapman RS, He X, Hu W, Tian L, Liu LZ, et al. History of lung disease and risk of lung cancer in a population with high household fuel combustion exposures in rural China. Lung Cancer. 2013;81:343-846.
19. Fan R, Zhang LY, Wang H, Yang B, Han T, Zhao XL, et al. Multicentre hospital-based case-control study of diffuse large B-cell lymphoma in shanghai, China. Asian Pac J Cancer Prev. 2012;13:3329-3334.
20. Park SK, Cho LY, Yang JJ, Park B, Chang SH, Lee KS, et al. Lung cancer risk and cigarette smoking, lung tuberculosis according to histologic type and gender in a population based case-control study. Lung Cancer. 2010;68:20-26.
21. Koshiol J, Rotunno M, Consonni D, Pesatori AC, De Matteis S, Goldstein AM, et al. Lower risk of lung cancer after multiple pneumonia diagnoses. Cancer Epidemiol Biomarkers Prev. 2010;19:716-721.
22. Brenner DR, Hung RJ, Tsao MS, Shepherd FA, Johnston MR, Narod S, et al. Lung cancer risk in never-smokers: a population-based case-control study of epidemiologic risk factors. BMC Cancer. 2010;10:285.
23. Liang H, Guan P, Yin Z, Li X, He Q, Zhou B. Risk of lung cancer following nonmalignant respiratory conditions among nonsmoking women living in Shenyang, Northeast China. J Womens Health (Larchmt). 2009;18:1989-1995.
24. Wang XR, Ignatius TS, Chiu YL, Qiu H, Fu Z, Goggins W, et al. Previous pulmonary disease and family cancer history increase the risk of lung cancer among Hong Kong women. Cancer Causes Control. 2009;20:757-763.
25. Galeone C, Pelucchi C, La Vecchia C, Negri E, Bosetti C, Hu J. Indoor air pollution from solid fuel use, chronic lung diseases and lung cancer in Harbin, Northeast China. Eur J Cancer Prev. 2008;17:473-478.
26. Ramanakumar AV, Parent ME, Menzies D, Siemiatycki J. Risk of lung cancer following nonmalignant respiratory conditions: evidence from two case-control studies in Montreal, Canada. Lung Cancer. 2006;53:5-12.
27. Zatloukal P, Pauk N, Tomášek L, Petruzelka L. Adenocarcinoma of the lung among women: risk associated with smoking, prior lung disease, diet and menstrual and pregnancy history. Lung Cancer. 2003;41:283-293.
28. Chan-Yeung M, Koo LC, Ho JM, Tsang KT, Chau WS, Chiu SW, et al. Risk factors associated with lung cancer in Hong Kong. Lung Cancer. 2003;40:131-140.
29. Kreuzer M, Heinrich J, Kreienbrock L, Rosario AS, Gerken M, Wichmann HE. Risk factors for lung cancer among nonsmoking women. Int J Cancer. 2002;100:706-713.
30. Brenner AV, Wang Z, Kleinerman RA, Wang L, Zhang S, Metayer C, et al. Previous pulmonary diseases and risk of lung cancer in Gansu Province, China. Int J Epidemiol. 2001;30:118-124.
31. Lee CH, Ko YC, Cheng LS, Lin YC, Lin HJ, Huang MS, et al. The heterogeneity in risk factors of lung cancer and the difference of histologic distribution between genders in Taiwan. Cancer Causes Control. 2001;12:289-300.
32. Kreuzer M, Gerken M, Kreienbrock L, Wellmann J, Wichmann HE. Lung cancer in lifetime nonsmoking men–results of a case-control study in Germany. Br J Cancer. 2001;84:134-140.
33. Brownson RC, Alavanja MC. Previous lung disease and lung cancer risk among women (United States). Cancer Causes Control. 2000;11:853-858.
34. Osann KE, Lowery JT, Schell MJ. Small cell lung cancer in women: risk associated with smoking, prior respiratory disease, and occupation. Lung Cancer. 2000;28:1-10.
35. Vineis P, Crosignani P, Sacerdote C, Fontana A, Masala G, Miligi L, et al. Haematopoietic cancer and medical history: a multicentre case control study. J Epidemiol Community Health. 2000;54:431-436.
36. Tavani A, La Vecchia C, Franceschi S, Serraino D, Carbone A. Medical history and risk of Hodgkin's and non-Hodgkin's lymphomas. Eur J Cancer Prev. 2000;9:59-64.
37. Kölmel KF, Pfahlberg A, Mastrangelo G, Niin M, Botev IN, Seebacher C, et al. Infections and melanoma risk: results of a multicentre EORTC case-control study. Melanoma Res. 1999;9:511-519.
38. Ko YC, Lee CH, Chen MJ, Huang CC, Chang WY, Lin HJ, et al. Risk factors for primary lung cancer among non-smoking women in Taiwan. Int J Epidemiol. 1997;26:24-31.
39. Schwartz AG, Yang P, Swanson GM. Familial risk of lung cancer among nonsmokers and their relatives. Am J Epidemiol. 1996;144:554-562.
40. Wu AH, Fontham ET, Reynolds P, Greenberg RS, Buffler P, Liff J, et al. Previous lung disease and risk of lung cancer among lifetime non-smoking women in the United States. Am J Epidemiol. 1995;141:1023-1032.
41. Zheng W, Linet MS, Shu XO, Pan RP, Gao YT, Fraumeni JF Jr. Prior medical conditions and the risk of adult leukemia in Shanghai, People's Republic of China. Cancer Causes Control. 1993;4:361-368.
42. Ger LP, Hsu WL, Chen KT, Chen CJ. Risk factors of lung cancer by histological category in Taiwan. Anticancer Res. 1993;13:1491-1500.
43. Alavanja MC, Brownson RC, Boice JD, Hock E. Preexisting lung disease and lung cancer among nonsmoking women. Am J Epidemiol. 1992;136:623-632.
44. Doody MM, Linet MS, Glass AG, Friedman GD, Pottern LM, Boice JD, et al. Leukemia, lymphoma, and multiple myeloma following selected medical conditions. Cancer Causes Control. 1992;3:449-456.
45. Rosenblatt KA, Koepsell TD, Daling JR, Lyon JL, Swanson GM, Greenberg RS, et al. Antigenic stimulation and the occurrence of chronic lymphocytic leukemia. Am J Epidemiol. 1991;134:22-28.
46. Gramenzi A, Buttino I, D'Avanzo B, Negri E, Franceschi S, La Vecchia C. Medical history and the risk of multiple myeloma. Br J Cancer. 1991;63:769-772.
47. Wu-Williams AH, Dai XD, Blot W, Xu ZY, Sun XW, Xiao HP, et al. Lung cancer among women in north-east China. Br J Cancer. 1990;62:982-987.
48. Wu AH, Mimi CY, Thomas DC, Pike MC, Henderson BE. Personal and family history of lung disease as risk factors for adenocarcinoma of the lung. Cancer Res. 1988;48:7279-7284.
49. Zheng W, Blot WJ, Liao ML, Wang ZX, Levin LI, Zhao JJ, et al. Lung cancer and prior tuberculosis infection in Shanghai. Br J Cancer. 1987;56:501-504.
50. Samet JM, Humble CG, Pathak DR. Personal and family history of respiratory disease and lung cancer risk. Am Rev Respir Dis. 1986;134:466­-470.
51. Bernard SM, Cartwright RA, Bird CC, Richards ID, Lauder I, Roberts BE. Aetiologic factors in lymphoid malignancies: a case-control epidemiological study. Leuk Res. 1984;8:681-689.
52. Hinds MW, Cohen HI, Kolonel LN. Tuberculosis and lung cancer risk in nonsmoking women. Am Rev of Respir Dis. 1982;125:776-778.
53. Egger M, Davey Smith G, Schneider M, Minder C. Bias in meta-analysis detected by a simple, graphical test. BMJ. 1997;315:629-634.
54. Sun S, Schiller JH, Gazdar AF. Lung cancer in never smoker-a different disease. Net Rev Cancer. 2007;7:778-790.
55. Global Burden of Disease Collaborative Network. Global Burden of Disease Study 2016 (GBD 2016) Results. Seattle, United States: Institute for Health Metrics and Evaluation (IHME). 2007. http://ghdx.healthdata.org/gbd-2016. Accessed 20 Oct 2017
56. Bates MN, Khalakdina A, Pai M, Chang L, Lessa F, Smith KR. Risk of tuberculosis from exposure to tobacco smoke: a systematic review and meta-analysis. Arch Intern Med. 2007;167:335-342.
